# Persistent Organic Pollutants and Breast Cancer: A Systematic Review and Critical Appraisal of the Literature

**DOI:** 10.3390/cancers11081063

**Published:** 2019-07-27

**Authors:** Kaoutar Ennour-Idrissi, Pierre Ayotte, Caroline Diorio

**Affiliations:** 1 Axe Oncologie, Centre de Recherche du CHU de Québec-Université Laval, Quebec City, QC G1E 6W2, Canada; 2 Centre de Recherche sur le Cancer, Université Laval, Quebec City, QC G1R 3S3, Canada; 3 Département de Médecine Sociale et Préventive, Faculté de Médecine, Université Laval, Quebec City, QC G1V 0A6, Canada; 4 Centre des Maladies du Sein Deschênes-Fabia, Hôpital du Saint-Sacrement, Quebec City, QC G1S 4L8, Canada; 5 Axe santé des Populations et Pratiques Optimales en Santé, Centre de Recherche du CHU de Québec, Université Laval, Quebec City, QC G1E 6W2, Canada; 6 Centre de Toxicologie du Québec (CTQ), INSPQ, Quebec City, QC G1V 5B3, Canada

**Keywords:** breast cancer, persistent organic pollutants, breast cancer risk, breast cancer prognostic, systematic review

## Abstract

Persistent organic pollutants (POPs) bioaccumulate in the food chain and have been detected in human blood and adipose tissue. Experimental studies demonstrated that POPs can cause and promote growth of breast cancer. However, inconsistent results from epidemiological studies do not support a causal relationship between POPs and breast cancer in women. To identify individual POPs that are repeatedly found to be associated with both breast cancer incidence and progression, and to demystify the observed inconsistencies between epidemiological studies, we conducted a systematic review of 95 studies retrieved from three main electronic databases. While no clear pattern of associations between blood POPs and breast cancer incidence could be drawn, POPs measured in breast adipose tissue were more clearly associated with higher breast cancer incidence. POPs were more consistently associated with worse breast cancer prognosis whether measured in blood or breast adipose tissue. In contrast, POPs measured in adipose tissue other than breast were inversely associated with both breast cancer incidence and prognosis. Differences in biological tissues used for POPs measurement and methodological biases explain the discrepancies between studies results. Some individual compounds associated with both breast cancer incidence and progression, deserve further investigation.

## 1. Introduction

Persistent organic pollutants (POPs) are a group of chemical substances of synthetic origin used for industrial, agricultural or domestic purposes, that persist in the environment and bioaccumulate in the food chain due to their lipophilic properties [1,2]. POPs have been detected in human blood, adipose tissue and human milk and have been linked to the increase in the incidence of hormone-dependent breast cancers [1,3,4,5,6,7,8]. 

Given the abundance of adipose tissue in the human breast, mammary epithelial cells exposure to POPs sequestered in breast adipose tissue may promote carcinogenesis and progression of mammary cancers [9]. In fact, numerous in vitro studies have demonstrated that some POPs stimulate the growth of estrogen receptor (ER)-positive breast cancer cells [10,11,12]. In animal studies, exposure to some POPs, particularly during the perinatal period, impairs breast tissue development and increases its susceptibility to carcinogens and the incidence of precancerous and cancerous breast lesions [13]. In addition to their endocrine disrupting effect either as agonists or as antagonists of endogenous hormones [14], POPs can interfere with estrogen synthesis by disrupting adipose tissue functioning [15,16], interact with transcription factors [17], induce genotoxic enzymes [17] and cytochrome 450 leading to increased levels of reactive oxygen species [18], and induce trans-generational phenotypic changes by altering the epigenome [19]. 

Although experimental studies demonstrate that POPs can cause and promote growth of breast cancer, several observational studies conducted in humans yielded inconsistent results regarding the implication of POPs in women breast cancers [20,21,22,23,24,25,26]. Observational studies are known to be prone to different biases that vary according to studies designs [27]. To draw meaningful conclusions about a causal relationship between POPs and breast cancer in women, a systematic comparison of the strengths and weaknesses of studies should be performed to triangulate their findings to provide assurance that the observed findings are actually real [27]. Thus, the objective of the present systematic review of the literature was to evaluate the observed associations between POPs and breast cancer risk and prognosis to identify individual POPs that are repeatedly found to be associated with both breast cancer incidence and progression, and to provide an explanation to the observed inconsistencies between studies.

## 2. Materials and Methods 

A systematic review was conducted following a pre-established protocol and according to the general methodology of Cochrane reviews [28]. Considering the expected methodological diversity and heterogeneity between eligible studies, the great susceptibility of observational designs to selection bias and the variability in methods used to control for confounding, no quantitative synthesis was planned [28].

### 2.1. Search Methods for Identification of Studies

An electronic search of the following databases was performed, from inception to December 2018: MEDLINE (via PubMed), EMBASE and CENTRAL (Cochrane Central Register of Controlled Trials). Search strategies were developed for each of these databases with text words and index terms referring to POPs, breast cancer risk and breast cancer prognosis, and excluding animal studies (Table S1). No language or publication date restrictions were applied. Reference lists of relevant reviews and of included studies were scanned for any additional relevant studies not otherwise identified.

### 2.2. Criteria for Considering Studies for This Review

#### 2.2.1. Types of Studies

Any observational or intervention study that evaluated the association between POPs and breast cancer risk, survival or a meaningful breast cancer prognostic factor, whatever the design was eligible for inclusion. No restrictions were applied regarding language or type (articles, short reports and abstracts) of publication. 

#### 2.2.2. Types of Participants

Women included in the studies before or after breast cancer diagnosis, regardless of age, menopausal status, breast cancer type, disease stage and treatment regimen, were eligible. No participants were excluded based on ethnicity.

#### 2.2.3. Types of Exposures 

Studies that measured exposure to any lipophilic POP, in a lipid rich biological human sample (peripheral blood and adipose tissue), whatever the method of measurement, were eligible.

#### 2.2.4. Types of Outcomes 

Breast cancer risk, measured by breast cancer incidence, prevalence or breast mammographic density (a recognized breast cancer risk factor) and breast cancer survival, including overall survival (all-cause mortality), breast cancer-specific survival (breast cancer-specific mortality), and breast cancer-free survival (breast cancer recurrence), were the primary outcomes. Studies that assessed the association of POPs with meaningful breast cancer prognostic factors (age, stage, tumor size, lymph node involvement, histological type, grade and molecular subtype) were also eligible.

### 2.3. Data Collection and Analysis

#### 2.3.1. Selection of Studies

The references identified by the search strategy were reviewed by one author (K.E-I.) in a two-step process. First, the title and abstract of each study were screened to exclude obviously non-eligible studies and second, the full text of retained articles was examined and subjected to evaluation using the predefined eligibility criteria. Whenever required, a second review author (C.D.) was consulted. When required, further information was sought from the authors by email.

#### 2.3.2. Data Extraction

Data extraction was performed using an exhaustive standardized form designed for this review. Information about study design (inclusion criteria, sample size and methodology), participants and tumors characteristics at diagnosis (age, menopausal status, tumor invasiveness, tumor ER status), exposure assessment (timing, tissue sample, method of measurement, lipid-adjustment, list of all contaminants evaluated, treatment of non-detectable values), measured outcomes and reported results (any reported measure of association, adjustment variables, and statistical model selection procedure) were collected. For observational studies, special attention was paid to distinguishing between adjusted and unadjusted results, and to the variable selection method used in multivariate analyses. Studies definition of each characteristic or variable retained was recorded. In the case of multiple publications related to the same study, the publication reporting the outcomes of interest to the present review or the one with the longest follow-up of these outcomes was considered as the reference, and information was supplemented by secondary publications as required. Abstracts with insufficient information and data to permit inclusion were excluded from the qualitative synthesis (Table S2). Data were extracted twice over the course of several days to ensure their consistency.

#### 2.3.3. Assessment of Risk of Bias in Retained Studies

Based on the “STrengthening the Reporting of OBservational studies in Epidemiology.” (STROBE) statements [28], and the rating approach of the “Risk Of Bias in Non-randomized Studies-of Interventions” (ROBINS-I) tool [27], the following domains were evaluated for risk of bias of included studies: selection of participants into the study, exposure measurement, outcome measurement, potential confounding accounted for, missing data, and selective reporting. 

Assessment of the risk of bias was performed twice by a review author (K.E-I.), both for the risk of bias in each study and for the overall risk of bias across studies. When required, a second reviewer (C.D.) was consulted.

#### 2.3.4. Assessment of Heterogeneity

Differences between studies, including study design, participant characteristics (age and menopausal status), tumor characteristics (invasiveness, ER status, and treatment received), exposure measurement (timing, type of tissue sample) and different levels of risk of bias were considered for exploring possible sources of heterogeneity. 

#### 2.3.5. Data Synthesis

Given that high heterogeneity between studies was expected, quantitative synthesis of data was not considered appropriate. A systematic qualitative synthesis of study characteristics and results was performed for risk, mortality, and prognostic factors associations with POPs exposure, and separately for each type of tissue sample. The results were considered adjusted only when all important confounders were considered into the models. For breast cancer risk, authors should have considered at minimum age, body mass index or any other estimation of body fat, and breastfeeding or parity as potential confounders. For breast cancer mortality, authors should have adjusted at minimum for age. In addition, studies of breast adipose POPs should have considered breastfeeding or parity as potential confounders. A positive association was defined as an observed higher risk or mortality with higher POPs exposure whereas a negative association was defined as an observed inverse association.

## 3. Results

### 3.1. Results of the Search

**Figure 1:**
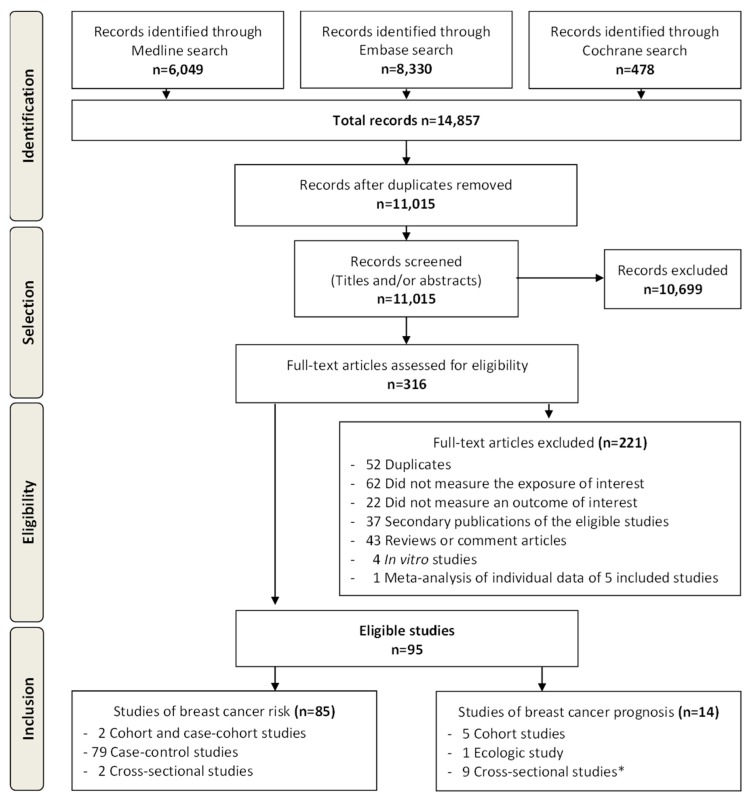
Flow Diagram according to PRISMA (Preferred Reporting Items of Systematic Reviews and Meta-Analyses) [PRISMA], with modifications. *One cohort study on breast cancer mortality also reported cross-sectional analyses of prognostic factors.

Of the 11,015 references retrieved by electronic search, 95 met eligibility criteria (Figure 1), of which 85 reported breast cancer incidence or prevalence outcomes [29,30,31,32,33,34,35,36,37,38,39,40,41,42,43,44,45,46,47,48,49,50,51,52,53,54,55,56,57,58,59,60,61,62,63,64,65,66,67,68,69,70,71,72,73,74,75,76,77,78,79,80,81,82,83,84,85,86,87,88,89,90,91,92,93,94,95,96,97,98,99,100,101,102,103,104,105,106,107,108,109,110,111,112,113], six reported mortality outcomes [41,45,114,115,116,117] and nine reported breast cancer prognostic factors [66,90,116,118,119,120,121,122,123]. The majority of studies of breast cancer risk were case-control studies (*n* = 81) whereas studies of breast cancer prognosis included five cohort studies for mortality and nine cross-sectional studies for breast cancer prognostic factors. Overall, POPs were measured in peripheral blood in 63 studies, in breast adipose tissue in 32 studies, in adipose tissue other than breast in five studies and in breast tumors in four studies (Figure 1).

### 3.2. Description of Studies

The 95 included studies were published between 1976 and 2018, and involved between five and 902 breast cancer patients (median = 113).

#### 3.2.1. Studies of Breast Cancer Risk

Characteristics of the 61 studies that examined associations between peripheral blood POPs and breast cancer risk are summarized in Table 1. These studies included breast cancer patients between 40 and 66 years of mean age with varying proportions of premenopausal and postmenopausal patients. Ten studies included at least 80% of postmenopausal patients, of which three studies included exclusively postmenopausal patients. The proportion of invasive breast cancers was not reported in 35 studies and varied in the remaining 26 studies between 62 and 100%. Twenty studies included at least 80% of invasive breast cancers of which 13 studies included exclusively invasive breast cancers. The proportion of estrogen receptor (ER) positive breast cancers was not reported in 40 studies and varied in the remaining 19 studies between 32% and 87%. Two studies included at least 80% of ER-positive breast cancers (Table 1 and Table S3). 

Characteristics of the 26 studies that examined associations between breast adipose tissue POPs and breast cancer risk are summarized in Table 2. All these studies were hospital-based case-control studies with hospital-controls and included breast cancer patients between 40 and 63 years of mean age with varying proportions of premenopausal and postmenopausal patients. Only one study included at least 80% of postmenopausal patients. The proportion of invasive breast cancers was not reported in 11 studies and varied in the remaining 15 studies between 76% and 100%, with eight studies including exclusively invasive breast cancers. The proportion of ER-positive breast cancers was not reported in 15 studies and varied in the remaining 11 studies between 45% and 90%. No study included at least 80% of ER-positive breast cancers (Table 2 and Supplementary Table S4).

Four studies examined associations between POPs in breast tumors and breast cancer risk, of which three included breast tissue surrounding malignant tumors for cases. Two studies compared malignant tumors of cases to benign tumors of controls whereas the two other studies compared malignant tumors of cases to normal tissue of controls. All these four studies were hospital-bases case-control studies with hospital controls, in which cases were on average 50 to 60 years old in the two studies that reported age at diagnosis. One study included exclusively invasive cancers, whereas the three other studies did not report proportion of invasive cancers. No study reported proportion of menopausal patients or the proportion of ER-positive breast cancers (Table S4).

Two studies examined associations between POPs in buttock adipose tissue and breast cancer risk. The recent one was a cohort-nested case-control study with incidence density (risk-set) sampling and included 409 postmenopausal breast cancer patients aged 58 years old in average, of which 78% had ER-positive breast cancers. The proportion of invasive tumors was not reported. The other study was a hospital-based case-control study with community controls including 265 breast cancer patients aged 62 years old in average. The proportions of postmenopausal patients, invasive tumors and ER-positive tumors were not reported (Table S5).

#### 3.2.2. Studies of Breast Cancer Prognosis

Characteristics of the 14 studies that examined the association between POPs and breast cancer prognosis are summarized in Table 3. 

Six studies examined mortality outcomes, with three measuring POPs in peripheral blood, one measuring POPs in breast adipose tissue and two measuring POPs in adipose tissue other than breast. Patients were aged between 58 and 66 years old in average with only two studies reporting the proportion of postmenopausal women (66% and 100% respectively), and two studies reporting proportion of invasive cancers (71% and 86% respectively). The proportion of ER-positive breast cancers varied between 72% and 78% in the three studies that have reported patients ER status (Tables S5 and S6).

Ten studies examined breast cancer prognostic factors, with three measuring POPs in peripheral blood, six measuring POPs in breast adipose tissue and one measuring POPs in adipose tissue other than breast. Patients were aged between 52 and 65 years old in average with varying proportions of premenopausal and postmenopausal women and none with at least 80% of postmenopausal patients. The proportion of invasive cancers varied between 85% and 100% and the proportion of ER-positive breast cancers varied between 50% and 86% with only two studies including at least 80% of ER-positive breast cancers (Table S8).

### 3.3. Risk of Bias in Retained Studies

Overall, studies reporting associations between peripheral blood POPs and breast cancer risk ranged from moderate to critical risk of bias, whereas studies reporting associations between adipose tissue POPs and breast cancer risk were more likely to be at serious or critical risk of bias.

Overall, studies reporting associations between POPs, measured in peripheral blood or in adipose tissue, and mortality outcomes were at serious risk of bias, whereas studies reporting associations with prognostic factors ranged from serious to critical risk of bias.

### 3.4. Systematic Data Synthesis

#### 3.4.1. Studies of Breast Cancer Risk

Among the 61 studies that examined associations between peripheral blood POPs and breast cancer risk, 30 reported a positive association with at least one POP, six reported a negative association with at least one POP, and 20 reported no association. Cohort-nested case-control studies with cumulative density (exclusive) sampling, population-based case-control studies not nested in a defined cohort and hospital-based case-control studies with both community- and hospital-controls were more likely to observe an association. Studies that included at least 80% of postmenopausal patients were more likely to observe an association whereas studies that included less than 80% of postmenopausal patients were more likely to observe no association. Studies that included at least 80% of invasive cancers and those with non-reported proportions of invasive breast cancers were more likely to observe an association. Studies with non-reported proportions of ER-positive breast cancers were more likely to observe an association whereas studies including less than 80% ER-positive breast cancers were slightly more likely to observe no association (Table S3).

Among the 26 studies that examined associations between breast adipose tissue POPs and breast cancer risk, 10 reported a positive association with at least one POP, two reported a negative association with at least one POP, and seven reported no association. Studies reporting the proportion of invasive breast cancers were slightly more likely to observe an association. Studies with non-reported proportions of ER-positive breast cancers were more likely to observe a positive association. The four studies of POPs in breast tumors did not report minimally adjusted estimates of risk (Table S4).

The two studies that examined associations between POPs in buttock adipose tissue and breast cancer risk reported negative associations (Table S5).

#### 3.4.2. Studies of Breast Cancer Prognosis

All three studies that examined peripheral blood POPs reported positive associations with both breast cancer all-cause and specific mortality, of which one study also reported a negative association with all-cause mortality (Table S6). The only study of breast adipose tissue POPs and breast cancer mortality reported a positive association with breast cancer recurrence. The two studies of POPs in adipose tissue other than breast reported negative associations with all-cause and breast cancer specific mortality respectively, of which one study reported a positive association with breast cancer specific mortality (Table S7).

Among the three studies that examined peripheral blood POPs and breast cancer prognostic factors, one study reported a positive association with tumor size and lymph-node involvement. Among the six studies of breast adipose tissue POPs, three studies examined associations with meaningful breast cancer prognostic factors but none of them reported minimally adjusted estimates. The only study of buttock adipose tissue POPs and breast cancer prognostic factors did not report minimally adjusted estimates (Table S8).

#### 3.4.3. Individual POPs and Breast Cancer Risk and Prognosis

One to 71 individual POPs were measured in studies of breast cancer risk (median = 9). Organochlorines were measured in 69 studies, of which 43 in blood, 21 in breast adipose tissue, two in adipose tissue other than breast and three in breast tumor. Polychlorinated biphenyls (PCBs) were measured in 57 studies, of which 38 were in blood, 16 in breast adipose tissue, one in adipose tissue other than breast and two in breast tumors. Dioxins were measured in four studies, of which two in blood and two in breast adipose tissue. Perfluoroalkyl substances (PFAS) were measured in three studies in blood. Bisphenol A (BPA) was measured in two studies in blood, polybrominated flame retardants (PBBs and PBDEs) in one study in blood and one study in breast adipose tissue whereas mono-ethyl phthalate (MEP) and parabens were measured in blood in one study respectively (Table S9). 

One to 35 individual POPs were measured in studies of breast cancer prognosis (median = 25). Organochlorines were measured in five studies of breast cancer mortality, of which three were in blood, one in breast adipose tissue and two in adipose tissue other than breast, whereas six studies measured organochlorines in relation to prognostic factors, of which three were in blood, four in breast adipose tissue and one in adipose tissue other than breast. PCBs were measured in four studies of breast cancer mortality, of which three were in blood, one in breast adipose tissue and one in adipose tissue other than breast, whereas four studies measured PCBs in relation to prognostic factors, of which two in blood, four in breast adipose tissue and one in adipose tissue other than breast (Tables S9 and S10). Parabens were measured in breast adipose tissue in one study in relation to prognostic factors.

The magnitude of the reported associations between POPs and breast cancer risk and mortality are summarized in Table 4.

When considering POPs positively associated with breast cancer risk in at least 10% of studies and at least two studies and no reported negative associations, eight individual POPs were consistently positively associated with breast cancer risk in blood: *p,p’*-Dichlorodiphenyldichloroethylene (*p,p’*-DDE), total or not specified DDE, β- Hexachlorocyclohexane (β-HCH), Dieldrin, PCB 118, PCB 138, PCB 170, PCB 180. Three individual POPs were consistently positively associated with breast cancer risk in breast adipose tissue: *p,p’*-DDE, total or not specified DDE, PCB 105. When considering POPs positively associated with breast cancer risk in at least one study and no reported negative associations, six individual POPs were positively associated with breast cancer risk in both blood and breast adipose tissue: *p,p’*-DDE, total or not specified DDE, Hexachlorobenzene (HCB), β-HCH, PCB 118 and PCB 180 (Tables S11 and S12).

When considering POPs positively associated with breast cancer mortality in at least 10% of studies and no reported negative associations, total PCBs and four individual POPs were positively associated with breast cancer mortality in blood: *p,p’*-Dichlorodiphenyltrichloroethane (*p,p’*-DDT), Dieldrin, PCB 174, PCB 177. Total PCBs and three individual POPs were positively associated with breast cancer mortality in breast adipose tissue: PCB 118, PCB 153, PCB 167 (Tables S13 and S14). Six individual POPs were positively associated with breast cancer prognostic factors in blood, in at least one study and with no reported negative associations: *p,p’*-DDE, Oxychlordane, *trans*-Nonachlor, β-HCH, PCB 138, PCB 153 (Table S16). 

Three individual POPs were positively associated with both breast cancer risk and prognosis either in blood or in breast adipose tissue: *p,p’*-DDE, β-HCH and PCB 118 (Tables S11–S15).

## 4. Discussion

The present systematic review of POPs and breast cancer indicates that studies of blood POPs and breast cancer risk accounted for much of the observed inconsistencies of epidemiological studies results. POPs measured in breast adipose tissue were more clearly associated with higher breast cancer risk. POPs were more consistently associated with worse breast cancer prognosis, whether measured in blood or breast adipose tissue, whereas POPs measured in adipose tissue other than breast were inversely associated with both breast cancer risk and prognosis. Some individual POPs measured in blood and breast adipose tissue were consistently associated with higher breast cancer risk and worse prognosis. However, the overall strength of evidence is weak, since few studies contributed to estimations of associations and the overall risk of bias in these studies ranged from moderate to critical. 

The inconsistencies between studies of blood POPs and breast cancer risk could be explained by methodological biases. In fact, more than half of these studies have measured POPs at the time of diagnosis which does not necessarily reflect the cumulative lifetime exposure to POPs and early-life exposures during critical windows of vulnerability [124]. Even though the majority of population-nested case-control studies and the only cohort study have measured POPs several months to many years before breast cancer occurrence, a point measurement of blood concentration of POPs is more likely to reflect recent dietary intakes and liver function [125,126] and can be affected by various events over time, such as weight loss or gain, pregnancies and breastfeeding [124,125,126]. The complex misclassification of POPs exposure resulting from blood measurements could have biased the observed associations toward the null, i.e., toward the observation of weaker associations or no associations at all. 

In this regard, adipose tissue, as a storage compartment for lipophilic POPs [127], is a more appropriate medium for estimating lifetime exposure to POPs. The observation of consistently positive associations with breast cancer risk in studies of breast adipose tissue POPs but consistently negative associations with POPs measured in adipose tissue other than breast is in line with the existent evidence of a protective function of adipose tissue in the wildlife and points toward the metabolic and toxicokinetic differences between different types of adipose tissue [127]. By accumulating POPs, adipose tissue away from breast decreases their availability to other tissues, thereby limiting their toxicity to the breast, whereas accumulation of POPs in breast adipose tissue exposes breast epithelial cells to their chronic local release. In fact, ultrastructural methods revealed regional differences in morphology of human subcutaneous tissues [128]. Abdominal adipose tissue, classified as deposit white adipose tissue, having large adipose cells and a poor collagenic component whereas adipocytes of breast adipose tissue, classified as structural white adipose tissue, are covered by a relatively dense connective capsule [128]. These regional differences in morphology explain the known regional differences in the metabolism of subcutaneous fatty depots that are related to their various functions. Thus, our results suggest that differences between adipose tissue subtypes may also have a toxicocokinetic impact on POPs.

Moreover, more than half of studies of blood POPs and breast cancer risk included more premenopausal than postmenopausal breast cancer patients. Although environmental exposures may be involved in premenopausal breast cancer occurrence, these cancers are primarily driven by a strong genetic susceptibility and are more often ER-negative cancers [129]. Furthermore, the increase in breast cancer incidence over the last decades reflects the increase in the incidence of postmenopausal breast cancers, which are more often ER-positive breast cancers [130], and thus more susceptible to the hormone-disrupting effects of POPs [8]. The selection bias created by inclusion of large proportions of premenopausal breast cancers, which are less likely to be related to POPs exposure, could have biased the observed associations toward the null. In fact, we observed that studies that included less than 80% of postmenopausal patients and those including fewer than 80% ER-positive breast cancers were more likely to observe no association. 

Another important issue was related to statistical methods used for selecting potential confounders. If the majority of studies of blood POPs and breast cancer risk have considered important confounders in their statistical models, methods used for selecting potential confounders were not always appropriate. In particular, the majority of case-control studies used the change in estimate method, which is not appropriate for accurate estimations of associations. Changes in estimates may be observed when adjusting for colliders (i.e., non-confounders that introduce a selection bias) and when non-collapsible effect measures such as odds ratios are used [131]. The bias introduced by this method can be difficult to predict when numerous variables are tested for confounding and can lead to discrepant studies results. 

The strengths of the present systematic review include the use of the Cochrane Reviews rigorous methodology, the extensive and highly sensitive search strategy to retrieve as many relevant studies as possible, the use of a pre-established protocol, the assessment of the risk of bias, and the systematic analysis of results, in addition to considering sources of heterogeneity between studies results. Limitations include the lack of high-quality evidence inherent in observational study designs and the overall critical risk of bias in included studies. 

Finally, although the present systematic review has identified some individual POPs associated with both breast cancer risk and prognosis that deserve further investigation, it should be emphasized that different POPs have different metabolic profiles and can have synergistic or antagonistic effects, and that proportions of different POPs may vary from one person to another. Thus, approaches considering the simultaneous exposure to different POPs may be more relevant than the isolated analysis of individual POPs. 

## 5. Conclusions

Over the past three decades, numerous epidemiological studies have attempted to assess the association between exposure to POPs and breast cancer. Despite the apparent inconsistencies between studies, which were mainly due to methodological biases and to differences in the biological sample used for exposure measurement, when considering all studies (peripheral blood and adipose tissue) and all outcomes together (risk and prognosis), there was a trend toward a positive association between exposure to POPs and breast cancer that deserves further investigation. Future studies need to use rigorous methodology by including the relevant study population, using an appropriate biological sample for POPs measurement, controlling properly for confounding and assessing combined effects of POPs.

## Figures and Tables

**Table 1:** Summary characteristics of studies of peripheral blood POPs and breast cancer risk (*n* = 61).

**Design**	Cohort studies: *n* = 1 Case-cohort studies: *n* = 1 Cohort nested case-control studies with incidence density (risk-set) sampling: *n* = 13 Cohort nested case-control studies with cumulative density (exclusive) sampling: *n* = 3 Population-based case-control studies not nested in a defined cohort: *n* = 9 Hospital-based case-control studies with community controls: *n* = 8 Hospital-based case-control studies with both community-controls and hospital-controls: *n* = 4 Hospital-based case-control studies with hospital-controls: *n* = 19 Case-control studies (unclassified): *n* = 1 Cross-sectional studies: *n* = 2	
**Breast cancer patients**	Number of breast cancer patients: 20 to 902 Mean age: 40 to 66 years; NR in 13 studies. Postmenopausal patients: 0% to 100%, median = 61%; two studies exclusively in premenopausal patients; NR in 19 studies. Invasive breast cancers: 62% to 100%, median = 99.5%; NR in 35 studies Estrogen receptor positive breast cancers: 32% to 87%, median 64%; NR in 40 studies	
**POPs**	*Measurement method:* GC-ECD: *n* = 43 HPLC-MS-MS: *n* = 5 GC-MS: *n* = 4 GC-ID-HRMS: *n* = 3 HPLC/FD: *n* = 2 LC-MS-MS: *n* = 1 GC-IDMS: *n* = 1 GC: *n* = 3 MS: *n* = 1 NR: *n* = 2	*Lipid adjustment: n* = 39; NR in 18 studies *Measured POPs:* PCBs: *n* = 38 Organochlorines: *n* = 48 Dioxins: *n* = 2 PFAS: *n* = 3 Phthalates: *n* = 1 Parabens: *n* = 1 BPA: *n* = 2 PBBs: *n* = 1

*n*: number of studies; NR: not reported; POPs: persistent organic pollutants; MS: mass spectrometry; GC-ECD: gas chromatography with electron Capture Detector; HLPC-MS-MS: high-performance liquid chromatography-tandem mass spectrometry; HPLC/FD: high-performance liquid chromatography with fluorescence detection; LC-MS-MS: liquid chromatography–tandem mass spectrometry; GC-MS-MS: gas chromatography-tandem mass spectrometry; GC-IDMS: gas-chromatography isotope-dilution mass-spectrometry; GC-ID-HRMS: gas chromatography-isotope dilution high-resolution mass spectrometry; HR-GC-ECD: high-resolution gas chromatography with micro-electron capture detection; GC: gas chromatography; PCBs:. polychlorinated biphenyls; PFAS: perfluoroalkyl substances; BPA: bisphenol A; PBBs: polybrominated biphenyls.

**Table 2:** Summary characteristics of studies of breast adipose tissue POPs and breast cancer risk (*n* = 26).

**Design**	Hospital-based case-control studies with hospital-controls: *n* = 26	
**Breast cancer patients**	Number of breast cancer patients: 5 to 304 Mean age: 40 to 63 years; NR in three studies. Postmenopausal patients: 49% to 82%, median = 56%; NR in 16 studies. Invasive breast cancers: 76% to 100%, median = 100%; NR in 11 studies Estrogen receptor positive breast cancers: 45% to 79%, median 64%; NR in 15 studies	
**POPs**	*Measurement method:* GC-ECD: *n* = 14 GC-MS: *n* = 9 GC: *n* = 4 *Lipid adjustment: n* = 23; NR in three studies	*Measured POPs:* PCBs: *n* = 16 Organochlorines: *n* = 21 Dioxins: *n* = 2 PBDEs: *n* = 1

*n*: number of studies; NR: not reported; POPs: persistent organic pollutants; GC-ECD: gas chromatography with electron Capture Detector; GC-MS: gas-chromatography mass-spectrometry; GC: gas chromatography; PCBs: polychlorinated biphenyls; PFAS: perfluoroalkyl substances; PBDE: polybrominated diphenyl ethers.

**Table 3:** Summary characteristics of studies of POPs and breast cancer prognosis (*n* = 14).

**Design**	Studies of mortality: *n* = 6 Cohort studies *n* = 5 Ecologic study: *n* = 1 Studies of prognostic factors: *n* = 10 Cross-sectional studies: *n* = 10	
**Breast cancer patients**	Studies of mortality: *n* = 6 Number of breast cancer patients: 161 to 633 Mean age: 58 to 66 years; NR in one study. Postmenopausal patients: 66% to 100% (in two studies); NR in three studies. Invasive breast cancers: 71% to 86% (in two studies); NR in three studies Estrogen receptor positive breast cancers: 72% to 78% (in three studies); NR in three studies Studies of prognostic factors: *n* = 10 Number of breast cancer patients: 40 to 409 Mean age: 52 to 65 years Postmenopausal patients: 41 % to 100%, median = 63%; NR in four studies. Invasive breast cancers: 85% to 100%, median = 100%; NR in three studies Estrogen receptor positive breast cancers: 50% to 86%, median 68%	
**POPs**	Studies of mortality: *n* = 6 *Type of sample:* Peripheral blood: *n* = 3 Breast adipose tissue: *n* =1 Adipose tissue other than breast: *n* = 2 *Measurement method:* GC-ECD: *n* = 4 GC-MS: *n* = 1 NR: *n* = 1 *Lipid adjustment: n* = 5; NR in 1 study *Measured POPs:* PCBs: *n* = 4 Organochlorines: *n* = 5	Studies of prognostic factors: *n* = 10 *Type of sample:* Peripheral blood: *n* = 3 Breast adipose tissue: *n* = 6 Adipose tissue other than breast: *n* = 1 *Measurement method:* GC-ECD: *n* = 6 GC-MS: *n* = 3 HLPC-MS-MS: *n* = 1 *Lipid adjustment: n* = 9; NR in 1 study *Measured POPs:* PCBs: *n* = 4 Organochlorines: *n* = 6

*n*: number of studies; NR: not reported; POPs: persistent organic pollutants; GC-MS: gas-chromatography mass-spectrometry; GC-ECD: gas chromatography with electron Capture Detector; HLPC-MS-MS: high-performance liquid chromatography-tandem mass spectrometric; PCBs: polychlorinated biphenyls.

**Table 4:** Main results summary of studies reporting positive ^*^ associations between POPs and breast cancer risk and mortality.

Type of Tissue Sample	Studies with Positive * Associations/Total Studies	Range of Associations ** Estimate [95% CI]
**Breast cancer risk**		
Blood	29/61	From OR = 1.1 [1.0–1.2] (*Heptachlor, continuous*) to OR = 7.6 [1.1–51.4] (*PCBs group 1a, variable form NR*)
Breast adipose tissue	10/26	From OR = 1.1 [1.0–1.3] (*PCB 180, quart 4 vs. quart 1*) to OR = 10.5 [2.0–55.3] (*β-HCH, >0.1 mg/kg vs. <0.1 mg/kg*)
Adipose tissue other than breast	0/2	NA
**Breast cancer mortality**		
Blood	3/3	From HR = 1.9 [1.1–3.4] (*15-year breast cancer mortality PCB 174, tert 3 vs. tert 1*) to HR = 5.8 [1.6–20.5] (*breast cancer recurrence and/or death, Dieldrin, quart 4 vs. quart 1*)
Breast adipose tissue	1/1	From HR = 2.6 [1.0–7.1] (*Breast cancer recurrence, PCB 153, tert 3 vs. tert1*) to HR = 4.0 [1.3–4.9] (*PCB 118, Breast cancer recurrence, tert 3 vs. tert1*)
Adipose tissue other than breast	1/2	MRR = 1.3 [1.1–1.5] (*Breast-cancer specific mortality, Dieldrin, linear estimates per inter-quartile range*)

POPs: persistent organic pollutants; CI: Confidence interval; NA: Not applicable; NR: not reported; * positive association: an observed higher risk or mortality with higher POPs exposure; ** Adjusted for all important confounders; OR: odds ratio; MRR: mortality rate ratio; PCB: Polychlorinated biphenyls; HR: hazard ratio, β-HCH: β-Hexachlorocyclohexane.

## Supplementary Materials

The following are available online at https://www.mdpi.com/2072-6694/11/8/1063/s1, Table S1: Search strategy for Medline via PubMed, Table S2: Studies not included in the qualitative synthesis, Table S3: Studies of POPs measured in peripheral blood and breast cancer risk, Table S4: Studies of POPs measured in breast adipose tissue and breast cancer risk, Table S5: Studies of POPs measured in adipose tissue other than breast and breast cancer risk, Table S6: Studies of POPs measured in peripheral blood and mortality among breast cancer patients, Table S7: Studies of POPs measured in adipose tissue and mortality among breast cancer patients, Table S8: Studies of POPs and breast cancer prognostic factors, Table S9: Results of studies of POPs and breast cancer risk, Table S10: Results of studies of POPs and mortality in breast cancer patients, Table S11: Results of studies of POPs and prognostic factors in breast cancer patients, Table S12: Summary results of POPs associated positively with breast cancer risk, Table S13: Summary results of POPs associated negatively with breast cancer risk, Table S14: Summary results of POPs associated positively with mortality among breast cancer patient, Table S15: Summary results of POPs associated negatively with mortality among breast cancer patients, Table S16: Summary results of POPs associated positively with breast cancer prognostic factors.
