# Supplementary material for: Persistent Organic Pollutants and Breast Cancer: A Systematic Review and Critical Appraisal of the Literature
Source: Cancers (Basel). 2019 Jul 27;11(8):1063. doi: 10.3390/cancers11081063 (PMC6721417; doi:10.3390/cancers11081063)
Supplement: Supplementary file 1 [file cancers-11-01063-s001.pdf]

Table S1. Search strategy for Medline via PubMed

Table S2. Studies not included in the qualitative synthesis

Table S3. Studies of POPs measured in peripheral blood and breast cancer risk

Table S4. Studies of POPs measured in breast adipose tissue and breast cancer risk

Table S5. Studies of POPs measured in adipose tissue other than breast and breast cancer risk

Table S6. Studies of POPs measured in peripheral blood and mortality among breast cancer patients

Table S7. Studies of POPs measured in adipose tissue and mortality among breast cancer patients

Table S8. Studies of POPs and breast cancer prognostic factors

Table S9. Results of studies of POPs and breast cancer risk

Table S10. Results of studies of POPs and mortality in breast cancer patients

Table S11. Results of studies of POPs and prognostic factors in breast cancer patients

Table S12. Summary results of POPs associated positively with breast cancer risk

Table S13. Summary results of POPs associated negatively with breast cancer risk

Table S14. Summary results of POPs associated positively with mortality among breast cancer patient

Table S15. Summary results of POPs associated negatively with mortality among breast cancer patients

Table S16. Summary results of POPs associated positively with breast cancer prognostic factors

Table S1. Search strategy for Medline via PubMed

(("Breast" [tiab] OR "mammary gland"[tiab] OR mammary[tiab]) AND (cancer[tiab] OR carcinoma[tiab]OR carcinomas[tiab] OR "malignant neoplasm"[tiab] OR "malignant neoplasms"[tiab] OR "malignant tumor"[tiab] OR "malignant tumors"[tiab] OR "malignant tumour"[tiab] OR "malignant tumours"[tiab])) OR "Breast Neoplasms"[Mesh]
AND<br>"Endocrine disrupting chemicals"[tiab] OR "Endocrine disrupting chemical"[tiab] OR "Endocrine Disruptors"[tiab] OR "Endocrine Disruptor"[tiab] OR "Endocrine Disruptor Effect"[tiab] OR "EDCs"[tiab] OR "Body Burden"[tiab] OR "Body Burdens"[tiab] OR "Environmental pollutant"[tiab] OR "Environmental Carcinogens"[tiab] OR "Environmental Pollution"[tiab] OR "Environmental pollutants"[tiab] OR "persistent organic pollutants"[tiab] OR "persistent organic pollutant"[tiab] OR "POP"[tiab] OR "POPs"[tiab] OR "Organic solvents"[tiab] OR "Organic solvent"[tiab] OR "Pesticide"[tiab] OR "Pesticides"[tiab] OR "Polycyclic aromatic hydrocarbon"[tiab] OR "Polycyclic aromatic hydrocarbons"[tiab] OR "Aromatic Polycyclic Hydrocarbons"[tiab] OR "Aromatic Polycyclic Hydrocarbon"[tiab] OR "PAH"[tiab] OR "Plasticizers"[tiab] OR "Plasticizer"[tiab] OR "Fertilizers"[tiab] OR "Fertilizer"[tiab] OR "Herbicide"[tiab] OR "Herbicides"[tiab] OR "Insecticide"[tiab] OR "Insecticides"[tiab] OR "Insect Repellents"[tiab] OR "Insect Repellent"[tiab] OR "Coloring Agents"[tiab] OR "Coloring Agent"[tiab] OR "Dyes"[tiab] OR "Dye"[tiab] OR "Pigments"[tiab] OR "Pigment"[tiab] OR "Stains"[tiab] OR "Hair Dyes"[tiab] OR "Hair Dye"[tiab] OR "Hair Coloring Agents"[tiab] OR "Hair Colorants"[tiab] OR "Cosmetics"[tiab] OR "Cosmetic"[tiab] OR "Food Coloring Agents"[tiab] OR "Food Coloring Agent"[tiab] OR "Food Colorants"[tiab] OR "Food Colorant"[tiab] OR "Food Additives"[tiab] OR "Food Additive"[tiab] OR "Flavoring Agents"[tiab] OR "Flavoring Agent"[tiab] OR "Flavor Additives"[tiab] OR "Flavor Additive"[tiab] OR "Flavor Enhancers"[tiab] OR "Flavor Enhancer"[tiab] OR "Sweetening Agents"[tiab] OR "Sweetening Agent"[tiab] OR "Artificial Sweeteners"[tiab] OR "Artificial Sweetener"[tiab] OR "Sugar Substitute"[tiab] OR "Sugar Substitutes"[tiab] OR "Sweeteners"[tiab] OR "Food Preservatives"[tiab] OR "Food Preservative"[tiab] OR "Organochlorine Compounds"[tiab] OR "Organochlorine Compound"[tiab] OR "Chlorinated Hydrocarbons"[tiab] OR "Chlorinated Hydrocarbon"[tiab] OR "Organic Chlorine Compounds"[tiab] OR "Organic Chlorine Compound"[tiab] OR "Organochlorines"[tiab] OR "Organochlorine"[tiab] OR "Aldrin"[tiab] OR "Isodrin"[tiab] OR "Dieldrin"[tiab] OR "Alvit-55"[tiab] OR "Alvit 55"[tiab] OR "Alvit55"[tiab] OR "Endrin"[tiab] OR "Hexadrin"[tiab] OR "Chlordan"[tiab] OR "Chlordane"[tiab] OR "Chlorobenzenes"[tiab] OR "Chlorobenzene"[tiab] OR "Chlorophenols"[tiab] OR "Chlorophenol"[tiab] OR "Hydroxychlorobenzenes"[tiab] OR "Hydroxychlorobenzene"[tiab] OR "Hexachlorobenzene"[tiab] OR "HCB"[tiab] OR "DDT"[tiab] OR "Dichlorodiphenyltrichloroethane"[tiab] OR "TbisC-ethane"[tiab] OR "TbisC ethane"[tiab] OR "Chlorophenothane"[tiab] OR "Benzochloryl"[tiab] OR "Dichlorodiphenyl Dichloroethylene"[tiab] OR "DDMU"[tiab] OR "DDE"[tiab] OR "Dichlorodiphenyldichloroethylene"[tiab] OR "DDX"[tiab] OR "Dichlorodiphenyldichloroethane"[tiab] OR "TDE"[tiab] OR "DDD"[tiab] OR "Heptachlor"[tiab] OR "Heptachlor Epoxide"[tiab] OR "Lindane"[tiab] OR "gamma-Benzene Hexachloride"[tiab] OR "Hexachloride gamma-Benzene"[tiab] OR "gamma Benzene Hexachloride"[tiab] OR "Hexachlorane"[tiab] OR "Hexachlorocyclohexane"[tiab] OR "Benzene Hexachloride"[tiab] OR "gamma-Hexachlorocyclohexane"[tiab] OR "gamma Hexachlorocyclohexane"[tiab] OR "BHC Insecticide"[tiab] OR "Delitex"[tiab] OR "Gammexane"[tiab] OR "Tetocid"[tiab] OR "Kwell"[tiab] OR "PMS-Lindane"[tiab] OR "PMS Lindane"[tiab] OR "PMSLindane"[tiab] OR "Scabecid"[tiab] OR "Scabene"[tiab] OR "Scabisan"[tiab] OR "Gamma-666"[tiab] OR "Gamma 666"[tiab] OR "Gamma666"[tiab] OR "Jacutin"[tiab] OR "Methoxychlor"[tiab] OR "DMDT"[tiab] OR "Dianisyl Trichloroethane"[tiab] OR "Trichloroethane Dianisyl"[tiab] OR "Metox"[tiab] OR "Methylene Chloride"[tiab] OR "Chloride Methylene"[tiab] OR "Methylene Bichloride"[tiab] OR "Bichloride Methylene"[tiab] OR "Dichloride Methylene"[tiab] OR "Dichloromethane"[tiab] OR "Solaesthin"[tiab] OR "Mirex"[tiab] OR "Toxaphene"[tiab] OR "Polychlorocamphene"[tiab] OR "Vinyl Chloride"[tiab] OR "Chloroethylene"[tiab] OR "nonachlor"[tiab] OR "nonachlor (trans)-isomer"[tiab] OR "nonachlor (cis)-isomer"[tiab] OR "oxychlordane" [tiab] OR "Polychlorinated Biphenyls"[tiab] OR "Biphenyls Polychlorinated"[tiab] OR "Polychlorobiphenyl Compounds"[tiab] OR "Compounds Polychlorobiphenyl"[tiab] OR "PCBs"[tiab] OR "PCB"[tiab] OR "trichlorobiphenyl"[tiab] OR "TCB"[tiab] OR "tetrachlorobiphenyl"[tiab] OR "pentachlorobiphenyl"[tiab] OR "hexachlorobiphenyl"[tiab] OR "CB-153"[tiab] OR "heptachlorobiphenyl"[tiab] OR "CB187 cpd"[tiab] OR "Octachlorobiphenyl"[tiab] OR "Nonachlorobiphenyl"[tiab] OR "aroclor 1260"[tiab] OR "aroclor-1260"[tiab] OR "aroclor1260"[tiab] OR "Askarel"[tiab] OR "Phenoxy Herbicide"[tiab] OR "Dichlorophenoxyacetic Acid"[tiab] OR "Monosan"[tiab] OR "DCP"[tiab] OR "Dichlorophenol"[tiab] OR "Per- and polyfluoroalkyl substances"[tiab] OR "PFASs"[tiab] OR "fluorinated chemicals"[tiab] OR "Polybrominated Flame Retardants"[tiab] OR "Halogenated Diphenyl Ethers"[tiab] OR "Chlorinated Diphenyl Ethers"[tiab] OR "PCDEs"[tiab] OR "PCDE Compounds"[tiab] OR "Iodinated Diphenyl Ethers"[tiab] OR "Brominated Diphenyl Ethers"[tiab] OR "PBDEs"[tiab] OR "Polybrominated Diphenyl Ethers"[tiab] OR "PBDE Compounds"[tiab] OR "Fluorinated Diphenyl Ethers"[tiab] OR "hexabromobiphenyl"[tiab] OR "HBB"[tiab] OR "PBB 153"[tiab] OR "PBB153"[tiab] OR "PBB-153"[tiab] OR "Dibromodiphenyl Ether"[tiab] OR "PBDE 15"[tiab] OR "PBDE15"[tiab] OR "PBDE-15"[tiab] OR "tribromodiphenyl ether 28"[tiab] OR "BDE-28"[tiab] OR "tribrominated diphenyl ether"[tiab] OR "tribromodiphenyl ether"[tiab] OR "PBDE 17"[tiab] OR "PBDE17"[tiab] OR "PBDE-17"[tiab] OR "PBDE 25"[tiab] OR "PBDE25"[tiab] OR "PBDE-25"[tiab] OR "PBDE 28"[tiab] OR "PBDE28"[tiab] OR "PBDE-28"[tiab] OR "PBDE 33"[tiab] OR "PBDE33"[tiab] OR "PBDE-33"[tiab] OR "tetrabromodiphenyl ether"[tiab] OR "PBDE-47"[tiab] OR "PBDE47"[tiab] OR "PBDE 47"[tiab] OR "TBDP-ether"[tiab] OR "brominated diphenyl ether"[tiab] OR "BDE-47"[tiab] OR "BDE47"[tiab] OR "BDE 47"[tiab] OR "pentabromodiphenyl ether"[tiab] OR "PBDE"[tiab] OR "PBDE 100"[tiab] OR "PBDE-100"[tiab] OR "PBDE100"[tiab] OR "PBDE 99"[tiab] OR "PBDE99"[tiab] OR "PBDE- 99"[tiab] OR "pentaBDE"[tiab] OR "PBDE 85"[tiab] OR "PBDE85"[tiab] OR "PBDE-85"[tiab] OR "DE 71"[tiab] OR "DE-71"[tiab] OR "DE71"[tiab] OR "Hexabromodiphenyl Ether"[tiab] OR "PBDE 153"[tiab] OR "PBDE153"[tiab] OR "PBDE-153"[tiab] OR "Environmental Phenol"[tiab] OR "bisphenol A"[tiab] OR "bisphenolA"[tiab] OR "bisphenol-A"[tiab] OR "Diphenylolpropane"[tiab] OR "BPA"[tiab] OR "Organophosphate Insecticides"[tiab] OR "Cholinesterase Reactivators"[tiab] OR "Organophosphorus Compounds"[tiab] OR "dimethyl phosphate" [tiab] OR "dimethylphosphate" [tiab] OR "dimethyl-phosphate" [tiab] OR "DMP" [tiab] OR "Organothiophosphates"[tiab] OR "Organothiophosphate"[tiab] OR "Organo-thiophosphate"[tiab] OR "dimethyl phosphorothionate"[tiab] OR "DMTP cpd" [tiab] OR "DMTP" [tiab] OR "dimethylthiophosphate" [tiab] OR "dimethyl phosphorothionate" [tiab] OR "dimethyl-phosphorothionate" [tiab] OR "dimethyl phosphorodithioate"[tiab] OR "DMDTP"[tiab] OR "dimethyldithiophosphate"[tiab] OR "Diethylphosphate"[tiab] OR "DEP"[tiab] OR "diethyl phosphorothionate" [tiab] OR "Diethylthiophosphate"[tiab] OR "DETP"[tiab] OR "dimethyl phosphorodithioate"[tiab] OR "Dimethyldithiophosphate"[tiab] OR "DMDTP"[tiab] OR "DEDTP"[tiab] OR "Fluorocarbons"[tiab] OR "Perfluorinated Compounds"[tiab] OR "Perfluorocarbons"[tiab] OR "Fluorinated Compounds"[tiab] OR "Polyfluorinated Compounds"[tiab] OR "perfluorooctane sulfonic acid"[tiab] OR "Perfluorooctane Sulfonate"[tiab] OR "heptadecafluoro-1-octane sulfonic acid"[tiab] OR "heptadecafluorooctane sulfonic acid"[tiab] OR "perfluorooctanesulfonic acid"[tiab] OR "PFOSA"[tiab] OR "PFOS"[tiab] OR "perfluorooctanyl sulfonate"[tiab] OR "PFOS compound"[tiab] OR "Perfluorooctanesulfonate"[tiab] OR "lithium perfluorooctane sulfonate"[tiab] OR "perfluorooctane sulfonate"[tiab] OR "perfluorooctanoic acid"[tiab] OR "PFOA"[tiab] OR "pentadecafluorooctanoic acid"[tiab] OR "perfluorinated octanoic acid"[tiab] OR "perfluorooctanoyl chloride"[tiab] OR "PFOA cpd"[tiab] OR "sodium perfluorooctanoate"[tiab] OR "perfluorooctanoate"[tiab] OR "ammonium perfluorooctanoate"[tiab] OR "APFO"[tiab] OR "Perfluorohexane Sulfonate"[tiab] OR "Sulfonate Perfluorohexane "[tiab] OR "PFHxS"[tiab] OR "DBCA"[tiab] OR "DCCA"[tiab] OR "CDCCA compound"[tiab] OR "TDCCA compound"[tiab] OR "PBA"[tiab] OR "Phthalates"[tiab] OR "Phthalate"[tiab] OR "Phthalic Acid"[tiab] OR "Phthalic Acids"[tiab] OR "mono-benzyl phthalate"[tiab] OR "phthalic acid monobenzyl ester" [tiab] OR "MBzP"[tiab] OR "Dibutyl Phthalate"[tiab] OR "Di-n-Butyl Phthalate"[tiab] OR "Di n Butyl Phthalate"[tiab] OR "Phthalate Di-n-Butyl"[tiab] OR "Butyl Phthalate"[tiab] OR "Phthalate Butyl"[tiab] OR "Diethylhexyl Phthalate"[tiab] OR "Dioctyl Phthalate"[tiab] OR "Phthalate Dioctyl"[tiab] OR "Di-2-Ethylhexylphthalate"[tiab] OR "Di 2 Ethylhexylphthalate"[tiab] OR "DEHP"[tiab] OR "monobutyl phthalate"[tiab] OR "mono-n-butyl phthalate"[tiab] OR "MnBP"[tiab] OR "Mono-ethyl Phthalate"[tiab] OR "MEP"[tiab] OR "MEHHP cpd"[tiab] OR "Mono-cyclohexyl Phthalate"[tiab] OR "MCHP"[tiab] OR "monoisononylphthalate"[tiab] OR "Mono-isononyl Phthalate"[tiab] OR "MiNP"[tiab] OR "monomethyl phthalate"[tiab] OR "mono-methyl phthalate"[tiab] OR "MMP"[tiab] OR "mono-n-octyl phthalate" [tiab] OR "MNOP"[tiab] OR "MOP"[tiab] OR "MCPP "[tiab] OR "Mono-3-carboxypropyl Phthalate"[tiab] OR "Mono-2-ethylhexyl Phthalate"[tiab] OR "MEHP"[tiab] OR "MEHP cpd"[tiab] OR "MEOHP cpd"[tiab] OR "MEOHP"[tiab] OR "MEHHP cpd"[tiab] OR "MEHHP"[tiab] OR "Diethylhexyl Phthalate"[tiab] OR "Dioctyl Phthalate"[tiab] OR "Di-2-Ethylhexylphthalate"[tiab] OR "Di 2 Ethylhexylphthalate"[tiab] OR "DEHP"[tiab] OR "Acronine"[tiab] OR "Acronycin"[tiab] OR "Acronycine"[tiab] OR "dichloroethane"[tiab] OR "Methylene Chloride"[tiab] OR "Chloride Methylene"[tiab] OR "Methylene Bichloride"[tiab] OR "Bichloride Methylene"[tiab] OR "Methylene Dichloride"[tiab] OR "Dichloride Methylene"[tiab] OR "Dichloromethane"[tiab] OR "Solaesthin"[tiab] OR "Benzene"[tiab] OR "Benzole"[tiab] OR "Cyclohexatriene"[tiab] OR "Benzol"[tiab] OR "ethylene dichloride"[tiab] OR "eugenol methyl ether"[tiab] OR "dimethoxybenzene"[tiab] OR "methyl eugenol"[tiab] OR "dimethoxybenzene"[tiab] OR "BBMPD"[tiab] OR "propylene dichloride"[tiab] OR "dichloropropane"[tiab] OR "nithiazide"[tiab] OR "Nithiazid"[tiab] OR "Hepzide"[tiab] OR "butadiene"[tiab] OR "divinyl"[tiab] OR "butadiene"[tiab] OR "Dichlorvos"[tiab] OR "Dichlofos"[tiab] OR "Dimethyl Dichlorovinyl Phosphate"[tiab] OR "DDVP"[tiab] OR "Dichlorophos"[tiab] OR "Divipan"[tiab] OR "Novotox"[tiab] OR "nitroacenaphthene"[tiab] OR "Environmental Pollutants"[Majr:NoExp] OR "Carcinogens, Environmental"[Majr:NoExp] OR "Pesticides"[Majr:NoExp] OR "Pesticide Residues"[Majr:NoExp] OR "Solvents"[Majr:NoExp] OR "Polycyclic Aromatic Hydrocarbons"[Majr:NoExp] OR "Plasticizers"[Majr:NoExp] OR "Fertilizers"[Majr:NoExp] OR "Endocrine Disruptors"[Majr:NoExp] OR "Herbicides"[Majr:NoExp] OR "Insecticides"[Majr:NoExp] OR "Body Burden"[Majr:NoExp] OR "Insect Repellents"[Majr:NoExp] OR "Coloring Agents"[Majr:NoExp] OR "Food Additives"[Majr:NoExp] OR "Food Coloring Agents"[Majr:NoExp] OR "Flavoring Agents"[Majr:NoExp] OR "Sweetening Agents"[Majr:NoExp] OR "Food Preservatives"[Majr:NoExp] OR "Hair Dyes"[Majr:NoExp] OR "Cosmetics"[Majr:NoExp]
AND<br>"Risk"[tiab] OR "Risks"[tiab] OR "Incidence"[tiab] OR "Incidences"[tiab] OR "occurrence"[tiab] OR "occurrences"[tiab] OR "prevalence"[tiab] OR "prevalences"[tiab] OR "Prognosis"[tiab] OR "Prognoses"[tiab] OR "Prognostic"[tiab] OR "severity"[tiab] OR "severities"[tiab] OR "stage"[tiab] OR "stages"[tiab] OR "Survival"[tiab] OR "Survivals"[tiab] OR "Survivor"[tiab] OR "Survivors"[tiab] OR "mortality"[tiab] OR "mortalities"[tiab] OR "Fatality"[tiab] OR "Death"[tiab] OR "Fatal Outcome"[tiab] OR "Fatal Outcomes"[tiab] OR "Outcome"[tiab] OR "Outcomes"[tiab] OR "Progression"[tiab] OR "Progressions"[tiab] OR "Recurrence"[tiab] OR "Recurrences"[tiab] OR "Relapse"[tiab] OR "Relapses"[tiab] OR "Risk"[Mesh] OR "Incidence"[Mesh] OR "Prognosis"[Mesh] OR "Survival"[Mesh] OR "Mortality"[Mesh] OR "Fatal Outcome"[Mesh] OR "Disease-Free Survival"[Mesh] OR "Recurrence"[Mesh]
NOT (animals [mh] NOT humans [mh])

Table S2. Studies not included in the qualitative synthesis

Reference	Participants (n)	Result	Reason of non-inclusion
Orhan H 2017	NR	"Tissue and blood POP concentrations were, although weakly, associated with cancers in patients."	Insufficient information on study design, population, analyses and results

Table S2. Studies of POPs measured in peripheral blood and breast cancer risk

Study reference<br>Publication year<br>Location	Design	Patients and tumors characteristics*<br>(mean ± SD)	Exposure assessment	Evaluated POPs	Associated POPs<br>Estimate [95% confidence interval]<br>Without adjustment	 <br>With adjustment**	Comments
**Cohort studies**							
Warner M et al.<br>2011 [1]<br>Pesatori AC et al.<br>2009 [2]<br>Warner M et al.<br>2002 [3]<br>Italy	. Prospective<br>. Lag time: not considered<br>. n=833<br>. n=66 incident cases<br>. Follow-up: 32 y	. Age: 48.8 ± 11.3 y<br>. Post-menopausal: 51%<br>. Invasiveness: NR<br>. % Invasive: NR<br>. % ER+: 87%	. Timing: not considered<br>. Serum<br>. Method: MS<br>. Lipid-adjusted	. Dioxin (TCDD)	HR=1.49 [0.93, 2.38]	HR=1.44 [0.89, 2.33]	. n=96 non-detectable values imputed half the detection limit<br>. Model selection: change in estimate (10%) ; method: NR<br>. HR for 10-fold increase of TCDD;<br>. Residence around a chemical explosion site
**Case-cohort studies**							
Bonefeld-Jørgensen EC et al.<br>2014 [4]<br>Denmark	. Prospective<br>. Lag time: not considered<br>. n=250 cases<br>. n=233 controls<br>. Follow-up: 10-15 y	. Age: mean=40.8 y, range [31–53]<br>. Pre-menopausal<br>. Invasive and non-invasive cancer<br>. % Invasive: NR<br>. % ER+: NR	. Timing: pregnancy<br>. Serum<br>. Method: LC-MS-MS<br>. Lipid-adjustment : NR	PFAS:<br>. 10 PFCAs<br>. 5 PFSAs<br>. PFOSA<br>*(individually and summed)*	. PFHxS, continuous :<br>RR=0.66 [NR]<br><br>. PFOSA, continuous:<br>RR=1.03 [NR]	. PFHxS, continuous :<br>RR=0.66 [0.47–0.94]<br><br>. PFOSA, quint 5 *vs* quint 1:<br>RR=1.89 [1.01–3.54]	. Non-detectable values imputed half the detection limit<br>. Model selection: none<br>(*a priori* variable selection)
**Cohort-nested case-control studies with incidence density (risk-set) sampling**							
Hurley S et al.<br>2018 [5]<br>USA (CTS)	. Retrospective<br>. Lag time: not considered<br>. n=902 cases<br>. n=858 controls<br>. Follow-up: NA	. Age: 96% ≥ 50 y<br>. Post-menopausal: 93%<br>. Invasive cancer<br>. % Invasive: 100%<br>. % ER+: 82%	. Timing: after diagnosis<br>. Serum<br>. Method: HPLC-MS-MS<br>. Lipid-adjustment: none	PFAS with detection frequencies ≥ 95%<br>. PFOA<br>. PFNA<br>. PFUnDA<br>. PFHxS<br>. PFOS<br>. MeFOSAA	. PFOA: OR=0.7 [0.5-1.0]<br>. PFNA: OR=1.0 [0.7-1.4]<br>. PFUnDA: OR=0.9 [0.7-1.1]<br>. PFHxS: OR=0.8 [0.6-1.0]<br>. PFOS: OR=1.0 [0.8-1.4]<br>. MeFOSAA: OR=0.9 [0.7-1.1]	. PFOA: 0.7 [0.5-1.1]<br>. PFNA: OR=0.9 [0.6-1.2]<br>. PFUnDA: OR=0.9 [0.7-1.1]<br>. PFHxS: OR=0.8 [0.6-1.1]<br>. PFOS: OR=0.9 [0.7-1.3]<br>. MeFOSAA: OR=1.0 [0.8-1.2]<br><br>*(log_{10} [PFAS] , ng/mL])*	. Non-detectable values: imputed the detection limit value divided by the square root of 2<br>. Model selection: descending retaining variables with a p-value < 0.05 then ascending with a change of estimate criterion (10%)
Cohn BA et al.<br>2015 [6]<br>USA (CHDS daughters)	. Prospective<br>. Lag time: not considered<br>. n=103 cases<br>. n=315 controls<br>. Follow-up: 54 y	. Age: ≤ 52 y<br>. Menopausal status: NR<br>. Invasive and non-invasive cancer<br>. % Invasive: NR<br>. % ER+: NR	. Timing: perinatal<br>. Maternal serum<br>. Method: GC-MS-MS<br>. Lipid-adjusted	DDTs:<br>. *p,p'*–DDT<br>. *o,p'*–DDT<br>. *p,p'*-DDE	. *p,p'*-DDT, quart 4 *vs* quart 1<br>OR=2.2 [NR]<br><br>. *o,p'*-DDT, quart 4 *vs* quart 1<br>OR=2.8 [NR]	. *o,p'*-DDT, quart 4 *vs* quart 1<br>OR =3.7 [1.5-9.0]	. Non-detectable values: NR<br>. Model selection: change in estimate (10%) ; method: descending
Cohn BA et al.<br>2012 [7] and<br>2007 [8]<br>USA (CHDS)	. Prospective<br>. Lag time: not considered<br>. n=129 cases<br>. n=129 controls<br>. Follow-up: median for cases=17 y	. Age: 100 %≤ 50 y, mean=44 y<br>. Menopausal status: NR<br>. Invasive and non-invasive cancer<br>. % Invasive: NR<br>. % ER+: NR	. Timing: post-partum<br>. Serum<br>. Method: GC-ECD<br>. Lipid-adjusted	16 PCBs<br>DDTs:<br>. *p,p'* –DDT<br>. *o,p'* –DDT<br>. *p,p'*-DDE<br><br>*(PCBs individually and summed in PCBs groups)*	*Tert 3 vs Tert 1*<br>. *p,p'*-DDT: OR=2.9 [1.1–8.0]<br>. *o,p'*-DDT: OR=0.4 [0.2–0.8]<br><br>*Quart 4 vs quart 1*<br>. PCB 203/(PCB 167+PCB 187) : OR=3.1 [1.3-7.2]	*Adjusted for breastfeeding and year of blood draw only,* tert 3 *vs* tert 1<br>. *p,p'*-DDT: OR=6.4 [1.9–21.5]<br>. *o,p'*-DDT: OR=0.3 [0.1–0.7]<br><br>*Full adjustment, quart 4 vs quart 1*<br>. PCB 203/(PCB 167+PCB 187): OR=2.8 [1.1-7.1]<br>. PCB 167: OR=0.2 [0.1-0.8]<br>. PCB 187: OR= 0.4 [0.1-1.1]<br>. PCB 203: OR= 6.3 [1.9-21.7]	. Non-detectable values: n=15 pairs for o,p´-DDT, imputed using modeling of other variables<br>. Model selection: series of models, one risk variable at a time for DDTs ; Full model including all PCBs then backward elimination of PCBs based on p-value , other adjustment variables selection method not reported
Iwasaki M et al.<br>2008 [9]<br>Japan	. Prospective<br>. Lag time: not considered<br>. n=139 cases<br>. n=278 controls<br>. Follow-up: 10.7 y	. Age: 51.8 ± 0.60 y<br>. Post-menopausal: 59%<br>. Invasive and non-invasive cancer<br>. % Invasive: NR<br>. % ER+: 32%	. Timing: not considered<br>. Plasma<br>. Method: GC-IDMS<br>. Cholesterol-adjusted	. *p,p'*-DDT<br>. *p,p'*-DDE<br>. HCB<br>. β-HCH	. *p,p'*-DDE, quart 4 *vs* quart 1<br>OR=2.1 [1.1, 4.0]	None	. Non-detectable values: 9% HCB and 27% β-HCH ; imputation: NR<br>. Model selection: NR

Table S3. Studies of POPs measured in peripheral blood and breast cancer risk *(continued)*

Study reference<br>Publication year<br>Location	Design	Patients and tumors characteristics*<br>(mean ± SD)	Exposure assessment	Evaluated POPs	Associated POPs<br>Estimate [95% confidence interval]<br>Without adjustment	 <br>With adjustment**	Comments
**Cohort-nested case-control studies with incidence density (risk-set) sampling**							
Rubin CH et al.<br>2006 [10]<br>USA (Alaska)	. Prospective<br>. Lag time: 3-10 y<br>. n=63 cases<br>. n=63 controls<br>. Follow-up: 3-10 y	. Age: 64% > 54 y<br>. Menopausal status: NR<br>. Invasiveness: NR<br>. % Invasive: NR<br>. % ER+: NR	. Timing: not considered<br>. Serum<br>. Method: GC-ECD<br>. Lipid-adjusted	. 28 PCBs<br>. *o,p'*-DDE<br>. *p,p'*-DDE<br>. *o,p'*-DDT<br>. *p,p'*-DDT<br>. HCB<br>. *trans*-nonachlor<br>. Oxychlordane<br>. Dieldrin<br>. 9 others organochlorines<br>*(individually and sum of DDTs and PCBs)*	. *p,p'*-DDE, Tert 3 *vs* Tert 1<br>OR=1.87 [0.82-4.27]<br><br>. Total PCBs, Tert 3 *vs* Tert 1<br>OR=0.13 [0.03-0.54]<br><br>*ER+ cases*<br>. *p,p'*-DDE, Tert 3 *vs* Tert 1<br>OR=1.76 [0.66-4.68]	. *p,p'*-DDE, Tert 3 *vs* Tert 1<br>OR=1.43 [0.46-4.47]<br><br>. Total PCBs, Tert 3 *vs* Tert 1<br>OR=0.42 [0.07-2.38]	. Only compounds with >50% of detectable values considered individually<br>. Model selection: NR
Wolff MS et al.<br>2000 [11]<br>USA (NYUWHS)	. Prospective<br>. Lag time: 6 months<br>. n=148 cases<br>. n=295 controls<br>. Follow-up: 3-9 y	. Age: median=60.3 y, range [36-71]<br>. Post-menopausal: 63.5%<br>. Invasiveness: NR<br>. % Invasive: NR<br>. % ER+: 57/89 cases	. Timing: not considered<br>. Serum<br>. Method: GC-ECD<br>. Lipid-adjusted	. DDE<br>. PCBs	None	None	. Non-detectable values: NR<br>. Model selection: NR
Terrell ML et al.<br>2016 [12]<br>USA	. Prospective<br>. Lag time: not considered<br>. n=51 cases<br>. n=202 controls<br>. Follow-up: max=28y	. Age: mean=61.2 ± 13.9 y<br>range [28.9-90.3]<br>. Menopausal status: NR<br>. Invasive cancers<br>. % Invasive: 100%<br>. % ER+: NR	. Timing: not considered<br>. Serum<br>. Method: GC-ECD<br>. Lipid-adjustment: none	. PBB mixture (mostly PBB 153)	NR	. PBB mixture, continuous<br>OR=1.2 [1.0-1.5]	. Non-detectable values: NR<br>. Model selection: change in estimate (10%) ; ascending
Ward E et al.<br>2000 [13]<br>Norway	. Prospective<br>. Lag time: ≥ 2 years<br>. n=150 cases<br>. n=150 controls<br>. Follow-up: 2-20 y	. Age: 60.4% < 50 y<br>. Pre-menopausal: 60.4%<br>. Invasive cancers<br>. % Invasive: 99.3%<br>. % ER+: 44/84	. Timing: not considered<br>. Serum<br>. Method: GC-ID-HRMS<br>. Lipid-adjusted	71 compounds:<br>. 22 dioxins, furans, and non-ortho co-planar PCBs<br>. 36 other PCBs<br>. 13 pesticides<br>*(individually and sum of dioxins, furans and PCBs)*	None	None	. Non-detectable values for analytes with >90% sample above LD imputed square root of 2 of the LD<br>. Non-detectable values for analytes with <90% sample above LD: dichotomous analysis detectable *vs* non-detectable<br>. Model selection: NR
Helzlsouer KJ et al.<br>1999 [14]<br>USA (CLUE 1 and 2)	. Prospective<br>. Lag time: not considered<br>. n=340 cases<br>. n=340 controls<br>. Follow-up: 5-20y	. Age: NR<br>. Menopausal status: NR<br>. Invasive and non-invasive cancer<br>. % Invasive: 74%<br>. % ER+: 44%	. Timing: not considered<br>. Serum and plasma<br>. Method: GC-ECD<br>. Lipid-adjusted	. Total PCBs (22-26 PCBs)<br>. Total DDE/DDT	None	NR	. Non-detectable values: NR<br>. Model selection: NR<br>. Same results when analyses by time from blood sampling to time of diagnosis, menopausal status and ER-status
Dorgan JF et al.<br>1999 [15]<br>USA	. Prospective<br>. Lag time: not considered<br>. n=105 cases<br>. n=208 controls<br>. Follow-up:<br>median =2.7 y<br>range=27days-9.5y	. Age: 59.7 ±‹10.3 y<br>. Post-menopausal: 85.7%<br>. Invasive and non-invasive cancer<br>. % Invasive: 71%<br>. % ER+: NR	. Timing: not considered<br>. Serum<br>. Method: GC-ECD<br>. Lipid-adjusted	. 27 PCBs<br>. 5 DDT analogs<br>. 13 other organochlorines<br><br>*(individually and sum of DDTs and PCBs)*	NR	. *p,p'*-DDT, quart 4 *vs* quart 1<br>OR =0.4 [0.2-1.0]<br><br>. HCB, quart 4 *vs* quart 1 :<br>OR=2.3 [1.0-5.0]	. Non-detectable values for analytes with <50% sample above LD: dichotomous analysis detectable vs non-detectable<br>. Model selection: NR<br>. For HCB: association maintained only for cases diagnosed <2.7y after blood draw
Hoyer AP et al.<br>2000 [16]<br>1998 [17]<br>Denmark	. Prospective<br>. Lag time: not considered<br>. n=240 cases<br>. n=477 controls<br>. Follow-up: 17y	. Age: mean=66 y<br>. Menopausal status: NR<br>. Invasive cancer<br>. % Invasive: 100%<br>. % ER+: NR	. Timing: not considered<br>. Serum<br>. Method: GC-ECD<br>. Lipid-adjusted	. 28 PCBs<br>. 5 DDT analogs<br>. 13 other organochlorines<br><br>*(individually and sum of DDTs and PCBs)*	. Dieldrin, quart 4 *vs* quart 1 :<br>OR= 2.3 [1.3-3.8]	. Dieldrin, quart 4 *vs* quart 1<br>OR=2.1 [1.2-3.6]<br><br>. PCB 118, quart 4 *vs* quart 1<br>OR=2.3 [1.1-4.8]	. Non-detectable values: NR<br>. Model selection: backwards stepwise procedure<br>. Stronger results for dieldrin after exclusion of women who developed breast cancer within 5 years of serum sampling

Table S3. Studies of POPs measured in peripheral blood and breast cancer risk *(continued)*

Study reference<br>Publication year<br>Location	Design	Patients and tumors characteristics*<br>(mean ± SD)	Exposure assessment	Evaluated POPs	Associated POPs<br>Estimate [95% confidence interval]<br>Without adjustment	 <br>With adjustment**	Comments
**Cohort-nested case-control studies with incidence density (risk-set) sampling**							
Krieger N et al.<br>1994 [18]<br>USA	. Prospective<br>. Lag time: ≥ 6 months<br>. n=150 cases<br>. n=150 controls<br>. Follow-up: 14.2 ± 6.3 y	. Age: NR<br>. Post-menopausal: 79%<br>. Invasiveness: NR<br>. % Invasive: NR<br>. % ER+: 44/67 cases	. Timing: not considered<br>. Serum<br>. Method: GC-ECD<br>. Lipid-adjustment : NR	. PCBs (total)<br>. DDE	None	None	. Non-detectable values: NR<br>. Model selection: none<br>(*a priori* variable selection and variables univariately associated with risk)
Wolff MS et al.<br>1993 [19]<br>USA (NYUWHS)	. Prospective<br>. Lag time: none<br>(*prevalent cases diagnosed within 6 months of blood draw*)<br>. n=58 cases<br>. n=171 controls<br>. Follow-up: 6 months	. Age: NR<br>. Post-menopausal: 59%<br>. Invasiveness: NR<br>. % Invasive: NR<br>. % ER+: NR	. Timing: not considered<br>. Serum<br>. Method: GC-ECD<br>. Lipid-adjustment : NR	. Higher PCBs (total)<br>. *p,p'*-DDE	. *p,p'*-DDE: 35% higher in cases than controls	. *p,p'*-DDE, quint 5 *vs* quint 1<br>OR =3.7 [1.0-13.5]	. Non-detectable values: NR<br>. Model selection: change in estimate (15%) ; method : NR
**Cohort-nested case-control studies with exclusive (cumulative density) sampling**							
Laden F et al.<br>2002 [20] and<br>2001 [21]<br>Hunter DJ et al.<br>1997 [22]<br>USA (Nurses' Health Study)	. Prospective<br>. Lag time: not considered<br>. n=378 cases<br>. n=378 controls<br>. Follow-up: 4-5 y	. Age: median= 60 y, range [43-69]<br>. Post-menopausal: 80%<br>. Invasive and non-invasive cancer<br>. % Invasive: 90%<br>. % ER+: 59% (of 236 cases)	. Timing: not considered<br>. Plasma<br>. Method: GC-ECD<br>. Lipid-adjusted	. DDE<br>. Total PCBs<br>*(sum of the 16 more chlorinated PCBs)*<br>. PCB 118<br>. PCB 138<br>. PCB 153<br>. PCB 180	*Quint 5 vs quint 1 (matched)*<br>. DDE: OR= 0.8 (0.5–1.2]<br>. Total PCBs: OR= 0.8 [0.5-1.4]<br>. PCB 118: OR=0.8 [0.4-1.3]<br>. PCB 138: OR=0.9 [0.6-1.5]<br>. PCB 153: OR=0.9 [0.5-1.5]<br>. PCB 180: OR= 1.0 [0.6-1.7]<br><br>*Tert 3 vs tert 1*<br>. Total PCBs: OR=2.8 [1.0-7.4]<br>*(post-menopausal women with CYP1A1-exon7 variants)*	*Quint 5 vs quint 1*<br>. DDE: OR=0.8 [0.5-1.4]<br>. Total PCBs: OR=0.8 [0.5-1.5]<br>. PCB 118: OR=0.7 [0.4-1.2]<br>. PCB 138: OR=0.9 [0.5-1.5]<br>. PCB 153: OR=0.8 [0.5-1.5]<br>. PCB 180: OR=1.0 [0.6-1.8]<br><br>*Tert 3 vs tert 1*<br>. Total PCBs: OR=2.8 [1.0–7.8]<br>*(post-menopausal women with CYP1A1-exon7 variants)*	. Non-detectable values: exclusion of samples when the major PCBs (118, 138, 153) non detectable; imputed by regression when minor PCBs<br>. Model selection: NR<br>. Possible effect modification by CYP1A1 polymorphism<br>. Similar results when restricted to invasive cancers or ER-positive tumors
Hoyer AP et al.<br>2001 [23] and<br>2002 [24]<br>Denmark	. Prospective<br>. Lag time: not considered<br>. n=161 cases<br>. n=318 controls<br>. Follow-up: 17-21 y	. Age: NR<br>. Menopausal status: NR<br>. Invasiveness: NR<br>. % Invasive: NR<br>. % ER+: 72%	. Timing: not considered<br>. Serum<br>. Method: GC-ECD<br>. Lipid-adjusted	. Total of 28 PCBs<br>. HCB<br>. Dieldrin<br>. *p,p'*-DDE<br>. *p,p'*-DDT<br>. Total DDT	NR	*ER-negative cases, no adjustment for disease stage, quart 4 vs quart 1:*<br>. Dieldrin : OR =7.6 [1.3-46.1]	. Non-detectable values: NR<br>. Model selection: covariates statistical significance ; backward step-wise procedures
Hoyer AP et al.<br>2000 [25]<br>Denmark	. Prospective<br>. Lag time: 3-7 y<br>. n=155 cases<br>. n=274 controls<br>. Follow-up: 14-16 y	. Age: mean=66 y<br>. Menopausal status: NR<br>. Invasiveness: NR<br>. % Invasive: NR<br>. % ER+: NR	. Timing: not considered<br>. Serum<br>. Method: GC-ECD<br>. Lipid-adjusted	PCBs: 74, 118, 138, 153, 180<br>Organochlorines:<br>. β-HCH<br>. Dieldrin<br>. *p,p'*-DDT<br>. *p,p'*-DDE<br><br>*(individually and sum of DDTs and PCBs)*	None	Average exposure over 3-7 y<br>. *p,p'*-DDT, quart 4 *vs* quart 1<br>OR=3.6 [1.1-12.2]<br><br>. PCB 138, quart 4 *vs* quart 1<br>OR=2.1 [1.0-4.4]<br><br>Exposure at baseline<br>. Dieldrin, quart 4 *vs* quart 1<br>OR=2.1 [1.2-3.6]	. Non-detectable values: assigned a zero value<br>. Model selection: covariates associated with change in serum organochlorines ; backward step-wise procedures

Table S3. Studies of POPs measured in peripheral blood and breast cancer risk *(continued)*

Study reference<br>Publication year<br>Location	Design	Patients and tumors characteristics*<br>(mean ± SD)	Exposure assessment	Evaluated POPs	Associated POPs<br>Estimate [95% confidence interval]<br>Without adjustment	 <br>With adjustment**	Comments
**Population-based case-control studies (not nested in a defined cohort)**							
Morgan M et al.<br>2017 [26]<br>Xu X et al.<br>2010 [27]<br>USA (NHANES)	. Cross-sectional<br>. Lag time: NA (prevalent breast cancers)<br>. n=62 cases<br>. n=2189 controls<br>. Follow-up: NA	. Age: 57.5% ≥ 65 y<br>. Post-menopausal: NR<br>. Invasiveness: NR<br>. % Invasive: NR<br>. % ER+: NR	. Timing: after diagnosis<br>. Serum<br>. Method: GC-ID-HRMS<br>. Lipid-adjusted	*PCBs above the LOD in at least 60% of subjects*<br>. PCBs: 74, 99, 118, 138, 153, 180<br>. Non-Dioxin-like PCBs (99+138+153+180)<br>. Dioxin-like PCBs: 74+118<br><br>*Organochlorines above the LOD in at least 50% of subjects*<br>. β-HCH<br>. *p,p'*-DDE<br>. Oxychlordane<br>. *trans*-nonachlor<br>. Heptachlor epoxide<br>. Dieldrin	*Above vs below LOD*<br>. PCB 74: OR=5.8 [2.6-13.3]<br>. PCB 99: OR=3.1 [1.6-6.0]<br>. PCB 118: OR=4.3 [2.0-9.7]<br>. PCB 138: OR=7.4 [3.1-17.4]<br>. PCB 153: OR=9.2 [2.3-36.7]<br>. PCB180: OR=10.6 [3.0-37.1]<br>. Dioxin-like: OR=1.5 [1.3-1.8]<br>. Non-Dioxin-like: OR=1.3 [1.2-1.4]<br><br>*Age-adjusted, tert 3 vs tert 1*<br>. β-HCH: OR= 1.7 [0.4-6.5]<br>. *p,p'*-DDE: OR= 0.9 [0.3-2.6]<br>. Dieldrin : OR= 1.0 [0.3-3.4]<br>. Oxychlordane: OR=3.0 (0.9–10.0]<br>. *trans*-nonachlor: OR=2.67 [0.6-11.2]<br>. Heptachlor epoxide: OR= 1.2 [0.5-2.8]	*Above vs below LOD*<br>. PCB 74: OR=2.6 [0.6-12.0]<br>. PCB 99: OR=1.7 [0.6-3.4]<br>. PCB 118: OR=2.0 [0.5-8.4]<br>. PCB 138: OR=2.9 [1.1-7.3]<br>. PCB 153: OR=3.6 [0.7-18.6]<br>. PCB 180: OR=4.5 [1.1-18.6]<br>. Dioxin-like: OR=1.1 [0.8-1.5]<br>. Non-Dioxin-like: OR=1.1 [0.9-1.3]<br><br>*Tert 3 vs tert 1*<br>. β-HCH: OR=2.3 [0.5-10.3]<br>. *p,p'*-DDE: OR=1.2 [0.4-3.5]<br>. Dieldrin : OR=1.0 [0.3-4.2]<br>. Oxychlordane: OR=2.6 [0.7-8.8]<br>. *trans*-nonachlor: OR=2.6 [0.7-10.4]<br>. Heptachlor epoxide: OR=1.1 [0.4-2.9]	. Non-detectable values: assigned a value of zero or imputed the LOD value divided by the square root of 2<br>. Model selection: NR for PCBs; 2 different fully adjusted models, only the best fitted model based on smaller AIC reported for other organochlorines<br>. Similar results when PCBs analysed in tertiles
Pastor-Barriuso R et al. 2016 [28]<br>Spain	. Retrospective<br>. Lag time: NA<br>. n=186 cases<br>. n=196 controls<br>. Follow-up: NA	. Age: 59.7 ± 11.1<br>. Post-menopausal: 83%<br>. Invasive and non-invasive cancer<br>. % Invasive: 89%<br>. % ER+: NR	. Timing: after diagnosis<br>. Serum<br>. Method: HPLC and HR-GC-ECD<br>. Lipid-adjustment : NR	. PCB-138<br>. PCB-153<br>. PCB-180<br>. HCB<br>. *p,p'*-DDE<br>. Total effective xenoestrogen burden (α- and β-HPLC fractions)	NR	. TEXB-α, tert 3 *vs* tert 1 OR=3.5 [1.5- 8.0]<br><br>. TEXB- β, tert 3 *vs* tert 1 OR= 4.0 [1.9, 8.6]<br><br>*(after mutual adjustment, association maintained only for fraction β)*	. Non-detectable values: <10% of samples for each analyte ; imputed the limit of detection value divided by the square root of 2<br>. Model selection: none (*3 a priori models*)<br>. HLPC-β includes endogenous estrogens
Tang M et al.<br>2014 [29]<br>China	. Retrospective<br>. Lag time: NA<br>. n=78 cases<br>. n=72 controls<br>. Follow-up: NA	. Age: NR<br>. Post-menopausal: NR<br>. Invasiveness : NR<br>. % Invasive: NR<br>. % ER+: NR	. Timing: NR<br>. Serum<br>. Method: GC-MS<br>. Lipid-adjustment : NR	. p,p'-DDE<br>. p,p'-DDT<br>. ΣDDT	NR	*Above vs below median*<br>. p,p'-DDE: OR=2.5 [1.2-5.3]<br>. p,p'-DDT: OR=1.4 [0.7-2.9]<br>. ΣDDT: OR=2.0 [1.0–4.0]	. Non-detectable values: NR<br>. Model selection: NR
Gatto NM et al.<br>2007 [30]<br>USA (Women's CARE Study)	. Retrospective<br>. Lag time: NA<br>. n=355 cases<br>. n=327 controls<br>. Follow-up: NA	. Age: 48.2 ± 8.3 y<br>. Post-menopausal: 41%<br>. Invasive cancer<br>. % Invasive: 100%<br>. % ER+: 183/338 cases	. Timing: after diagnosis<br>. Serum<br>. Method: GC-ECD<br>. Lipid-adjusted	. DDE<br>. PCBs	NR	*Cases with no history of chemotherapy, n=94*<br>. PCB, > 0.47 µg/L *vs* non-detectable: OR=1.9 [1.1–3.4]	. Non-detectable values: PCBs only, 41% of cases and 42% controls ; assigned a value of zero<br>. Model selection: change in estimate (15%) ; ascending
Li Y et al.<br>2005 [31]<br>USA	. Retrospective<br>. Lag time: NA<br>. n=612 cases<br>. n=599 controls<br>. Follow-up: NA	. Age: NR<br>. Post-menopausal: 49%<br>. Invasive cancers<br>. % Invasive: 100%<br>. % ER+: NR	. Timing: after diagnosis<br>. Plasma<br>. Method: GC-ECD<br>. Lipid-adjusted	PCBs: 99, 105, 118, 153<br><br>*(individually and summed)*	NR	None	. Non-detectable values: imputed the detection limit value divided by the square root of 2<br>. Model selection: NR<br>. Possible effect modification by CYP1A1-M2 genotype
Millikan R et al.<br>2000 [32]<br>USA	. Retrospective<br>. Lag time: NA<br>. n=748 cases<br>. n=659 controls<br>. Follow-up: NA	. Age: mean=50.2 y, range [23-74]<br>. Post-menopausal: 49%<br>. Invasive cancers<br>. % Invasive: 100%<br>. % ER+: NR	. Timing: after diagnosis<br>. Plasma<br>. Method: GC-ECD<br>. Lipid-adjusted	. Total of 35 PCBs<br>. Total of PCBs 118, 138, 153, and 180<br>. Chlorinated PCBs<br>. Less chlorinated PCBs<br>. *p,p'*-DDE<br>. *o,p'*-DDE<br>. DDT	NR	*Fully adjusted models*<br>. Total PCBs, tert 3 vs tert 1<br>African-Americans: OR=1.7 [1.0–3.0]<br>African-Americans in the highest third of BMI: OR=4.9 [1.6-14.8]	. Non-detectable values: 99% for *o,p'*-DDE, analyte not included in analyses ; other analytes: imputed detection limit divided by the square root of 2<br>. Model selection: *a priori* final model

Table S3. Studies of POPs measured in peripheral blood and breast cancer risk *(continued)*

Study reference<br>Publication year<br>Location	Design	Patients and tumors characteristics* (mean ± SD)	Exposure assessment	Evaluated POPs	Associated POPs Estimate [95% confidence interval]: Without adjustment	With adjustment**	Comments
**Population-based case-control studies (not nested in a defined cohort)**							
Moysich KB et al.<br>1999 [33]<br>USA	. Retrospective<br>. Lag time: NA<br>. n=154 cases<br>. n=191 controls<br>. Follow-up: NA	. Age: NR<br>. Post-menopausal: 100%<br>. Invasiveness: NR<br>. % Invasive: NR<br>. % ER+: NR	. Timing: before chemotherapy or radiation within 3 months of surgery<br>. Serum<br>. Method: GC-ECD<br>. Lipid-adjusted	. 73 PCBs<br>. DDE<br>. HCB<br>. Mirex<br>. Total PCBs *(sum 56 PCBs)*<br>*(individual and sum of PCBs)*	. Total PCBs, > vs < median OR =2.2 [1.0-4.8]<br>*(when at least one allele of CYP1A1 with isoleucine-to-valine substitution in exon 7)*	*Never lactated women:*<br>. Total PCBs, tert 3 *vs* tert1 OR=2.9 [1.0-7.3]<br>. Number of detected peaks OR =3.3 [1.0-11.3]<br>. Moderately chlorinated OR= 3.6 [1.1-8.6]<br>*At least one allele of CYP1A1 with Ile-to-Val exon substitution*<br>. Total PCBs, > *vs* < median OR=2.9 [1.2-7.4]	. Non-detectable values: assigned a zero value<br>. Model selection: change in estimate (15%); method: NR<br>. Possible effect modification by CYP1A1 polymorphism
Gammon MD et al.<br>2002 [34]<br>USA	. Retrospective<br>. Lag time: NA<br>. n=646 cases<br>. n=429 controls<br>. Follow-up: NA	. Age: NR<br>. Post-menopausal: 61%<br>. Invasive and non-invasive cancers<br>. % Invasive: 72%<br>. % ER+: 39%	. Timing: after diagnosis<br>. Serum<br>. Method: GC-ECD<br>. Lipid-adjusted	. 24 PCBs<br>. *p,p'*-DDE<br>. *p,p'*DDT<br>. Oxychlordane<br>. *trans*-nonachlor<br>. Dieldrin<br>*(individually and summed)*	None	None	. Non-detectable values: imputed the lowest observed positive value<br>. Model selection: best fitting model by -2 log likelihood ratio test, descending
Romieu I et al.<br>2000 [35]<br>Mexico	. Retrospective<br>. Lag time: NA<br>. n=120 cases<br>. n=126 controls<br>. Follow-up: NA	. Age: 52 ± 14 y<br>. Post-menopausal: 53%<br>. Invasiveness: NR<br>. % Invasive: NR<br>. % ER+: NR	. Timing: after diagnosis<br>. Plasma<br>. Method: GC-ECD<br>. Lipid-adjusted	. DDT<br>. DDE	Higher in cases:<br>. DDE	*Age-adjusted:*<br>. DDE, continuous OR =1.6 [1.1-2.3] per log unit of lipid-adjusted DDE<br>*Fully adjusted:*<br>. DDE, quart 4 *vs* quart 1 OR = 3.8 [1.1-12.8]	. Non-detectable values: NR<br>. Model selection: NE, 3 different models<br>. Stronger associations in post-menopausal women
**Hospital-based case-control studies with community-controls**							
Arrebola JP et al.<br>2015 [36]<br>Tunisia	. Retrospective<br>. Lag time: NA<br>. n=69 cases<br>. n=54 controls<br>. Follow-up: NA	. Age: 49.9 ± 11.0 y<br>. Post-menopausal: 51 %<br>. Invasiveness: NR<br>. % Invasive: NR<br>. % ER+: NR	. Timing: before surgery or chemotherapy<br>. Serum<br>. Method: GC-ECD<br>. Lipid-adjusted	. PCBs: 138, 153, 180<br>. p,p'-DDE<br>. HCB<br>. β-HCH<br>. α-endosulfan<br>. Endosulfan ether<br>. Heptachlor<br>. Oxychlordane	. β-HCH: OR=1.1 [1.0-1.2]<br>. Heptachlor: OR=1.1 [1.0-1.2]<br>. PCB 138: OR=1.1 [1.0-1.1]<br>. PCB 180: 1.1 [1.0-1.1]<br>. p,p'-DDE: OR=1.3 [1.1-1.8]	. β-HCH: OR=1.2 [1.1-1.3]<br>. Heptachlor: OR=1.1 [1.0-1.2]<br>. p,p'-DDE: OR=1.7 [1.1-3.1]<br>*(all for continuous exposure)*	. Non-detectable values: imputed a random value between zero and the detection limit value<br>. Model selection: change in estimate (10%); method :NR
Boada LD et al.<br>2012 [37]<br>Gran Canaria Island	. Retrospective<br>. Lag time: NA<br>. n=121 cases<br>. n=103 controls<br>. Follow-up: NA	. Age: 58.0 ± 11.7 y<br>. Post-menopausal: 18.2%<br>. Invasiveness: NR<br>. % Invasive: NR<br>. % ER+: NR	. Timing: after diagnosis<br>. Serum<br>. Method: GC-ECD<br>. Lipid-adjusted	. p,p´-DDT<br>. o,p´-DDT<br>. p,p´-DDE<br>. o,p´-DDE<br>. p,p´-DDD<br>. o,p´-DDD<br>. Aldrin<br>. Dieldrin<br>. Endrin<br>. γ-HCH<br>*(individually and summed)*	Higher in cases:<br>. Aldrin<br>. *p,p'*-DDE<br>. *p,p'*-DDD<br>. *p,p'*-DDT<br>. Total DDTs<br>. Total organochlorines<br>Higher in cases >45y<br>. Aldrin<br>. p,p-DDE<br>. p,p-DDD<br>. p,p-DDT<br>. Total organochlorines<br>Higher in controls:<br>. Total Cyclodienes	None	. Non-detectable values: *o,p'*-isomes of DDT, DDE and DDD detected in a small percentage of samples (not reported);<br>. Model selection: *a priori* variables selection

Table S3. Studies of POPs measured in peripheral blood and breast cancer risk *(continued)*

Study reference<br>Publication year<br>Location	Design	Patients and tumors characteristics*<br>(mean ± SD)	Exposure assessment	Evaluated POPs	Associated POPs Estimate [95% confidence interval]<br>Without adjustment	 With adjustment**	Comments
**Hospital-based case-control studies with community-controls**							
Bonefeld-Jorgensen EC et al. 2011 [38]<br>Greenland	. Retrospective<br>. Lag time: NA<br>. n=31 cases<br>. n=115 controls<br>. Follow-up: NA	. Age: median=50 y, range [29-80]<br>. Post-menopausal: 82%<br>. Invasiveness: NR<br>. % Invasive: NR<br>. % ER+: NR	. Timing: after diagnosis<br>. Plasma<br>. Method: GC-ECD ; HPLC-MS-MS (PFCs)<br>. Lipid-adjusted	. 12 PCBs<br>. 10 PFCs<br>. 8 other organochlorines<br><br>*(individually and summed)*<br><br>. Xeno-estrogenic and androgenic transactivities of F1 fraction	Higher in cases :<br>. PFOS<br>. PFOA<br>. Sum PFSAs<br>. Sum PFCAs<br>. Sum of all analytes<br>. Xenoandrogenic transactivity OR=8.5 [1.6-46.8]	. Xenoandrogenic transactivity OR= 44.1 [2.0-975.7]	. Non-detectable values: NR<br>. Model selection: change in estimate (15%); ascending
Li JY et al. 2006 [39]<br>China	. Retrospective<br>. Lag time: NA<br>. n=104 cases<br>. n=154 controls<br>. Follow-up: NA	. Age: 48.6 ± 11.0<br>. Post-menopausal: 55%<br>. Invasiveness: NR<br>. % Invasive: NR<br>. % ER+: NR	. Timing: NR<br>. Serum<br>. Method: GC-EDC<br>. Lipid-adjustment : NR	. *p,p'*–DDT<br>. *p,p'*-DDE<br>. *o,p'*-DDT<br>. *p,p'*-DDD<br>. α-, β-, γ- and δ-HCH	None	*OR > 2 [NR] (n=90/136)*<br>. *p,p'*-DDT, *p,p'*-DDD, δ -HCH<br><br>*Premenopausal,* high vs reference, *(n=90/136):*<br>. *p,p'*-DDT: OR= 3.6 [NR]<br>. *p,p'*-DDD: OR=5.7 [NR]<br>. β-HCH: OR=3.1 [NR]<br><br>*At least one variant allele of CYPIAI m2 genotype:*<br>. *p,p'*-DDT: OR=4.4 [1.1-17.0]	. Non-detectable values: NR<br>. Model selection: NR<br>. *p,p'*–DDT categorized as high when > upper quartile of controls
Pavuk M et al. 2003 [40]<br>Slovakia	. Retrospective<br>. Lag time: NA<br>. n=24 cases<br>. n=88 controls<br>. Follow-up: NA	. Age: median=51.5 y, range [26-73]<br>. Post-menopausal: 83%<br>. Invasiveness: NR<br>. % Invasive: NR<br>. % ER+: NR	. Timing: after surgery ± chemotherapy and/or radiotherapy<br>. Serum<br>. Method: GC-EDC<br>. Lipid-adjusted	. 15 PCBs<br>. *p,p'*-DDE<br>. *p,p'*–DDT<br>. HCB<br>*(individual and sum of PCBs)*	Lower in cases:<br>. PCBs group 1 (28+52+101)<br>Higher in cases:<br>. DDE<br>. DDT<br>. HCB	. PCBS group 1 (28+52+101), tert 3 *vs* tert 1 OR=0.2 [0.1–1.0]	. Non-detectable values: imputed half the detection limit value<br>. Model selection: statistical significance (0.05) and/or change in estimate (10%) ; method: NR
Soliman AS et al. 2003 [41]<br>Egypt	. Retrospective<br>. Lag time: NA<br>. n=69 cases<br>. n=53 controls<br>. Follow-up: NA	. Age: 39.8 ± 7.1 y<br>. Premenopausal<br>. Invasiveness: NR<br>. % Invasive: NR<br>. % ER+: NR	. Timing: before chemotherapy and radiotherapy<br>. Serum<br>. Method: GS-ECD<br>. Lipid-adjustment : None	. 19 organochlorines	NR	None	. Non-detectable values: assigned zero values<br>. Model selection: regression tree-based method
Dello Lacovo R et al. 1999 [42]<br>Italy	. Retrospective<br>. Lag time: NA<br>. n=170 cases<br>. n=195 controls<br>. Follow-up: NA	. Age: 54.2 ±10.1<br>. Post-menopausal: 64.5%<br>. Invasiveness: NR<br>. % Invasive: NR<br>. % ER+: NR	. Timing: before chemotherapy and radiotherapy<br>. Serum<br>. Method: GC-ECD<br>. Lipid-adjustment : NR	. β-HCH<br>. Heptachlor<br>. *p,p'*-DDE<br>. *p,p'*-DDT<br>. Endrin	None	None	. Non-detectable values: NR<br>. Model selection: NR

Table S3. Studies of POPs measured in peripheral blood and breast cancer risk *(continued)*

Study reference<br>Publication year<br>Location	Design	Patients and tumors characteristics*<br>(mean ± SD)	Exposure assessment	Evaluated POPs	Associated POPs<br>Estimate [95% confidence interval]<br>Without adjustment	 <br>With adjustment**	Comments
**Hospital-based case-control studies with community-controls**							
Mendonça GAS et al. 1999 [43]<br>Brazil	. Retrospective<br>. Lag time: NA<br>. n=177 cases<br>. n=350 controls<br>. Follow-up: NA	. Age: median=58 y, range [30-75]<br>. Post-menopausal: 76.8%<br>. Invasive cancers<br>. % Invasive: 100%<br>. % ER+: NR	. Timing: before surgery<br>. Serum<br>. Method: GC-ECD<br>. Lipid-adjustment : NR	. HCB<br>. α-, β- and γ-HCH<br>. Heptachlor<br>. Heptachlor epoxide<br>. Dieldrin<br>. Endrin<br>. p-p'-DDE<br>. o-p'-DDE<br>. o-p'-DDT<br>. p-p'-DDT<br>. p-p'-DDD	None	None	. Non-detectable values: NR<br>. Model selection: NR
**Hospital-based case-control studies with both community-controls and hospital-controls**							
Wielsøe M. et al 2017 [44]<br>Wilsoe 2018 [45]<br>Greenland	. Retrospective<br>. Lag time: NA<br>. n=77 cases<br>. n=84 controls<br>. Follow-up: NA	. Age: mean 52.0 y<br>. Post-menopausal: 64 %<br>. Invasiveness: NR<br>. % Invasive: NR<br>. % ER+: 65%	. Timing: before any treatment<br>. Serum<br>. Method: LC-MS-MS<br>. Lipid-adjusted	. 14 PCBs<br>. 9 PBDES<br>. 17 PFAAs<br>. PBB153<br>. 11 other organochlorines<br><br>*(individually and summed in categories)*	. Total organochlorines<br>. *cis*-nonachlor<br>. *trans*-nonachlor<br>. mirex<br>. Oxychlordane<br>. *p,p'*-DDE<br>. PCBs: 9, 118, 138, 153, 170, 180, 183,187<br>. Total PFAA<br>. PFHpA<br>. PFOA<br>. Total PFSA<br>. PFHxS<br>. PFOS	*ORs from 2.4 to 5.5, tert 3 vs tert 1*<br>. Total PCBs<br>. PCBs: 138,153, 170, 183<br>. Total PFAA<br>. PFOA<br>. PFDA<br>. Total PFSA<br>. PFHxS<br>. PFOS	. Non-detectable values: excluded form single compound analyses if >50% ; imputed detection limit divided by two<br>. Model selection: change in estimate (10%), method : descending<br>. Hospital controls include patients with benign breast lesions<br>. No differences in levels of measured analytes between ER-positive and ER negative tumors
Zhang Y et al. 2004 [46]<br>USA	. Retrospective<br>. Lag time: NA<br>. n=374 cases<br>. n=406 controls<br>. Follow-up: NA	. Age: NR<br>. Post-menopausal: 77%<br>. Invasiveness: NR<br>. % Invasive: NR<br>. % ER+: NR	. Timing: after diagnosis<br>. Serum<br>. Method: ~~GS~~GC<br>. Lipid-adjusted	. PCBs : 74, 118, 138, 153, 156, 170, 180, 183, 187	NR	. Total PCBs, > *vs* < median<br>OR = 2.4 [1.1-5.0]<br>*(CYP1A1 m2 variant in post-menopausal women)*	. Non-detectable values: NR<br>. Model selection: NR<br>. Hospital controls: benign breast disease excluding atypical hyperplasia, normal breast tissue<br>. Possible effect modification by CYP1A1 m2 variants
Demers A et al. 2000 [47] and 2002 [48]<br>Canada	. Retrospective<br>. Lag time: NA<br>. n=315 cases<br>. n=523 controls<br>. Follow-up: NA	. Age: 53 ± 9 y<br>. Post-menopausal: 70%<br>. Invasive cancers<br>. % Invasive: 100%<br>. % ER+: 70%	. Timing: before chemotherapy or radiotherapy<br>. Plasma<br>. Method: GC-ECD<br>. Lipid-adjusted	. 14 PCBs<br>. *p,p'*-DDT<br>. *p,p'*-DDE<br>. *cis*-nonachlor<br>. *trans*-nonachlor<br>. Oxychlordane<br>. α- and γ-chlordane<br>. HCB<br>. β-HCH<br>. Mirex<br>. Aldrin	Higher in cases:<br>. PCB 99<br>. PCB 118<br>. PCB 156	. PCB 118, quart 4 *vs* quart 1<br>OR=1.6 [1.0-2.5]<br><br>. PCB 156, quart 4 *vs* quart 1<br>OR=1.8 [1.1-2.9]<br><br>. Mono-ortho-PCBs, quart 4 *vs* quart 1<br>OR=2.0 [1.2-3.3]	. Non-detectable values: >30% for 28, 52, 101, 105, 128 (not analysed)<br>. Model selection: change in estimate (10%) ; method: NR<br>. Hospital controls: no gynecologic illnesses<br>. Stronger associations in pre-menopausal women (PCB 118 and 156)
Zheng T et al. 2000 [49]<br>USA	. Retrospective<br>. Lag time: NA<br>. n=475 cases<br>. n=502 controls<br>. Follow-up: NA	. Age: 83% >45 y<br>. Menopausal status: NR<br>. Invasive and non-invasive cancers<br>. % Invasive: NR<br>. % ER+: NR	. Timing: after diagnosis<br>. Serum<br>. Method: GC<br>. Lipid-adjusted	. Total of 9 PCBs<br>. *p,p'*-DDE	None	*Tert 3 vs tert 1*<br>. *p,p'*-DDE: OR=1.0 [0.7-1.4]<br>. PCBs: OR=1.0 [0.7–1.3]	. Non-detectable values: 5% for DDE, 30% for PCBs ; imputed half the detection limit value<br>. Model selection: NR<br>. Type of controls: benign breast diseases excluding atypical hyperplasia and population based controls

Table S3. Studies of POPs measured in peripheral blood and breast cancer risk *(continued)*

Study reference<br>Publication year<br>Location	Design	Patients and tumors characteristics*<br>(mean ± SD)	Exposure assessment	Evaluated POPs	Associated POPs<br>Estimate [95% confidence interval]<br>Without adjustment	 <br>With adjustment**	Comments
**Hospital-based case-control studies with hospital-controls**							
Holmes AK et al.<br>2014 [50]<br>USA (Alaska)	. Retrospective<br>. Lag time: NA<br>. n=75 cases<br>. n=95 controls<br>. Follow-up: NA	. Age: mean=51 y, range [30-88].<br>. Post-menopausal: 64%<br>. Invasive and non-invasive cancer<br>. % Invasive: 83%<br>. % ER+: 53% (PR+)	. Timing: after diagnosis<br>. Serum<br>. Method: NR<br>. Lipid-adjusted	. 34 PCBs<br>. 8 PBDEs<br>. *o,p'*-DDT<br>. *p,p'*-DDT<br>. *p,p'*-DDE<br>. HCB<br>. Mirex<br>. Oxychlordane<br>. β- and γ-HCH<br>. *trans*-nonachlor	None	None	. Non-detectable values: imputed limit of detection divided by the square root of 2<br>. Model selection: NR<br>. Controls : benign breast conditions, fibrocystic changes and hyperplasia
Zhang H et al.<br>2013 [51]<br>China	. Retrospective<br>. Lag time: NA<br>. n=92 cases<br>. n=92 controls<br>. Follow-up: NA	. Age: NR<br>. Post-menopausal: NR<br>. Invasiveness: NR<br>. % Invasive: NR<br>. % ER+: NR	. Timing: after diagnosis<br>. Serum<br>. Method: GC-ECD<br>. Lipid-adjustement: NR	. PCB 28<br>. PCB 52<br>.15 other organochlorines	. β-HCH<br>. *p,p'*-DDE<br>. PCB 52	*OR>2, CI [NR]*<br>. β-HCH<br>. *p,p'*-DDE<br>. PCB 52	. Non-detectable values: NR<br>. Model selection: NR<br>. Type of controls: NR
Recio-Vega R et al. 2011 [52]<br>Mexico	. Retrospective<br>. Lag time: NA<br>. n=70 cases<br>. n=70 controls<br>. Follow-up: NA	. Age: 46.7± 12.3 y<br>. Post-menopausal: 60%<br>. Invasiveness: NR<br>. % Invasive: NR<br>. % ER+: NR	. Timing: before any treatment<br>. Serum<br>. Method: GC-ECD<br>. Lipid-adjusted	. 20 PCBs<br><br>*(individually and summed)*	NR	*ORs from 1.1 (total PCBs) to 7.6 (Group1a):*<br>. PCB 8, 118, 138, 170, 180, 187, 195, 206, 209,<br>. Total PCBs<br>. Group 1a (44+52)<br>. Group 2b (128+138+170)<br>. Group 3 (153+180):<br>. Group 4 (8+195+206+209)<br>. Group 3 (153+180)	. Non-detectable values: assigned zero values<br>. Model selection: statistically significance (0.05) OR change in estimate (10%); method: NR<br>. Controls: negative biopsies. Stronger associations in post-menopausal women: total PCBs, Group 1a, Group 2b, Group 4
Itoh H et al.<br>2009 [53]<br>Japan	. Retrospective<br>. Lag time: NA<br>. n=403 cases<br>. n=403 controls<br>. Follow-up: NA	. Age: 53.7 ± 0.52 y<br>. Post-menopausal: 55%<br>. Invasive cancers<br>. % Invasive: 100%<br>. % ER+: 66%	. Timing: after diagnosis<br>. Serum<br>. Method: GC-ID-HRMS<br>. Lipid-adjusted	. 41 PCBs<br>. *o,p'*-DDT<br>. *p,p'*-DDT<br>. *p,p'*-DDE<br>. HCB<br>. β-HCH<br>. *trans*-nonachlor<br>. *cis*-nonachlor<br>. Oxychlordane<br>. Mirex	Lower in cases:<br>. *o,p'*-DDT<br>. *p,p'*-DDT<br>. *p,p'*-DDE<br>. *trans*-nonachlor<br>. *cis*-nonachlor<br>. Oxychlordane<br>. Mirex<br>. Total PCBs	*Quart 4 vs quart 1*<br>. *cis*-nonachlor:<br>OR=0.4 [0.2-0.9]<br>. Mirex<br>OR=0.4 [0.2–0.8]<br>. Total PCBs<br>OR=0.3 [0.1–0.8]<br>. *trans*-nonachlor<br>OR=0.5 [0.2–1.1]	. Non-detectable values: imputed detection limit values<br>. Model selection: variables correlated with breast cancer risk and serum organochlorines in the data<br>. Type of controls: medical checkup examinees<br>. Stronger association for ER+/PR- tumors<br>. Complete case analyses (n=349)
Yang M et al.<br>2009 [54]<br>South Korea	. Retrospective<br>. Lag time: NA<br>. n=70 cases<br>. n=82 controls<br>. Follow-up: NA	. Age: 46.2 ±10.4 y<br>. Post-menopausal: 37%<br>. Invasiveness : NR<br>. % Invasive: NR<br>. % ER+: NR	. Timing: NR<br>. Serum<br>. Method: HPLC/FD<br>. Lipid-adjustment: NR	Bisphenol A	Median value of BPA higher in cases (0.61 *vs* 0.03 µg/L)	NR	. Non-detectable values: assigned a value of one-half of the minimum detected level<br>. Model selection: None (age-matched)<br>. Type of controls: NR
Siddiqui MKJ et al.<br>2005 [55]<br>India	. Retrospective<br>. Lag time: NA<br>. n=25 cases<br>. n=25 controls<br>. Follow-up: NA	. Age: 49.7 ± 19.6 y, range [32–82]<br>. Post-menopausal: NR<br>. Invasiveness: NR<br>. % Invasive: NR<br>. % ER+: NR	. Timing: after diagnosis<br>. Serum<br>. Method: GC-ECD<br>. Lipid-adjustment: NR	. α-, β-, γ- and δ-HCH<br>. *p,p'*-DDT<br>. *o,p'*-DDT<br>. *p,p'*-DDE<br>. *p,p'*-DDD	Higher in cases:<br>. γ-HCH (2.51 times)	NR	. Non-detectable values: NR<br>. Model selection: NR<br>. Type of controls: benign breast lesions (NR)
Charlier CJ et al.<br>2004 [56]<br>2003 [57]<br>Belgium	. Retrospective<br>. Lag time: NA<br>. n=60 cases<br>. n=60 controls<br>. Follow-up: NA	. Age: 54.8 ± 6.59 y<br>. Post-menopausal: 82%<br>. Invasiveness: NR<br>. % Invasive: NR<br>. % ER+: NR	. Timing: prior to surgery<br>. Serum<br>. Method: GS-MS<br>. Lipid-adjusted	. PCBs: 28, 52, 101, 118, 138, 153, 180<br><br>*(individually and summed)*	Higher in cases:<br>. PCB138<br>. PCB153<br>. Total PCBs	*All measured PCBs included in the main model (mutual adjustment):*<br>. PCB 153, continuous (*log-transformed*)<br>OR=1.8 [1.4-2.5]<br>. Total PCBs: OR=NR	. Non-detectable values: PCBs 28 and 118 undetected (Not reported)<br>. Model selection: statistical significance; backward elimination<br>. Type of controls: cervico-vaginal cytological screening

Table S3. Studies of POPs measured in peripheral blood and breast cancer risk *(continued)*

Study reference<br>Publication year<br>Location	Design	Patients and tumors characteristics*<br>(mean ± SD)	Exposure assessment	Evaluated POPs	Associated POPs<br>Estimate [95% confidence interval]<br>Without adjustment	 <br>With adjustment**	Comments
**Hospital-based case-control studies with hospital-controls**							
Charlier CJ et al.<br>2004 [58]<br>Belgium	. Retrospective<br>. Lag time: NA<br>. n=231 cases<br>. n=290 controls<br>. Follow-up: NA	. Age: 53.6 ± 11.4 y<br>. Post-menopausal: 69%<br>. Invasive and non-invasive cancers<br>. % Invasive: 62%<br>. % ER+: NR	. Timing: prior to surgery<br>. Serum<br>. Method: GS-MS<br>. Lipid-adjusted	. *o,p'*-DDT<br>. *p,p'*-DDT<br>. *o,p'*-DDE<br>. *p,p'*-DDE<br>. HCB	Higher in cases:<br>. *p,p'*DDE<br>. HCB	*Detectable vs non-detectable*<br>. *p,p'*DDE: OR=2.2 [1.4-3.5]<br>. HCB: OR=5.0 [3.0-8.4]<br>*Continuous:*<br>. *p,p'*DDE: OR=1.2 [1.2-1.3]<br>. HCB: OR=1.5 [1.2-1.9]	. Non-detectable values: assigned zero values<br>. Model selection: NR<br>. Type of controls: cervico-vaginal cytological screening
Rusiecki JA et al.<br>2004 [59]<br>USA	. Retrospective<br>. Lag time: NA<br>. n=266 cases<br>. n=347 controls<br>. Follow-up: NA	. Age: 69% ≥ 50 y<br>. Post-menopausal: 65%<br>. Invasiveness: NR<br>. % Invasive: NR<br>. % ER+: 50%	. Timing: after diagnosis<br>. Serum<br>. Method: ~~GS~~GC<br>. Lipid-adjusted	. Total of 9 PCBs	None	None	. Non-detectable values: <30%; treatment in analyses: NR<br>. Model selection: NR<br>. Type of controls: benign breast disease excluding atypical hyperplasia, or normal breast tissue<br>. Stratification by joint ER/PR status
Charlier CJ et al.<br>2003 [60]<br>Belgium	. Retrospective<br>. Lag time: NA<br>. n=159 cases<br>. n=250 controls<br>. Follow-up: NA	. Age: 54.2 ± 12.1 y<br>. Menopausal status: NR<br>. Invasiveness: NR<br>. % Invasive: NR<br>. % ER+: NR	. Timing: prior to surgery<br>. Serum<br>. Method: GS-MS<br>. Lipid-adjusted	. Total DDT<br>. HCB	*Detectable vs none:*<br>. Total DDT: OR=5.4 [1.9-15.2]<br>. HCB: OR=8.7 [2.8-26.6]<br>*Adjusted for breast feeding only, detectable vs none:*<br>. Total DDT: OR=5.6 [1.8-17.7]<br>. HCB: OR=9.1 [2.8-29.4]	NR	. Non-detectable values: assigned zero values<br>. Model selection: NR<br>. Type of controls: routine vaginal cytological examinations
Lopez-Carrillo L et al. 2002 [61]<br>Mexico	. Retrospective<br>. Lag time: NA<br>. n=95 cases<br>. n=95 controls<br>. Follow-up: NA	. Age: range [20-79] y<br>. Menopausal status: NR<br>. Invasive and non-invasive cancers<br>. % Invasive: 90%<br>. % ER+: NR	. Timing: after diagnosis<br>. Serum<br>. Method: GC-ECD<br>. Lipid-adjusted	. PCBs *(Aroclor 1260)*<br>. β-HCH<br>. HCB	None	None	. Non-detectable values: NR<br>. Model selection: NR<br>. Type of controls: clinical wards except gynaecology and oncology
Mathur V et al.<br>2002 [62]<br>India	. Retrospective<br>. Lag time: NA<br>. n=135 cases<br>. n=50 controls<br>. Follow-up: NA	. Age: 26% >50y<br>. Menopausal status: NR<br>. Invasiveness: NR<br>. % Invasive: NR<br>. % ER+: NR	. Timing: after diagnosis<br>. Sample: NR<br>. Method: GC-ECD<br>. Lipid-adjustment : NR	. DDT<br>. DDD<br>. DDE<br>. Heptachlor<br>. Dieldrin<br>. α-, β- and γ-HCH	. All pesticides higher in cases	NR	. Non-detectable values: NR<br>. Model selection: NR<br>. Type of controls: NR
Ahmed MT et al.<br>2002 [63]<br>Egypt	. Retrospective<br>. Lag time: NA<br>. n=43 cases<br>. n=32 controls<br>. Follow-up: NA	. Age: NR<br>. Menopausal status: NR<br>. Invasive cancers<br>. % Invasive: 100%<br>. % ER+: NR	. Timing: after diagnosis<br>. Serum<br>. Method: GC-ECD<br>. Lipid-adjustment : NR	. Total PCBs<br>. DDE	Higher in cases:<br>. PCBs peaks from 16-20	NR	. Non-detectable values: NR<br>. Model selection: NR<br>. Type of controls: benign breast disease and non-breast disease<br>. Similar for breast cancer and benign disease but higher than normal controls
Wolff MS et al.<br>2000 [64]<br>USA	. Retrospective<br>. Lag time: NA<br>. n=175 cases<br>. n=355 controls<br>. Follow-up: NA	. Age: 56 ± 13 y<br>. Post-menopausal: 59%<br>. Invasive and non-invasive cancers<br>. % Invasive: 82%<br>. % ER+: 64%	. Timing: after diagnosis<br>. Serum<br>. Method: GC-ECD<br>. Lipid-adjusted	. 19 PCBs<br>. *trans*-Nonachlor<br>. *p,p'*-DDT<br>. DDE	NR	*Tert 3 vs Tert 1*<br>. *p,p'*-DDT: OR=1.7 [1.0-3.0]	. Non-detectable values: NR<br>. Model selection: NR<br>. Type of controls: benign breast disease (BDD) and non-BBD controls
Burger M et al.<br>2000 [65]<br>Uruguay	. Retrospective<br>. Lag time: NA<br>. n=56 cases<br>. n=25 controls<br>. Follow-up: NA	. Age: mean age 61 y<br>. Menopausal status: NR<br>. Invasive and non-invasive cancers<br>. % Invasive: 95%<br>. % ER+: NR	. Timing: NR<br>. Serum and breast adipose tissue<br>. Method: GC-ECD<br>. Lipid-adjustment: NR	. HCB<br>. α-, β- and γ-HCH<br>. Aldrin<br>. Dieldrin, Endrin<br>. Heptaclor<br>. Heptaclor epoxide<br>. *p,p'*-DDT<br>. *p,p'*-DDE<br>. *o,p'*-DDE<br>. *p,p'*-DDD	None	NR	. Non-detectable values: NR<br>. Model selection: NR<br>. Type of controls: benign breast disease, including non-proliferative (74%), proliferative without atypia (14%) and proliferative with atypia (14%) diseases.

Table S3. Studies of POPs measured in peripheral blood and breast cancer risk *(continued)*

Study reference<br>Publication year<br>Location	Design	Patients and tumors characteristics[*]<br>(mean ± SD)	Exposure assessment	Evaluated POPs	Associated POPs<br>Estimate [95% confidence interval]<br>Without adjustment	 <br>With adjustment[**]	Comments
**Hospital-based case-control studies with hospital-controls**							
Olaya-Contreras P. et al. 1998 [66]<br>Colombia	. Retrospective<br>. Lag time: NA<br>. n=153 cases<br>. n=153 controls<br>. Follow-up: NA	. Age: NR<br>. Post-menopausal: 61%<br>. Invasiveness: NR<br>. % Invasive: NR<br>. % ER+: NR	. Timing: prior to chemotherapy<br>. Serum<br>. Method: GC-ECD<br>. Lipid-adjustment: NR	. DDT<br>. DDE<br>. DDD	*Tert 3 vs Tert 1*<br>. DDE: OR=1.8 [1.1-3.1]	*Tert 3 vs Tert1:*<br>. DDE: OR=2.0 [1.1-3.5]	. Non-detectable values: NR<br>. Model selection: NR<br>. Type of controls: non-cancer patients from another hospital<br>. Results for DDT and DDD not reported
Lopez-Carrillo L et al. 1997 [67]<br>Mexico	. Retrospective<br>. Lag time: NA<br>. n=141 cases<br>. n=141 controls<br>. Follow-up: NA	. Age: NR<br>. Post-menopausal: 50%<br>. Invasiveness: NR<br>. % Invasive: NR<br>. % ER+: NR	. Timing: prior to treatment<br>. Serum<br>. Method: GC-ECD<br>. Lipid-adjusted	. DDE<br>. p'p-DDT<br>. o'p-DDT	None	None	. Non-detectable values: 97% of o'p-DDT; not analysed<br>. Model selection: NR<br>. Type of controls: clinical services except gynecology and oncology
Schecter A et al. 1997 [68]<br>Vietnam	. Retrospective<br>. Lag time: NA<br>. n=21 cases<br>. n=21 controls<br>. Follow-up: NA	. Age: 42.3 ± 6.9 y<br>. Post-menopausal: 14%<br>. Invasive cancers<br>. % Invasive: 100%<br>. % ER+: NR	. Timing: after diagnosis<br>. Serum<br>. Method: GC-ECD<br>. Lipid-adjustment: NR	. *p,p'*-DDT<br>. *p,p'*-DDE<br><br>*(individually and summed)*	None	None	. Non-detectable values: NR<br>. Model selection: change in estimate (10%) ; Method: NR<br>. Type of controls: breast fibrocystic disease
Dewailly E et al. 1994 [69]<br>Canada	. Retrospective<br>. Lag time: NA<br>. n=20 cases<br>. n=17 controls<br>. Follow-up: NA	. Age: mean=54.1 y<br>. Post-menopausal: NR<br>. Invasive cancers<br>. % Invasive: 100%<br>. % ER+: 45%	. Timing: At the time of biopsy<br>. Plasma<br>. Method: GC-ECD<br>. Lipid-adjustment: NR	. 10 PCBs<br>. DDE<br>. HCB<br>. β-HCH<br>. Oxychlordane<br>. *trans*-nonachlor<br>. Mirex	Higher in cases:<br>. HCB<br>. DDE	NR	. Non-detectable values: NR<br>. Model selection: NR<br>. Type of controls: benign breast disease excluding hyperplasia
**Case-Control studies (Unclassified)**							
Ye ZH et al. 2009 [70]<br>China	. Retrospective<br>. Lag time: NA<br>. n=78 cases<br>. n=72 controls<br>. Follow-up: NA	. Age: 40-64 y<br>. Post-menopausal: 55%<br>. Invasive cancers<br>. % Invasive: 100%<br>. % ER+: 66%	. Timing: after diagnosis<br>. Serum<br>. Method: NR<br>. Lipid-adjustment: None	. 7 PCBs	NR	. PCB 153: OR=2.9 [1.4-5.7]<br>. PCB 180: OR = 2.3 [1.2-4.7]	. Non-detectable values: NR<br>. Model selection: NR<br>. Type of controls: NR
**Cross-Sectional studies**							
Sprague BL et al. 2013 [71]<br>USA	. n=264<br>. Women free of cancer<br>. Outcome: breast density	. Age: 60.6 ± 4.4 y<br>. Post-menopausal: 100%	. Timing: at time of the mammogram<br>. Serum<br>. Method: HPLC-MS-MS<br>. Lipid-adjustement: NR	. Phtalates<br>. Parabens<br>. Phenols	NR	. BPA: β=0.191, p=0.01<br>. Mono-ethyl phthalate β=0.034, p=0.01	. Non-detectable values: categorized as "non-detectable"<br>. Model selection: *a priori* variable selection<br>. BPA positively associated with percentage density only not obese women
Diorio C et al. 2013 [72]<br>Canada	. n=106<br>. Women free of cancer<br>. Outcome: breast density	. Age: 58.2 ± 5.6 y<br>. Post-menopausal: 100%	. Timing: at time of the mammogram<br>. Plasma<br>. Method: GC-ECD<br>. Lipid-adjusted	. 24 PCBs<br><br>*(individually and summed in categories)*	None	*Percentage density*<br>. PCB 153: r_s= –0.24 (p=0.03)<br>. PCB 183: r_s= –0.30 (p=0.004)<br>. PCB 196: r_s= –0.22 (p=0.04)<br>. Group 3: rs= –0.22 (p=0.04)<br>*Absolute density*<br>. PCB 183: r_s= –0.24 (p=0.02)	. Non-detectable values: NR<br>. Model selection: *a priori* variable selection

POPs: persistent organic pollutants; [*]at diagnosis; SD: Standard deviation; y: years; NR: Not reported; NA: Not applicable; MS: Mass spectrometry; LD: limit-detection; LC-MS-MS: liquid chromatography–tandem mass spectrometry; GC-MS-MS: gas chromatography-tandem mass spectrometry; HPLC/FD: high-performance liquid chromatography with fluorescence detection; GC-IDMS: gas-chromatography isotope-dilution mass-spectrometry; GC-ECD: gas chromatography with electron Capture Detector; GC-ID-HRMS: gas chromatography-isotope dilution high-resolution mass spectrometry ; HR-GC-ECD: high-resolution gas chromatography with micro-electron capture detection ; HLPC: High-performance liquid chromatography; GC: gas chromatography; PFCs: perfluorinated compounds ; LOD: limit of detection
Prospective/retrospective/cross-sectional: timing of sample in regards to breast cancer diagnosis ; Lag time: time between blood draw and breast cancer diagnosis
** only when all important confounders were considered for adjustment;

Table S4. Studies of POPs measured in breast adipose tissue and breast cancer risk

Study reference<br>Publication year<br>Location	Design	Patients and tumors characteristics*<br>(mean ± SD)	Exposure assessment	Evaluated POPs	Associated POPs<br>Estimate [95% confidence interval]<br>Without adjustment	 <br>With adjustment**	Comments
**Hospital-based case-control studies, hospital controls**							
He Y et al.<br>2018 [73,74]<br>China	. Cross-sectional<br>. n=209 cases<br>. n=165 controls	. Age: 52.0 ± 9.9 y, range [25-80]<br>. Post-menopausal: 56%<br>. Invasive and non-invasive cancers<br>. % Invasive: 95%<br>. % ER+: 68%	. Timing: prior to chemotherapy and radiotherapy<br>. Breast adipose tissue for cases and breast and abdominal adipose tissue for controls<br>. Method: GC-MS<br>. Lipid-adjusted	14 PBDE<br>(17, 28, 47, 66, 71, 85, 99, 100, 138, 153, 154, 183, 190, 209)<br><br>*(individually and summed)*	*Tert 3 vs tert 1*<br>. BDE-28: OR=4.2 [2.5-7.1]<br>. BDE-47: OR=8.7 [4.6-16.3]<br>. BDE-74: OR=0.5 [0.3-0.8]<br>. BDE-99: OR=4.5 (2.6-7.7]<br>. BDE-100: OR=8.6 [4.6-16.2]<br>. BDE-138: OR=3.5 [2.0-5.9]<br>. BDE-153: OR=2.0 [1.2-3.4]<br>. BDE-154: OR=2.4 [1.4-4.2]<br>. BDE-183: OR=0.8 [0.5-1.4]<br>. BDE-190: OR=1.2 [0.7-2.1]<br>. BDE-209: OR=6.9 [3.7-12.8]<br>. ΣPBDEs: OR=2.3 [1.4, 3.9]	*Tert 3 vs tert 1*<br>. BDE-28: OR=2.8 [1.6-4.9]<br>. BDE-47: OR=5.5 [3.0,10.1]<br>. BDE-71: OR=0.4 [0.2-0.7]<br>. BDE-99: OR=3.2 [1.9-5.6]<br>. BDE-100: OR=5.5 [2.9-10.2]<br>. BDE-138: OR=2.4 [1.4-4.2]<br>. BDE-153: OR=1.7 [1.0-3.0]<br>. BDE-154: OR=1.8 [1.1-3.2]<br>. BDE-183: OR=0.7 [0.4-1.3]<br>. BDE-190: OR=1.1 [0.6-1.9]<br>. BDE-209: OR=4.7 [2.5-8.8]<br>. ΣPBDEs: OR=1.8 [1.1-3.1]	. Non-detectable values: NR; low detection rates of BDE-17, BDE-66, BDE-85 (not analysed)<br>. Model selection: variables not balanced between cases and controls and stepwise logistic regression<br>. Type of controls: benign breast disease (n=14) or non-breast-related diseases (n=151, abdominal adipose tissue)
He TT et al.<br>2017 [75]<br>China	. Retrospective<br>. n=56 cases<br>. n=46 controls	. Age: 95% >40 y<br>. Post-menopausal: 55%<br>. Invasive cancers<br>. % Invasive: 100%<br>. % ER+: 79%	. Timing: prior to chemotherapy and radiotherapy<br>. Breast adipose tissue for cases and controls<br>. Method: GC-ECD<br>. Lipid-adjustment: NR	. β-HCH<br>. γ-HCH<br>. PCB 28<br>. PCB 52<br>. PCTA<br>. *p,p'*-DDE	Higher in cases:<br>. PCB 52<br>. *p,p'*-DDE<br>*Incomplete adjustement*<br>. PCB 52: OR=0.7 [0.4-1.0]<br>. *p,p'*-DDE: OR= 0.1 [0.0-0.5]	NR	. Non-detectable values: 95% for PCB 28 (not analysed)<br>. Model selection: NR ; neither breastfeeding nor parity were considered<br>. Type of controls: benign breast disease (adenosis)
Ociepa-Zawal M et al. 2010 [76]<br>Poland	. Retrospective<br>. n=54 cases<br>. n=23 controls	. Age: mean=58.1 y, range [36-87]<br>. Menopausal status: NR<br>. Invasiveness: NR<br>. % Invasive: NR<br>. % ER+: NR	. Timing: at time of surgery<br>. Breast adipose tissue for cases, abdominal adipose tissue for controls<br>. Method: GC-MS<br>. Lipid adjusted (mg/kg fat)	. DDT<br>. DDE<br>. DDD<br>. α-, β- and γ-HCH<br>. HCB<br>. Heptachlor<br>. Heptachlor epoxide<br>. Aldrin<br>. Dieldrin<br>. Endrin	Higher in cases with ER-positive tumors)<br>. β-HCH	NR	. Non-detectable values: NR<br>. Model selection: NR<br>. Type of controls: non-cancer patients of the same clinical department
Hurley S et al.<br>2010 [77]<br>Reynolds P et al.<br>2005 [78]<br>USA	. Retrospective<br>. n=79 cases<br>. n=52 controls	. Age: 57% >50 y<br>. Post-menopausal: 55%<br>. Invasive cancers<br>. % Invasive: 100%<br>. % ER+: NR	. Timing: diagnostic biopsy or surgical treatment<br>. Breast adipose tissue for cases and controls<br>. Method: GC-MS<br>. Lipid adjusted (pg/g of lipid)	. 17 dioxins and furans (PCDD/PCDFs)<br>. 5 PBDEs (BDE 47, 99, 100, 153, 154)<br><br>*(individually and summed in categories of Toxic Equivalents)*	None	None	. Non-detectable values: imputed half the detection limit<br>. Model selection: NR<br>. Type of controls: benign histological changes excluding pre-cancerous lesions
Cassidy RA et al.<br>2005 [79]<br>USA	. Retrospective<br>. n=17 cases<br>. n=17 controls	. Age: median=62 y, range [51-82]<br>. Menopausal status: NR<br>. Invasiveness: NR<br>. % Invasive: NR<br>. % ER+: NR	. Timing: at time of breast biopsy<br>. Breast adipose tissue for cases and controls<br>. Method: GS-ECD<br>. Lipid adjusted (ng/g of lipid)	. Oxychlordane<br>. Heptachlor epoxide<br>. DDE	Higher in cases:<br>. Heptachlor epoxide	. Heptachlor epoxide, age-adjusted median risk of quart 4 =3.2-fold [1.1 to 9.2 fold] that of quart1	. Non-detectable values: NR<br>. Model selection: statistical significance (0.05) ; backward stepping<br>. Type of controls: benign breast diseases including high risk lesions
Siddiqui MKJ et al.<br>2005 [55]<br>India	. Retrospective<br>. n=25 cases<br>. n=25 controls	. Age: 49.7± 19.6 y<br>range [32-82]<br>. Menopausal status: NR<br>. Invasiveness: NR<br>. % Invasive: NR<br>. % ER+: NR	. Timing: at time of surgery<br>. Breast adipose and tumor tissue for cases and controls<br>. Method: GS-MS<br>. Lipid adjusted (ppb)	. α, β, γ and δ-HCH<br>. *p,p'*-DDT<br>. *o,p'*-DDT<br>. *p,p'*-DDE<br>. *p,p'*-DDD	Lower in cases<br>. α-HCH<br>. γ-HCH	NR	. Non-detectable values: NR<br>. Model selection: NR<br>. Type of controls: palpable benign breast lesions

Table S4. Studies of POPs measured in breast adipose tissue and breast cancer risk *(continued)*

Study reference<br>Publication year<br>Location	Design	Patients and tumors characteristics*<br>(mean ± SD)	Exposure assessment	Evaluated POPs	Associated POPs<br>Estimate [95% confidence interval]<br>Without adjustment	 <br>With adjustment**	Comments
**Hospital-based case-control studies, hospital controls**							
Waliszewski SM et al. 2005 [80]<br>Mexico	. Retrospective<br>. n=127 cases<br>. n=254 controls	. Age: mean=49 y<br>. Menopausal status: NR<br>. Invasive cancers<br>. % Invasive: 100%<br>. % ER+: NR	. Timing: at time of surgery<br>. Breast adipose tissue for cases and benign breast controls ; abdominal adipose tissue for autopsy controls<br>. Method: GS-ECD<br>. Lipid adjusted (mg/kg fat)	. HCB<br>. β-HCH<br>. *p,p'*-DDE<br>. *o,p'*DDT<br>. *p,p'*-DDT	*Cases compared to autopsy controls; grouping of exposure not reported:*<br>. HCB: RR=2.0 [1.9-2.1]<br>. β-HCH: RR=1.6 [1.5-1.6]<br>. pp'-DDE: RR=1.2 [1.1-1.2]<br>. op'-DDT: RR=2.3 [2.2-2.4]<br>. pp'-DDT: RR=1.3 [1.3-1.4]<br>. Total DDT: RR=1.2 [1.2-1.2]	NR	. Non-detectable values: NR<br>. Model selection: NR<br>. Type of controls: benign breast changes and autopsies from car accidents<br>. All analytes higher in benign breast controls than in cases<br>. Stronger associations when benign breast controls compared to autopsy controls
McCready D et al. 2004 [81]<br>Canada	. Retrospective<br>. n=70 cases<br>. n=69 controls	. Age: 52.5 ± 10.0 y<br>. Post-menopausal: 49%<br>. Invasive cancers<br>. % Invasive: 100%<br>. % ER+: NR	. Timing: at time of biopsy<br>. Non-cancerous breast tissue for cases and controls<br>. Method: GC-ECD<br>. Lipid adjusted (µg/kg lipid)	. 14 PCBs<br>. Total PCBs<br>. *p,p'*-DDT<br>. *p,p'*-DDE<br>. *cis*-nonachlor<br>. *trans*-nonachlor<br>. oxychlordane<br>. HCB<br>. Mirex<br>. β-HCH<br>. α- and γ-chlordane	Higher in cases<br>. PCB 99<br>. PCB 118<br>. *p,p'*-DDE<br>. *p,p'*-DDT<br>. HCB	*Above vs below median:*<br>. PCB 105: OR=2.5 [1.0-6.1]<br>. *p,p'*-DDE: OR=2.5 [1.1-5.7]<br><br>*Homo wt/hetero GSTT1, above vs below median:*<br>. *p,p'*-DDT: OR=3.0 [1.1-8.1]	. Non-detectable values: >30% for PCBs 28, 52, 101, 128, α-and γ-chlordane ; not analysed<br>. Model selection: *a priori* variable selection<br>. Type of controls: biopsies negative for malignancy<br>. Possible effect modification by GSTM1, GSTT1, CYP1A1-M1
Ibarluzea JM et al. 2004 [82]<br>Spain	. Retrospective<br>. n=198 cases<br>. n=260 controls	. Age: mean= 54.8 y<br>. Post-menopausal: 56%<br>. Invasive and non-invasive cancer<br>. % Invasive: 95.5%<br>. % ER+: NR	. Timing: before chemotherapy or radiotherapy<br>. Breast adipose tissue for cases and abdominal adipose tissue for controls<br>. Method: GC-ECD<br>. Lipid adjusted (ng/g of lipid)	. 15 organochlorines<br>. Total effective xenoestrogen burden (HPLC fractions α and β)	None	*Above vs below detection limit:*<br>. Aldrin: OR =1.6 [1.0–2.4]<br><br>*Post-menopausal women, above vs below detection limit:*<br>. Lindane: OR=1.8 [1.0–3.0]<br><br>*Women with BMI below median, quart 4 vs quart 1*<br>. TEXB-α: OR=2.4 [1.0-5.8]	. Non-detectable values: imputed half the detection limit value ; >40% detection only for aldrin, lindane, and endosulfan-ether<br>. Model selection: NR<br>. Type of controls: non-cancer-related surgery<br>. Stronger associations in post-menopausal women
Rusiecki JA et al 2004 [59]<br>USA	. Retrospective<br>. n=244 cases<br>. n=186 controls	. Age: 69% ≥ 50 y<br>. Post-menopausal: 65%<br>. Invasiveness: NR<br>. % Invasive: NR<br>. % ER+: 50%	. Timing: at time of surgery<br>. Breast adipose tissue for cases and controls<br>. Method: GC<br>. Lipid adjusted (ng/g)	. PCBs: 74, 118, 138, 153, 156, 170, 180, 183, 187<br><br>*(individually and summed)*	None	None	. Non-detectable values: <30%; treatment in analyses: NR<br>. Model selection: NR<br>. Type of controls: benign breast disease excluding atypical hyperplasia, or normal breast tissue<br>. All analyses stratified by stratified by joint ER/PR status
Lucena RA et al. 2001 [83]<br>Spain	. Retrospective<br>. n=69 cases<br>. n=65 controls	. Age: NR<br>. Menopausal status: NR<br>. Invasiveness: NR<br>. % Invasive: NR<br>. % ER+: NR	. Timing: at time of surgery<br>. Breast fat for cases and controls<br>. Method: GC<br>. Lipid adjustment: NR	. PCBs: 28, 52, 101, 118, 138, 153, 170, 180,183, 187, 188	NR	. PCB 28<br>OR=9.6 [3.8-24.4]	. Non-detectable values: NR<br>. Model selection: NR<br>. Type of controls: benign breast lumps<br>. PCBs 138, 153, 170, 180 and 187 identified in all the studied subjects<br>. Exposure grouping: NR

Table S4. Studies of POPs measured in breast adipose tissue and breast cancer risk *(continued)*

Study reference<br>Publication year<br>Location	Design	Patients and tumors characteristics*<br>(mean ± SD)	Exposure assessment	Evaluated POPs	Associated POPs<br>Estimate [95% confidence interval]<br>Without adjustment	 <br>With adjustment**	Comments
**Hospital-based case-control studies, hospital controls**							
Charles MJ et al.<br>2001 [84]<br>USA	. Retrospective<br>. n=44 cases<br>. n=21 controls	. Age: NR<br>. Menopausal status: NR<br>. Invasiveness: NR<br>. % Invasive: NR<br>. % ER+: NR	. Timing: at time of surgery<br>. Cancerous tissue for cases, noncancerous breast tissue for controls<br>. Method: GC-ECD<br>. Lipid adjusted	. 18 PCBs<br>. Total PCBs<br>. *o,p'*-DDE<br>. *p,p'*-DDE<br>. *o,p'*-DDT<br>. *p,p'*-DDT	Higher in cancerous tissues<br>. *p,p'*-DDE	NR	. Non-detectable values: imputed one-half the lowest value<br>. Model selection: NR<br>. Type of controls: benign (not further specified)
Woolcott CG et al.<br>2001 [85] and<br>Aronson KJ et al.<br>2000 [86]<br>Canada	. Retrospective<br>. n=217 cases<br>. n=213 controls	. Age: mean=58 y<br>. Menopausal status: NR<br>. Invasive and non-invasive cancers<br>. % Invasive: 78%<br>. % ER+: 75%	. Timing: at time of biopsy<br>. Benign breast tissue of cases and controls<br>. Method: GC-ECD<br>. Lipid adjusted	. 14 PCBs<br>. *p,p'*-DDT<br>. *p,p'*-DDE<br>. *cis*-nonachlor<br>. *trans*-nonachlor<br>. Oxychlordane<br>. α- and γ-chlordane<br>. HCB<br>. β-HCH<br>. Mirex	None	Quart 4 *vs* quart 1 :<br>. PCB 105: OR=3.2 [1.5-6.7]<br>. PCB 118: OR=2.3 [1.1-4.8]<br><br>*ER-negative tumors, tert 3 vs tert 1:*<br>. *p,p'*-DDE: OR=2,4 [1.0-5.4]	. Non-detectable values: >30% for PCB 28,52, 101, 128 (not analysed); for others: excluded<br>. Model selection: forward selection (p-value <0.3) then backward selection (change in estimate of >10%) ;<br>. Type of controls: breast biopsy negative for malignancy
Zheng T et al.<br>2000 [87,88],<br>*1999 [89-91] and Holford TR 2000 [92]*<br>USA	. Retrospective<br>. n=304 cases<br>. n=186 controls	. Age: mean=56.3 y<br>. Post-menopausal: 71%<br>. Invasive and non-invasive cancers<br>. % Invasive: 76%<br>. % ER+: 52%	. Timing: at time of biopsy<br>. Breast adipose tissue for cases and controls<br>. Method: GC<br>. Lipid adjusted (ppb)	. 9 PCBs<br>. *trans*-nonachlor<br>. Oxychlordane<br>. β-HCH<br>. DDT<br>. DDE<br>. HCB<br>*(individually and summed PCBs)*	None	. PCB 183: OR=1.8 [1.1–3.0]<br>. PCB 153: OR=0.9 [0.8–1.0]<br>. PCB 156: OR=0.8 [0.6-1.0]<br>. PCB 180: OR=1.1 [1.0–1.3]<br>. Oxychlordane, OR=0.7 [0.4-1.3]<br>. *trans*-nonachlor OR=1.1 [0.6-1.9]<br>*(quart 4 vs quart 1)*	. Non-detectable values: NR<br>. Model selection: *a priori* variable selection<br>. Type of controls: benign breast (excluding atypical hyperplasia) or normal breast tissue
Stellman SD et al.<br>2000 [93]<br>USA	. Retrospective<br>. n=232 cases<br>. n=323 controls	. Age: 66% >50 y<br>. Post-menopausal: 59%<br>. Invasive and non-invasive cancers<br>. % Invasive: 86%<br>. % ER+: 56%	. Timing: at time of biopsy<br>. Breast adipose tissue for cases and breast controls ; abdominal adipose tissue for non-breast controls<br>. Method: GC-ECD<br>. Lipid-adjusted (ng/g)	. 14 PCBs<br>. *p,p'*-DDE<br>. *p,p'*-DDT<br>. *o,p'*-DDD<br>. oxychlordane<br>. *trans*-nonachlor<br>. β-HCH<br>. HCB<br>*(individually and summed)*	None	None	. Non-detectable values: NR<br>. Model selection: NR<br>. Type of controls: benign breast diseases and non-breast-related
Bagga D et al.<br>2000 [94]<br>USA	. Retrospective<br>. n=73 cases<br>. n=73 controls	. Age: 57.5 ± 31.1 y<br>. Post-menopausal: NR<br>. Invasiveness: NR<br>. % Invasive: NR<br>. % ER+: NR	. Timing: at the time of biopsy<br>. breast adipose tissue for cases and controls<br>. Method: GC-ECD<br>. Lipid-adjusted (wet-weight basis and lipid basis)	DDT and its metabolites<br><br>*(individually and summed)*	Higher in cases<br>. DDE<br>. sum of DDTs	None	. Non-detectable values: NR<br>. Model selection: a priori variables selection and/or associated with outcome in univariate analyses<br>. Type of controls: reduction mammoplasty for mastomegaly
Burger M et al.<br>2000 [65]<br>Uruguay	. Retrospective<br>. Lag time: NA<br>. n=56 cases<br>. n=25 controls<br>. Follow-up: NA	. Age: mean age 61 y<br>. Menopausal status: NR<br>. Invasive and non-invasive cancers<br>. % Invasive: 95%<br>. % ER+: NR	. Timing: NR<br>. Serum and breast adipose tissue<br>. Method: GC-ECD<br>. Lipid-adjustment: NR	. HCB<br>. α-, β- and γ-HCH<br>. Aldrin<br>. Dieldrin<br>. Endrin<br>. Heptaclor<br>. Heptaclor epoxide<br>. *p,p'*-DDT<br>. *p,p'*-DDE<br>. *o,p'*-DDE<br>. *p,p'*-DDD	β-HCH higher in cases	NR	. Non-detectable values: NR<br>. Model selection: NR<br>. Type of controls: benign breast disease, including non-proliferative (74%), proliferative without atypia (14%) and proliferative with atypia (14%) diseases.

Table S4. Studies of POPs measured in breast adipose tissue and breast cancer risk *(continued)*

Study reference<br>Publication year<br>Location	Design	Patients and tumors characteristics*<br>(mean ± SD)	Exposure assessment	Evaluated POPs	Associated POPs<br>Estimate [95% confidence interval]<br>Without adjustment	 <br>With adjustment**	Comments
**Hospital-based case-control studies, hospital controls**							
Liljegren G et al.<br>1998 [95]<br>Sweden	. Retrospective<br>. n=43 cases<br>. n=35 controls	. Age: mean=57.7 y<br>. Post-menopausal: 74%<br>. Invasive cancers<br>. % Invasive: 100%<br>. % ER+: 74%	. Timing: during surgery<br>. Breast adipose tissue for cases and controls<br>. Method: GC-MS<br>. Lipid-adjusted	. Total of 39 PCBs<br>. DDE<br>. HCB<br><br>*(individually for PCBs 77, 126 and 169)*	. PCB 77<br>. PCB 126<br>. PCB 169<br>. HCB	*Postmenopausal women with ER+ tumours*<br>*Above vs below 4.5 pg/g lipid*<br>. PCB 77: OR=33 [1.8-588]<br>*Above vs below 40 ng/g lipid*<br>. HCB: OR= 7.1 [1.1-45]	. Non-detectable values: NR<br>. Model selection: NR<br>. Type of controls: benign breast disease
Güttes S et al.<br>1998 [96]<br>Germany	. Retrospective<br>. n=45 cases<br>. n=20 controls	. Age: median=60 y<br>. Menopausal status: NR<br>. Invasive cancers<br>. % Invasive: 100%<br>. % ER+: NR	. Timing: at the time of surgery<br>. Tumor and surrounding area for cases, diseased tissue for controls<br>. Method: GS-MS<br>. Lipid-adjusted (µg/kg fat)	. 13 PCBs<br>. α-, β and γ-HCH<br>. HCB<br>. *p,p'*-DDT<br>. *p,p'*-DDE<br>. *p,p'*-DDD	*Adjusted for age only, h*igher in cases:<br>. *p,p'*-DDE<br>. PCB 118	NR	. Non-detectable values: <30% for PCB 149, 105 and lindane (not analysed)<br>. Model selection: NR<br>. Type of controls: benign breast disease
Hardell L et al.<br>1996 [97]<br>Sweden	. Retrospective<br>. n=22 cases<br>. n=19 controls	. Age: mean=63 y, range [42-72]<br>. Post-menopausal: 82%<br>. Invasive cancers<br>. % Invasive: 100%<br>. % ER+: 64%	. Timing: at time of surgery<br>. Breast tissue for cases and controls<br>. Method: GC-MS<br>. Lipid-adjusted (pg/g lipid)	. Dioxins (PCDDs)<br>. Furans (PCDFs)	Higher in cases:<br>. OCDD<br>*(post-menopausal)*	None	. Non-detectable values: NR<br>. Model selection: NR<br>. Type of controls: benign breast lesion
Dewailly E et al<br>1994 [69]<br>Canada	. Retrospective<br>. n=20 cases<br>. n=17 controls	. Age: mean=54.1 y<br>. Post-menopausal: NR<br>. Invasive cancers<br>. % Invasive: 100%<br>. % ER+: 45%	. Timing: At the time of biopsy<br>. Breast adipose tissue for cases and controls<br>. Method: GC-ECD<br>. Lipid-adjusted (µg/kg)	. 10 PCBs<br>. DDE<br>. HCB<br>. β-HCH<br>. Oxychlordane<br>. *trans*-nonachlor<br>. Mirex	*Higher in cases:*<br>. HCB<br>*Higher in ER-positive cases vs controls:*<br>. DDE<br>. PCB 99<br>*Higher in controls vs ER-negative cases*:<br>. PCB 118	*Mean+SD of controls vs mean-SD of control, adjusted for age only:*<br>. DDE: OR=8.9 [NR]	. Non-detectable values: NR<br>. Model selection: NR<br>. Type of controls: benign breast disease excluding hyperplasia
Djordjevic MV et al.<br>1994 [98]<br>USA	. Retrospective<br>. n=5 cases<br>. n=5 controls	. Age: mean=58.8<br>. Post-menopausal: NR<br>. Invasiveness: NR<br>. % Invasive: NR<br>. % ER+: 3/5 cases	. Timing: at time of surgery<br>. Adipose tissue adjacent to tumor for cases; NR for controls<br>. Method: GC-ECD<br>. Lipid-adjusted (ng/g net weight)	. Total PCBs<br>. HCB<br>. α-, β- and γ-HCH<br>. Heptachlor epoxide<br>. *trans*-nonachlor<br>. Oxychlordane<br>. Dieldrin<br>. Endrin<br>. DDT<br>. DDE	Higher in cases:<br>. HCB<br>. Oxychlordane<br>. p-p'-DDE<br>. p-p'-DDT<br>. Total PCBs	NR	. Non-detectable values: NR<br>. Model selection: NR<br>. Type of controls: hospital controls<br>. Pilot study of Stellman et al.
Falck F et al.<br>1992 [99]<br>USA	. Retrospective<br>. n=20 cases<br>. n=20 controls	. Age: 63 ± 13 y range [36-86]<br>. Post-menopausal: NR<br>. Invasive and non-invasive cancers<br>. % Invasive: 90%<br>. % ER+: NR	. Timing: at time of surgery<br>. Breast adipose tissue for cases and controls<br>. Method: chromatography<br>. Lipid-adjusted	. PCBs<br>. HCB<br>. γ-HCH<br>. Heptachlor epoxide<br>. Oxychlordane<br>. *trans*-nonachlor<br>. *p,p'*-DDE<br>. *p,p'*-DDT	Higher in cases<br>. DDE<br>. DDT<br>. PCBs	NR	. Non-detectable values: NR<br>. Model selection: NR<br>. Type of controls: benign diseases excluding atypia<br>. Pilot study

Table S4. Studies of POPs measured In breast adipose tissue and breast cancer risk *(continued)*

Study reference<br>Publication year<br>Location	Design	Patients and tumors characteristics* (mean ± SD)	Exposure assessment	Evaluated POPs	Associated POPs Estimate [95% confidence interval]<br>Without adjustment	 With adjustment**	Comments
**Hospital-based case-control studies, hospital controls**							
Mussalo-Rauhamaa H et al. 1990 [100]<br>Finland	. Retrospective<br>. n=44 cases<br>. n=33 controls	. Age: 58 ± 13<br>. Menopausal status: NR<br>. Invasiveness: NR<br>. % Invasive: NR<br>. % ER+: NR	. Timing: NR<br>. Breast-tissue for cases and controls ; adjacent to tumors for cases<br>. Method: GC-MS<br>. Lipid-adjusted (µg/kg fat)	. PCBs<br>. 18 other organochlorines<br>. Polycyclic aromatic hydro- carbons (PAH)	Higher in cases:<br>. β-HCH	. β-HCH, > *vs* < to 0.1 mg/kg: OR=10.5 [2.0-55.3]	. Non-detectable values: NR<br>. Model selection: stepwise logistic regression<br>. Type of controls: routine post-mortem examinations
Unger M et al. 1984 [101]<br>Denmark	. Retrospective<br>. n=18 cases / n=25 controls (autopsies)<br>. n=14 cases / n=21 controls (surgery)	. Age:<br>mean=61 y, range [43-82] (autopsy)<br>mean=40 y, range [25-54] (surgery)<br>. Post-menopausal: NR<br>. Invasiveness: NR<br>. % Invasive: NR<br>. % ER+: NR	. Timing: at autopsy or at time of surgery<br>.Breast adipose tissue for cases and controls<br>. Method: GC-ECD<br>. Lipid-adjusted (ppm)	. PCB<br>. DDE	None	None	. Non-detectable values: NR<br>. Model selection: NR<br>. Type of controls: deceased women without cancer and living women with benign disorders of the breast
Wassermann M et al. 1976 [102]<br>Brazil	. Retrospective<br>. n=9 cases<br>. n=5 controls	. Age: NR<br>. Menopausal status: NR<br>. Invasiveness: NR<br>. % Invasive: NR<br>. % ER+: NR	. Timing: NR<br>. Malignant tissue, adjacent normal glandular and adipose tissue for cases ; mammary gland and adjacent adipose tissue for controls<br>. Method: GC-ECD<br>. Lipid-adjusted	. PCBs<br>. *p,p'*-DDE<br>. *p,p'*-DDT<br>. *p,p'*-DDD<br>. *o,p'*-DDT<br>. *o,p'*-DDE<br>. *o,p'*-DDD<br>. γ-HCH<br>. Dieldrin<br>*(individually and sum of PCBs)*	Higher in tumors of cases:<br>. *o,p'*-DDT<br>. *o,p'*-DDE<br>. *o,p'*-DDD<br>. γ-HCH<br>. Dieldrin<br>. Total PCBs<br><br>Higher in adipose tissue of cases:<br>. Heptachlor epoxide	NR	. Non-detectable values: NR<br>. Model selection: NR<br>. Type of controls: autopsies from car accidents<br>. Higher analytes in malignant tissues compared to adjacent normal and adipose tissue of cases

POPs: persistent organic pollutants; *at diagnosis; SD: Standard deviation; y: years; NR: Not reported; NA: Not applicable; MS: Mass spectrometry; LD: limit-detection; LC-MS-MS: liquid chromatography–tandem mass spectrometry; GC-MS-MS: gas chromatography-tandem mass spectrometry; GC-IDMS: gas-chromatography isotope-dilution mass-spectrometry; GC-ECD: gas chromatography with electron Capture Detector; GC-ID-HRMS: gas chromatography-isotope dilution high-resolution mass spectrometry ; HR-GC-ECD: high-resolution gas chromatography with microelectron capture detection ; HLPC: High-performance liquid chromatography; PFCs: perfluorinated compounds

Prospective/retrospective/cross-sectional: timing of sample in regards to breast cancer diagnosis ; Lag time: time between blood draw and breast cancer diagnosis;

** only when all important confounders were considered for adjustment.

Table S5. Studies of POPs measured in adipose tissue other than breast and breast cancer risk

Study reference<br>Publication year<br>Location	Design	Patients and tumors characteristics*<br>(mean ± SD)	Exposure assessment	Evaluated POPs	Associated POPs<br>Estimate [95% confidence interval]<br>Without adjustment	 <br>With adjustment**	Comments
**Cohort-nested case-control studies with incidence density (risk-set) sampling**							
Brauner EV et al. 2014 [103]<br>Raaschou-Nielsen O et al. 2005 [104]<br>Denmark	. Prospective<br>. Lag time: not considered<br>. n=409 cases<br>. n=409 controls<br>. Follow-up: median=4.8 y	. Age: 57.5 ± 4.0<br>. Post-menopausal: 100%<br>. Invasiveness: NR<br>. % Invasive: NR<br>. % ER+: 77.5%	. Timing: not considered<br>. Buttock adipose tissue<br>. Method: GC-MS<br>. Lipid-adjusted (µg/kg lipids)	. 18 PCBs<br>. 14 other organochlorines<br><br>*(individually and summed PCBs)*	NR	All tumors, *quart 4 vs quart 1*<br>. HCB: OR =0.5 [0.3-0.9]<br>. β-HCH: OR= 0.5 [0.3-0.9]<br>. Oxychlordane: OR=0.5 [0.3-0.9]<br><br>ER-negative, *quart 4 vs quart 1*<br>. HCB: OR=0.2 [0.0-0.6]<br>. β-HCH: OR=0.2 [0.1-0.8]<br>. *p,p'*-DDE: OR= 0.1 [0.0-0.5]<br>. PCB 118: OR=0.2 [0.0-0.8]<br>. PCB 138: OR=0.3 [0.1-0.9]<br>. PCB 153: OR=0.3 [0.1-0.9]<br>. Total PCBs: OR=0.3 [0.1-0.9]<br>. *trans*-Nonachlor: OR=0.2 [0.1-0.9]	. Non-detectable values: >50% of samples for 8 individual PCBs (not analysed); contributed zero to the PCB sum ; excluded for other organochlorines<br>. Model selection: NR
**Hospital-based case-control studies, community controls**							
van 't Veer P et al. 1997 [105]<br>Europe	. Retrospective<br>. n=265 cases<br>. n=341 controls	. Age: 62.3 ± 6.0<br>. Menopausal status: NR<br>. Invasiveness: NR<br>. % Invasive: NR<br>. % ER+: NR	. Timing: after hospital admission<br>. Subcutaneous fat of the buttocks<br>. Method: GC-ECD<br>. Lipid-adjusted (µg/g of fatty acids)	DDE	NR	. DDE, quart 4 *vs* quart 1 OR=0.5 [0.3 – 1.0]<br>. DDE, continuous OR=0.8 [0.7-1.0]	. Non-detectable values: NR<br>. Model selection: NR

POPs: persistent organic pollutants; *at diagnosis; SD: Standard deviation; y: years; NR: Not reported; NA: Not applicable; MS: Mass spectrometry; LD: limit-detection; LC-MS-MS: liquid chromatography–tandem mass spectrometry; GC-MS-MS: gas chromatography-tandem mass spectrometry; GC-IDMS: gas-chromatography isotope-dilution mass-spectrometry; GC-ECD: gas chromatography with electron Capture Detector; GC-ID-HRMS: gas chromatography-isotope dilution high-resolution mass spectrometry ; HR-GC-ECD: high-resolution gas chromatography with microelectron capture detection ; HLPC: High-performance liquid chromatography; PFCs: perfluorinated compounds
Prospective/retrospective/cross-sectional: timing of sample in regards to breast cancer diagnosis ; Lag time: time between blood draw and breast cancer diagnosis;
** only when all important confounders were considered for adjustment.

Table S6. Studies of POPs measured in peripheral blood and mortality among breast cancer patients

Study reference<br>Publication year<br>Location	Design	Patients and tumors characteristics*<br>(mean ± SD)	Exposure assessment	Evaluated POPs	Associated POPs<br>Estimate [95% confidence interval]<br>Without adjustment	 <br>With adjustment**	Comments
Parada JH et al.<br>2016 [106,107]<br>USA	. Prospective cohort study<br>. n=633<br>. Follow-up: median=14.7y	. Age: 58 ± 12 y, range [29-89]<br>. Post-menopausal: 66%<br>. Invasive and non-invasive cancers<br>. % Invasive: 71%<br>. % ER+: 78%	. Timing: 77% prior to chemotherapy<br>. Serum<br>. Method: GC-ECD<br>. Lipid-adjusted	. 29 PCBs<br>. *p,p'*-DDT<br>. *p,p'*-DDE<br>. Chlordane *(oxychlordane + trans-nonachlor)*<br><br>*(individually and sum of PCBs)*	*All-cause mortality*<br>5-year<br>. *p,p'*-DDT<br>15-year<br>. Chlordane<br><br>*Breast cancer mortality*<br>5-year<br>. *p,p'*-DDT<br><br>*(Not reported for PCBs)*	*All-cause mortality*<br>5-year<br>*. p,p'-DDT: HR=2.2[1.0, 4.7]*<br>*. PCB 174: HR=2.2 [1.1-4.]*<br>*. PCB 177: HR=2.1 [1.1-4.3]*<br>15-year<br>. *p,p'*-DDE: HR=0.7 [0.4, 1.0]<br><br>*Breast cancer mortality*<br>5-year<br>. *p,p'*-DDT: HR=2.7 [1.0-7.1]<br>. PCB 174: HR=3.2 [1.2-8.1]<br>15- year<br>. PCB 174: HR=1.9 [1.1-3.4]<br><br>*(All HR for tert 3 vs tert 1)*	. Non-detectable values: imputed detection limit value divided by square 2<br>. Model selection: *a priori* variable selection<br>. Treatment received and prognostic factors not considered for adjustment<br>. Stronger associations when restricted to invasive breast cancers and post-menopausal women<br>. Stronger associations in the strata of women with BMI<25 kg/mg2
Hoyer AP et al.<br>2001 [23,24]<br>Denmark	. Prospective cohort study<br>. n= 161<br>. Follow-up: median=7.2 y	. Age: NR<br>. Post-menopausal: NR<br>. Invasiveness: NR<br>. % Invasive: NR<br>. % ER+: 72%	. Timing: before diagnosis<br>. Serum<br>. Method: GC-ECD<br>. Lipid-adjusted	. Total of 28 PCBs<br>. HCB<br>. Dieldrin<br>. *p,p'*-DDE<br>. *p,p'*-DDT<br>. Total DDT	NR	*All-cause mortality*<br>*ER-positive tumors; quart 4 vs quart 1:*<br>. Total PCBs: HR= 2.5 [1.1-5.7]<br><br>*Tumor wild-type for p53, for each 1 ng/ml increase:*<br>. Dieldrin: HR=3.5 [NR]	. Non-detectable values: NR<br>. Model selection: statistical significance (0.05); Backward step-wise procedures;<br>. Treatment received not considered for adjustment<br>. All analyses stratified by ER status (no global analyses)
Hoyer AP et al.<br>2000 [16]<br>Denmark	. Prospective cohort study<br>. n= 195<br>. Follow-up: median=79 to 86 months	. Age: mean=65.6 y<br>. Post-menopausal: NR<br>. Invasiveness: NR<br>. % Invasive: NR<br>. % ER+: NR	. Timing: before diagnosis<br>. Serum<br>. Method: GC-ECD<br>. Lipid-adjusted	. Total DDTs<br>. Total of 27 PCBs<br>. β-HCH<br>. HCB<br>. Dieldrin<br>. *p,p'*-DDT<br>. *p,p'*-DDE<br>. *o,p'*-DDT<br>. *o,p'*-DDE<br>. *p,p'*-DDD<br><br>*(individually and sum of DDTs)*	*All-cause mortality*<br>. Dieldrin: HR= 2.4 [1.2–4.9]<br><br>*Breast cancer recurrence and/or death*<br>. Dieldrin: HR= 3.3[1.4-7.6]<br><br>*(All HR for quart 4 vs quart 1)*	*All-cause mortality*<br>. Dieldrin: HR= 2.7 [1.5–4.8]<br><br>*Breast cancer recurrence and/or death*<br>. Dieldrin: HR= 5.8 [1.6-20.5]<br><br>*(All HR for quart 4 vs quart 1)*	. Non-detectable values: NR<br>. Model selection: statistical significance (0.05); method: NR<br>. Treatment received and prognostic factors not considered for adjustment<br>. Stronger associations for higher grade, lymph nodal involvement and advanced stage

POPs: persistent organic pollutants; *at diagnosis; SD: Standard deviation; y: years; NR: Not reported; NA: Not applicable; MS: Mass spectrometry; LD: limit-detection; LC-MS-MS: liquid chromatography–tandem mass spectrometry; GC-MS-MS: gas chromatography-tandem mass spectrometry; GC-IDMS: gas-chromatography isotope-dilution mass-spectrometry; GC-ECD: gas chromatography with electron Capture Detector; GC-ID-HRMS: gas chromatography-isotope dilution high-resolution mass spectrometry ; HR-GC-ECD: high-resolution gas chromatography with microelectron capture detection ; HLPC: High-performance liquid chromatography; PFCs: perfluorinated compounds
Prospective/retrospective/cross-sectional: timing of sample in regards to breast cancer diagnosis ; Lag time: time between blood draw and breast cancer diagnosis ; in bold : compounds actually analysed;
** only when all important confounders were considered for adjustment.

Table S7. Studies of POPs measured in adipose tissue and mortality among breast cancer patients

Study reference<br>Publication year<br>Location	Design	Patients and tumors characteristics*<br>(mean ± SD)	Exposure assessment	Evaluated POPs	Associated POPs<br>Estimate [95% confidence interval]<br>Without adjustment	 <br>With adjustment**	Comments
**Breast adipose tissue**							
Muscat JE et al.<br>2003 [108]<br>USA	. Cohort study<br>. Lag time: NA<br>. n=224<br>. Follow-up: 5 years	. Age: 67% >50 y<br>. Post-menopausal: NR<br>. Invasive and non-invasive cancers<br>. % Invasive: 86%<br>. % ER+: NR	. Timing: at surgery<br>. Breast adipose tissue<br>. Method: GC-ECD<br>. Lipid-adjusted (ng/g)	. DDT<br>. DDD<br>. *p,p'*-DDE<br>. *trans*-nonachlor<br>. Oxychlordane<br>. β- HCH<br>. HCB<br>. 14 PCBs<br><br>*(individually and summed)*	NR	*Breast cancer recurrence, tert 3 vs tert 1*<br>. PCB 118: HR=4.0 [1.3-4.9]<br>. PCB 153: HR=2.6 [1.0-7.1]<br>. PCB 167: HR=3.1 [1.0–9.3]<br>. Total PCBs: HR=2.9 [1.0–8.2]	. Non-detectable values: imputed a zero value<br>. Model selection: NR
**Adipose tissue other than breast**							
Roswall M et al.<br>2018 [109]<br>Raaschou-Nielsen<br>2005 [104]<br>Denmark	. Cohort study<br>. Lag time: NA<br>. n=399<br>. Follow-up: 16.1 years	. Age: median=60 y<br>. Post-menopausal: 100%<br>. Invasiveness: NR<br>. % Invasive: NR<br>. % ER+: 77.5%	. Timing: median of 4.8 years before diagnosis<br>. Buttock adipose tissue<br>. Method: GC-MS<br>. Lipid-adjusted (µg/kg lipids)	. Total of 18 PCBs<br>. *p,p'*-DDE<br><br>*(individually and summed PCBs)*	*All-cause mortality*<br>. PCB 118: HR=0.8 [0.6-1.0]<br>. PCB 138: HR=0.8 [0.6-1.0]<br>. PCB 183: HR=0.7 [0.5-0.9]<br>. *p,p'*-DDT: HR=0.7 [0.5-1.0]<br><br>*Breast-cancer specific mortality*<br>NR	*All-cause mortality*<br>. PCB 118: HR=0.7 [0.5-1.0]<br>. PCB 138: HR=0.8 [0.6-1.0]<br>. PCB 183: HR=0.7 [0.5-0.9]<br>. *p,p'*-DDT: HR=0.7 [0.5-1.0]<br><br>*Breast-cancer specific mortality*<br><br>Negative associations:<br>. PCBs: 187, 201, 118, 156, 138, 170, 99, 153, 180, 183, total PCBs<br>.Organochlorines: *p,p'*-DDE, *p,p'*-DDT, *cis*-nonachlor, *trans*-nonachlor, HCB<br><br>Positive associations:<br>. Dieldrin: HR=1.3 [1.1-1.5]	. Non-detectable values: >50% of samples for 8 individual PCBs (not analysed); contributed zero to the PCB sum ; excluded for other organochlorines<br>. Model selection: NR
Cocco P et al.<br>2000 [110]<br>USA	. Ecologic correlation study<br>. Lag time: 7 years<br>. n= NA<br>. Follow-up: NA	. Age: NA<br>. Post-menopausal: NA<br>. Invasiveness: NR<br>. % Invasive: NR<br>. % ER+: NR	. Timing: NA<br>. Subcutaneous adipose tissue<br>. Method: NR<br>. Lipid-adjustment: NR	DDE	Inverse correlation with breast cancer mortality<br>. *White women:* r= -0.73<br>. *African-American*: r= -0.50	*White women:*<br>Inverse correlation with breast cancer mortality, β= -0.947	. Non-detectable values: NA<br>. Model selection: covariates correlated with cancer sites

POPs: persistent organic pollutants; *at diagnosis; SD: Standard deviation; y: years; NR: Not reported; NA: Not applicable; MS: Mass spectrometry; LD: limit-detection; LC-MS-MS: liquid chromatography–tandem mass spectrometry; GC-MS-MS: gas chromatography-tandem mass spectrometry; GC-IDMS: gas-chromatography isotope-dilution mass-spectrometry; GC-ECD: gas chromatography with electron Capture Detector; GC-ID-HRMS: gas chromatography-isotope dilution high-resolution mass spectrometry ; HR-GC-ECD: high-resolution gas chromatography with microelectron capture detection ; HLPC: High-performance liquid chromatography; PFCs: perfluorinated compounds

Prospective/retrospective/cross-sectional: timing of sample in regards to breast cancer diagnosis ; Lag time: time between blood draw and breast cancer diagnosis ; in bold : compounds actually analysed;

** only when all important confounders were considered for adjustment.

Table S8. Studies of POPs and breast cancer prognostic factors

Study reference<br>Publication year<br>Location	Design	Patients and tumors characteristics*<br>(mean ± SD)	Exposure assessment	Evaluated POPs	Associated POPs<br>Estimate [95% confidence interval]<br>Without adjustment	 <br>With adjustment**	Comments
**Peripheral blood**							
Arrebola JP et al.<br>2016 [111]<br>Spain	. Cross-sectional study<br>. n=103<br>. Prognostic factors: ER, PR, HER2, E-Cadherin, Ki-67, p53	. Age: 53.6 ± 11.8 y<br>. Post-menopausal: 41%<br>. Invasive cancers<br>. % Invasive: 100%<br>. % ER+: 86%	. Timing: at time of surgery<br>. Serum<br>. Method: GC-ECD<br>. Lipid-adjusted	. PCB 138<br>. PCB 153<br>. PCB 180<br>. *p,p'*-DDE<br>. HCB	NR	p53-positive tumors<br>. PCB 138: OR=0.2 [0.1-1.0]	. Non-detectable values: imputed a random value between zero and detection limit<br>. Model selection: *a priori* selected covariates
Charlier Cj et al.<br>2007 [112]<br>Belgium	. Cross-sectional study<br>. n=125<br>. Prognostic factors: stage, histologic status, ER, grade, relapse	. Age: 60.7 ± 12.2 y<br>. Menopausal status: NR<br>. Invasive cancers<br>. % Invasive: 100%<br>. % ER+: 57%	. Timing: after chemotherapy and/or hormone therapy<br>. Serum<br>. Method: GC-MS<br>. Lipid-adjusted	Total pesticides (*p,p'*-DDT, *p,p'*-DDE, HCB, γ-HCH, aldrin)	Total pesticides higher in relapsing patients<br><br>*Adjustment for age only,*<br>Total pesticides higher in relapsing patients		. Non-detectable values: NR<br>. Model selection: NR
Demers A et al.<br>2000 [47]<br>Canada	. Cross-sectional study<br>. n=299<br>. Prognostic factors: tumor size ≥2cm, lymph node involvement	. Age: 53 ± 9 y<br>. Post-menopausal: 70%<br>. Invasive cancers<br>. % Invasive: 100%<br>. % ER+: 70%	. Timing: before chemotherapy or radiotherapy<br>. Plasma<br>. Method: GC-ECD<br>. Lipid-adjusted	. PCB 153<br>. *p,p'*-DDT<br>. *p,p'*-DDE<br>. Oxychlordane<br>. β-HCH<br>. *trans*-nonachlor<br>. *cis*-nonachlor<br>. α- and γ-chlordane<br>. HCB<br>. Mirex<br>. Aldrin	None	*Tert 3 vs Tert 1:*<br>*Tumor size ≥2cm*<br>. β-HCH: OR=2.3 [1.1-4.5]<br>. *trans*-nonachlor: OR=2.3 [1.1-4.7]<br><br>*Positive lymph-node*<br>. *p,p'*-DDE: OR=2.9 [1.4-5.9]<br>. PCB-153: OR=2.1 [1.1-4.3]<br>. Oxychlordane: OR=2.3 [1.1-5.0]<br><br>Positive lymph-node and tumor size ≥2cm<br>. *p,p'*-DDE: OR=3.5 [1.4-8.7]	. Non-detectable values: not analysed if >30 % of samples; imputed half the detection limit value<br>. Model selection: change of estimate (10%); ascending
**Breast adipose tissue**							
Ellsworth RE et al.<br>2018 [113]<br>USA	. Cross-sectional<br>. n=46<br>. Prognostic factors: stage, tumor grade, tumor size, node involvement, ER, HER2	. Age: 53.2 ± 10.1 y, range [34–79]<br>. Post-menopausal: NR<br>. Invasive and non-invasive cancers<br>. % Invasive: 85%<br>. % ER+: 62%	. Timing: at time of surgery<br>. Breast adipose<br>. Method: GC-ECD<br>. Lipid-adjusted	. 46 PCBs *(summed in 3 functional groups and total)*<br>. *p,p'*-DDE<br>. Mirex<br>. HCB<br>. Total organochlorine	*Invasive cancers only (adjusted for age and BMI):*<br>. *p,p'*-DDE: Higher in stage I vs stage II ; higher in luminal A vs luminal B and HER2+ tumors<br>. HCB: Higher in grade 1 vs grade 2 and 3 ; higher in luminal A vs HER2+ tumors<br>. Mirex: *Higher in lymph node negative vs positive patients*	NR	. Non-detectable values: assigned a zero value<br>. Model selection: NR
He Y et al.<br>2017 [73,74]<br>China	. Cross-sectional<br>. n=209<br>. Prognostic factors: stage, tumor size, node involvement, distant metastasis, ER, PR, HER2	. Age: 52.0 ± 9.9 y, range [25-80]<br>. Post-menopausal: 56%<br>. Invasive and non-invasive cancers<br>. % Invasive: 95%<br>. % ER+: 68%	. Timing: prior to chemotherapy and radiotherapy<br>. Breast adipose tissue for cases and breast and abdominal adipose tissue for controls<br>. Method: GC-MS<br>. Lipid-adjusted	. PCB 28<br>. PCB 52<br>. PCB 101<br>. PCB 118<br>. PCB 138<br>. PCB 153<br>. PCB 180<br><br>*(Individually and summed)*	. Total PCBs higher when higher stage and higher ER expression<br>. PCB 28, 138 and 153 higher when higher ER expression	NR	. Non-detectable values: imputed the detection limit value divided by the square root of 2<br>. Model selection: NR

Table S8. Studies of POPs and breast cancer prognostic factors *(continued)*

Study reference<br>Publication year<br>Location	Design	Patients and tumors characteristics[*]<br>(mean ± SD)	Exposure assessment	Evaluated POPs	Associated POPs<br>Estimate [95% confidence interval]<br>Without adjustment	 <br>With adjustment[**]	Comments
**Breast adipose tissue**							
Eldakroory SA et al.<br>2017 [114]<br>Egypt	. Cross-sectional<br>. n=70<br>. Prognostic factors: molecular markers of tumor aggressiveness	. Age: 54.8 ± 12.4 y, range [29-58]<br>. Post-menopausal: NR<br>. Invasiveness: NR<br>. % Invasive: NR<br>. % ER+: 64.3%	. Timing: prior to chemotherapy and radiotherapy<br>. Breast adipose and tumor tissue<br>. Method: GC-ECD<br>. Lipid-adjusted	. *p,p'*-DDT<br>. *p,p'*-DDD<br>. *p,p'*-DDE<br>. α-, β- and γ-HCH<br>. HCB<br>. Aldrin<br>. Endrin<br>. Heptachlor<br>. Heptachlor epoxide<br>. γ-chlordane<br>. Methoxychlor	*In tumor tissue ; quart4 vs quart1:*<br>. Methoxychlor:<br>. BcL2: r=-0.22<br>. PIM1: r=-0.25<br>. Annexin early apoptosis: r=-0.23<br>. G2m: r= 0.4<br>. Heptachlor, PIM1: r=-0.25<br>. HCB, Annexin necrotic: r=-0.25<br>. *p,p'*-DDE, G2m: r=0.3<br>. *p,p'*DDT, G2m: r=0.24	NR	. Non-detectable values: NR<br>. Model selection: NR<br>. Higher concentrations of methoxychlor, DDT, HCB, and chlordane in tumor tissue samples compared to the surrounding normal tissue
Arrebola JP et al.<br>2016 [111]<br>Spain	. Cross-sectional study<br>. n=103<br>. Prognostic factors: ER, PR, HER2, E-Cadherin, Ki-67, p53	. Age: 53.6 ± 11.8 y<br>. Post-menopausal: 41%<br>. Invasive cancers<br>. % Invasive: 100%<br>. % ER+: 86%	. Timing: at time of surgery<br>. Breast adipose tissue<br>. Method: GC-ECD<br>. Lipid-adjusted	. PCB 138<br>. PCB 153<br>. PCB 180<br>. *p,p'*-DDE<br>. HCB	NR	ER-positive tumors<br>. HCB: OR=28.4 [1.9-435.5]<br>PR-positive tumors<br>. *HCB:* OR =11.2 [1.7-73.3]<br>E-cad-positive tumors<br>HCB: OR=0.01 [0.0-0.3]	. Non-detectable values: imputed a random value between zero and detection limit<br>. Model selection: *a priori* selected covariates
Barr L et al.<br>2012 [115]<br>UK	. Cross-sectional study<br>. n=40<br>. Prognostic factors: ER status	. Age: mean=65 y, range [37-91]<br>. Post-menopausal: NR<br>. Invasiveness: NR<br>. % Invasive: NR<br>. % ER+: 27/40	. Timing: at time of surgery<br>. Breast adipose tissue<br>. Method: HLPC-MS-MS<br>. Lipid-adjustment: NR	. Methylparaben<br>. Ethylparaben<br>. n-propylparaben<br>. n-butylparaben<br>. Benzylparaben	None	NR	. Non-detectable values: NR<br>. Model selection: NR
Muñoz-de-Toro M et al. 2006 [116]<br>Argentina	. Cross-sectional study<br>. n=55<br>. Prognostic factors: node involvement, tumor size, Ki-67, ER, PR	. Age: 60.5 ± 11. 6 y<br>. Post-menopausal: 76%<br>. Invasive cancers<br>. % Invasive: 100%<br>. % ER+: 50%	. Timing: At the time of biopsy<br>. Breast adipose tissue<br>. Method: GC-ECD<br>. Lipid-adjusted (ppb)	. 8 PCBs<br>. 18 other organochlorines<br><br>*(individually and summed)*	Total organochlorines<br>. PR expression : r_s= 0.359<br>. Ki-67 : positive correlation r_s= NR *(post-menopausal)*<br><br>*p,p'*-DDE<br>. PR expression positive correlation r_s= NR<br>*(among ER-positive tumors)*	NR	. Non-detectable values: imputed the mean between zero and the detection limit<br>. Model selection: NR
**Adipose tissue other than breast**							
Roswall N et al.<br>2018 [109]<br>Raaschou-Nielsen O et al. 2005 [104]<br>Denmark	. Cross-sectional analysis<br>. n=409<br>. Prognostic factors: tumor size, lymph node involvement	. Age: median=60 y<br>. Post-menopausal: 100%<br>. Invasiveness: NR<br>. % Invasive: NR<br>. % ER+: 77.5%	. Timing: not considered<br>. Buttock adipose tissue<br>. Method: GC-MS<br>. Lipid-adjusted (µg/kg lipids)	. Total of 18 PCBs<br>. *p,p'*-DDE<br><br>*(individually and summed PCBs)*	None	*NR*	. Non-detectable values: >50% of samples for 8 individual PCBs (not analysed); contributed zero to the PCB sum ; excluded for other organochlorines<br>. Model selection: NR

POPs: persistent organic pollutants; [*]at diagnosis; SD: Standard deviation; y: years; NR: Not reported; NA: Not applicable; MS: Mass spectrometry; LD: limit-detection; LC-MS-MS: liquid chromatography–tandem mass spectrometry; GC-MS-MS: gas chromatography-tandem mass spectrometry; GC-IDMS: gas-chromatography isotope-dilution mass-spectrometry; GC-ECD: gas chromatography with electron Capture Detector; GC-ID-HRMS: gas chromatography-isotope dilution high-resolution mass spectrometry ; HR-GC-ECD: high-resolution gas chromatography with microelectron capture detection ; HLPC: High-performance liquid chromatography; PFCs: perfluorinated compounds
Prospective/retrospective/cross-sectional: timing of sample in regards to breast cancer diagnosis ; Lag time: time between blood draw and breast cancer diagnosis ; in bold : compounds actually analysed;
**only when all important confounders were considered for adjustment.

Table S9. Results of studies of POPs and breast cancer risk

POPs	Total number of studies	N studies with measures in blood (plasma/serum)			N studies with measures in breast adipose tissue			N studies with measures in adipose tissue other than breast			N studies with measures in breast tumor		
		Total studies	N studies with positive association*	N studies with negative association**	Total studies	N studies with positive association**	N studies with negative association**	Total studies	N studies with positive association**	N studies with negative association**	Total studies	N studies with positive association**	N studies with negative association**
Total	**85**	**61**			**26**			**2**			**4**		
PCBs	**57**	**38**			**16**			**1**			**3**		
Organochlorines	**69**	**48**			**21**			**2**			**4**		
o,p'-DDT	17	14	1	1	4	0	0	2	0	0	2	0	0
p,p'-DDT	40	30	4	1	12	0	0	3	0	0	2	0	0
o,p'-DDE	10	9	0	0	2	0	0	0	NA	NA	1	0	0
p,p'-DDE	44	33	5	0	13	2	0	3	0	1	2	0	0
p,p'-DDD	2	2	1	0	1	0	0	0	NA	NA	0	NA	NA
DDT*	12	8	1	0	4	0	0	2	0	0	0	NA	NA
DDE*	19	13	2	0	6	2	0	1	0	1	0	NA	NA
HCB	34	22	2	0	14	1	0	1	0	1	0	NA	NA
α-Chlordane	9	4	0	0	4	0	0	1	0	0	0	NA	NA
γ-Chlordane	8	4	0	0	3	0	0	1	0	0	0	NA	NA
Oxychlordane	20	12	0	0	9	0	0	1	0	1	0	NA	NA
cis-Nonachlor	7	3	0	1	3	0	0	1	0	0	0	NA	NA
trans-Nonachlor	20	12	0	1	9	0	0	1	0	1	0	NA	NA
α-HCH	9	6	0	0	5	0	0	0	NA	NA	1	0	0
β-HCH	30	22	3	0	13	1	0	1	0	1	0	NA	NA
γ-HCH (Lindane)	16	10	0	0	8	1	0	0	NA	NA	1	0	0
δ-HCH	5	4	1	0	2	0	0	0	NA	NA	1	0	0
Aldrin	11	8	0	0	4	1	0	1	0	0	0	NA	NA
Dieldrin	17	13	2	0	5	0	0	1	0	0	0	NA	NA
Endrin	12	7	0	0	5	0	0	1	0	0	0	NA	NA
Mirex	14	8	0	1	6	0	0	1	0	0	0	NA	NA
Heptachlor	10	8	1	0	3	0	0	0	NA	NA	0	NA	NA
Heptachlor Epoxide	13	7	0	0	6	1	0	1	0	0	0	NA	NA
Toxaphene	1	0	NA	NA	1	0	0	0	NA	NA	0	NA	NA
Total PCB	28	21	8	1	6	0	0	1	0	1	2	0	0
PCB 8	1	1	1	0	0	NA	NA	0	NA	NA	0	NA	NA
PCB 28	17	11	0	0	5	1	0	2	0	0	0	NA	NA
PCB 49	1	0	NA	NA	0	NA	NA	0	NA	NA	1	0	0
PCB 52	16	8	1	0	5	0	0	2	0	0	1	0	0
PCB 66	8	8	0	0	0	NA	NA	0	NA	NA	0	NA	NA
PCB 70	1	0	NA	NA	0	NA	NA	0	NA	NA	1	0	0
PCB 74	15	13	0	0	4	0	0	0	NA	NA	0	NA	NA
PCB 77	2	1	0	0	1	1	0	0	NA	NA	0	NA	NA
PCB 99	21	16	0	0	5	0	0	2	0	0	0	NA	NA
PCB 101	21	13	0	0	5	0	0	2	0	0	1	0	0
PCB 105	21	9	0	0	4	2	0	3	0	0	1	0	0
PCB 110	1	0	NA	NA	0	NA	NA	0	NA	NA	1	0	0
PCB 118	32	23	3	0	10	1	0	2	0	1	1	0	0
PCB 128	8	4	0	0	3	0	0	2	0	0	0	NA	NA

Table S9. Results of studies of POPs and breast cancer risk *(continued)*

POPs	Total number of studies	N studies with measures in blood (plasma/serum)			N studies with measures in breast adipose tissue			N studies with measures in adipose tissue other than breast			N studies with measures in breast tumor		
		Total studies	N studies with positive association**	N studies with negative association**	Total studies	N studies with positive association**	N studies with negative association**	Total studies	N studies with positive association**	N studies with negative association**	Total studies	N studies with positive association**	N studies with negative association**
PCB 137	1	0	NA	NA	0	NA	NA	0	NA	NA	1	0	0
PCB 138	35	25	4	0	10	0	0	2	0	1	1	0	0
PCB 146	8	8	0	1	0	0	0	NA	NA	NA	0	NA	NA
PCB 153	35	26	3	1	10	0	1	2	0	1	1	0	0
PCB 156	28	20	1	0	9	0	1	2	0	0	1	0	0
PCB 163	1	1	0	0	0	NA	NA	NA	NA	NA	NA	NA	NA
PCB 167	9	9	0	1	0	NA	NA	0	NA	NA	0	NA	NA
PCB 169	1	0	NA	NA	0	NA	NA	0	NA	NA	1	0	0
PCB 170	31	21	2	0	10	0	0	2	0	0	1	0	0
PCB 172	7	7	0	0	1	0	0	0	NA	NA	0	NA	NA
PCB 177	6	5	0	0	0	NA	NA	0	NA	NA	1	0	0
PCB 178	9	9	0	0	1	0	0	0	NA	NA	0	NA	NA
PCB 180	39	28	3	0	10	1	0	2	0	0	1	0	0
PCB 183	30	21	1	1	9	1	0	2	0	0	2	0	0
PCB 187	30	21	1	1	9	0	0	2	0	0	1	0	0
PCB 189	1	0	NA	NA	0	NA	NA	0	NA	NA	1	0	0
PCB 194	7	7	0	0	0	NA	NA	0	NA	NA	0	NA	NA
PCB 195	8	8	1	0	0	NA	NA	0	NA	NA	0	NA	NA
PCB 196	2	2	0	1	0	NA	NA	0	NA	NA	0	NA	NA
PCB 199	2	2	0	0	0	NA	NA	0	NA	NA	0	NA	NA
PCB 201	1	1	0	0	0	NA	NA	0	NA	NA	0	NA	NA
PCB 203	9	9	1	0	0	NA	NA	0	NA	NA	0	NA	NA
PCB 206	6	6	1	0	0	NA	NA	0	NA	NA	0	NA	NA
PCB 209	3	3	1	0	0	NA	NA	0	NA	NA	0	NA	NA
PCB 203/(167+187)	1	1	1	0	0	NA	NA	0	NA	NA	0	NA	NA
PCB 28+52+101	1	1	0	1	0	NA	NA	0	NA	NA	0	NA	NA
Dioxins	4	2	0	0	2	0	0	0	NA	NA	0	NA	NA
PFAS*	1	1	0	0	0	NA	NA	0	NA	NA	0	NA	NA
PFAAs*	1	1	1	0	0	NA	NA	0	NA	NA	0	NA	NA
PFSAs*	1	1	1	0	0	NA	NA	0	NA	NA	0	NA	NA
PFOSA	2	2	1	0	0	NA	NA	0	NA	NA	0	NA	NA
PFHxS	3	3	1	1	0	NA	NA	0	NA	NA	0	NA	NA
PFOA	1	1	1	0	0	NA	NA	0	NA	NA	0	NA	NA
PFNA	1	1	0	0	0	NA	NA	0	NA	NA	0	NA	NA
PFUnDA	1	1	0	0	0	NA	NA	0	NA	NA	0	NA	NA
PFOS	2	2	1	0	0	NA	NA	0	NA	NA	0	NA	NA
MeFOSAA	1	1	0	0	0	NA	NA	0	NA	NA	0	NA	NA
Mono-ethyl phthalate	1	1	1	0	0	NA	NA	0	NA	NA	0	NA	NA
Parabens	1	1	0	0	0	NA	NA	0	NA	NA	0	NA	NA
BPA	2	2	1	0	0	NA	NA	0	NA	NA	0	NA	NA
PBBs	1	1	1	0	0	NA	NA	0	NA	NA	0	NA	NA
PBDE*	1	0	NA	NA	1	1	0	0	NA	NA	0	NA	NA
BDE 28	1	0	NA	NA	1	1	0	0	NA	NA	0	NA	NA

Table S9. Results of studies of POPs and breast cancer risk *(continued)*

POPs	Total number of studies	N studies with measures in blood (plasma/serum)			N studies with measures in breast adipose tissue			N studies with measures in adipose tissue other than breast			N studies with measures in breast tumor		
		Total studies	N studies with positive association**	N studies with negative association**	Total studies	N studies with positive association**	N studies with negative association**	Total studies	N studies with positive association**	N studies with negative association**	Total studies	N studies with positive association**	N studies with negative association**
BDE 47	1	0	NA	NA	1	1	0	0	NA	NA	0	NA	NA
BDE 71	1	0	NA	NA	1	0	1	0	NA	NA	0	NA	NA
BDE 99	1	0	NA	NA	1	1	0	0	NA	NA	0	NA	NA
BDE 100	1	0	NA	NA	1	1	0	0	NA	NA	0	NA	NA
BDE 138	1	0	NA	NA	1	1	0	0	NA	NA	0	NA	NA
BDE 153	1	0	NA	NA	1	1	0	0	NA	NA	0	NA	NA
BDE 154	1	0	NA	NA	1	1	0	0	NA	NA	0	NA	NA
BDE 183	1	0	NA	NA	1	0	0	0	NA	NA	0	NA	NA
BDE 190	1	0	NA	NA	1	0	0	0	NA	NA	0	NA	NA
BDE 209	1	0	NA	NA	1	1	0	0	NA	NA	0	NA	NA

POPs: persistent organic pollutants; * Total or not specified; NA: Not applicable; ** only when all important confounders were considered for adjustment; positive association: higher risk with higher levels of the POP; negative association : lower risk with higher levels of the POP.

Table S10. Results of studies of POPs and mortality in breast cancer patients

POPs	Total studies	N studies with measures in blood (plasma/serum)			N studies with measures in breast adipose tissue			N studies with measures in adipose tissue other than breast		
		Total studies	N studies with positive association**	N studies with negative association**	Total studies	N studies with positive association**	N studies with negative association**	Total studies	N studies with positive association**	N studies with negative association**
Total	**6**	**3**	-	-	**1**	-	-	**2**	-	-
PCBs	**4**	**3**	-	-	**1**	-	-	**1**	-	-
Organochlorines	**5**	**3**	-	-	**1**	-	-	**2**	-	-
p,p'-DDT	3	3	1	0	0	NA	NA	1	0	1
p,p'-DDE	3	3	0	1	1	0	0	1	0	1
DDT*	3	2	0	0	1	0	0	0	NA	NA
DDE*	1	0	NA	NA	0	NA	NA	1	0	1
DDD*	1	0	NA	NA	1	0	0	0	NA	NA
HCB	3	1	0	0	1	0	0	1	0	1
Chlordane*	1	1	0	0	0	NA	NA	0	NA	NA
α-chlordane	1	0	NA	NA	0	NA	NA	1		
γ-chlordane	1	0	NA	NA	0	NA	NA	1	0	0
Oxychlordane	2	0	NA	NA	1	0	0	1	0	0
trans-nonachlor	2	0	NA	NA	1	0	0	1	0	1
cis-Nonachlor	1	0	NA	NA	0	NA	NA	1	0	1
Heptachlor epoxide	1	0	NA	NA	0	NA	NA	1		
β-HCH	2	1	0	NA	1	0	0	1	NA	NA
Aldrin	1	0	NA	NA	0	NA	NA	1	0	0
Dieldrin	3	2	2	0	0	NA	NA	1	1	0
Endrin	1	0	NA	NA	0	NA	NA	1	0	0
Mirex	1	0	NA	NA	0	NA	NA	1	0	0
Total PCB	4	3	1	0	1	1	0	1	0	1
PCB 28	2	1	0	0	0	NA	NA	1	0	0
PCB 52	2	1	0	0	0	NA	NA	1	0	0
PCB 54	1	0	NA	NA	0	NA	NA	1	0	0
PCB 66	1	1	0	0	0	NA	NA	0	NA	NA
PCB 74	2	1	0	0	1	0	0	0	NA	NA
PCB 99	3	1	0	0	1	0	0	1	0	1
PCB 101	2	1	0	0	0	NA	NA	1	0	0
PCB 104	1	0	NA	NA	0	NA	NA	1	0	0
PCB 105	2	1	0	0	0	NA	NA	1	0	0
PCB 118	3	1	0	0	1	1	0	1	0	1
PCB 128	1	0	NA	NA	0	NA	NA	1	0	0
PCB 138	3	1	0	0	1	0	0	1	0	1
PCB 146	2	1	0	0	1	0	0	0	NA	NA
PCB 153	3	1	0	0	1	1	0		0	1
PCB 155	1	0	NA	NA	0	NA	NA	1	0	0
PCB 156	3	1	0	0	1	0	0	1	0	1
PCB 167	2	1	0	0	1	1	0	0	NA	NA
PCB 170	3	1	0	0	1	0	0	1	0	1
PCB 172	2	1	0	0	1	0	0	0	NA	NA
PCB 174	1	1	1	0	0	NA	NA	0	NA	NA

Table S10. Results of studies of POPs and mortality in breast cancer patients

POPs	Total studies	N studies with measures in blood (plasma/serum)			N studies with measures in breast adipose tissue			N studies with measures in adipose tissue other than breast		
		Total studies	N studies with positive association**	N studies with negative association**	Total studies			Total studies	N studies with positive association**	N studies with negative association**
PCB 177	2	2	1	0	0	NA	NA	0	NA	NA
PCB 178	2	1	0	0	1	0	0	0	NA	NA
PCB 180	3	1	0	0	1	0	0	1	0	1
PCB 183	3	1	0	0	1	0	0	1	0	1
PCB 187	3	1	0	0	1	0	0	1	0	1
PCB 193	1	1	0	0	0	NA	NA	0	NA	NA
PCB 199	1	1	0	0	0	NA	NA	0	NA	NA
PCB 200	1	1	0	0	0	NA	NA	0	NA	NA
PCB 201	1	0	NA	NA	0	NA	NA	1	0	1
PCB 203	1	1	0	0	0	NA	NA	0	NA	NA

POPs: persistent organic pollutants; * Total or not specified ; ** only when all important confounders were considered for adjustment; positive association : higher mortality with higher levels of the POP; negative association : lower mortality with higher levels of the POP; NB: no study evaluated the association of POPs as measured in breast tumors and mortality among breast cancer patients.

Table S11. Results of studies of POPs and prognostic factors in breast cancer patients

POPs	Total studies	N studies with measures in blood (plasma/serum)			N studies with measures in breast adipose tissue			N studies with measures in adipose tissue other than breast			N studies with measures in breast tumor		
		Total studies	N studies with positive association**	N studies with negative association**	Total studies	N studies with positive association**	N studies with negative association**	Total studies	N studies with positive association**	N studies with negative association**	Total studies	N studies with positive association**	N studies with negative association**
Total	**9**	**3**			**6**			**1**			**1**		
PCBs	**4**	**2**			**4**			**1**			**0**		
Organochlorines	**6**	**3**			**4**			**1**			**1**		
o,p'-DDT	1	0	NA	NA	1	0	0	0	NA	NA	0	NA	NA
p,p'-DDT	4	2	0	0	2	0	0	0	NA	NA	1	0	0
o,p'-DDE	1	0	NA	NA	1	0	0	0	NA	NA	0	NA	NA
p,p'-DDE	5	3	1	0	3	0	0	1	0	0	1	0	0
p,p'-DDD	1	0	NA	NA	1	0	0	0	NA	NA	1	0	0
DDT*	1	0	NA	NA	1	0	0	0	NA	NA	0	NA	NA
HCB	4	2	0	0	4	0	0	0	NA	NA	1	0	0
α-Chlordane	1	0	NA	NA	1	0	0	0	NA	NA	0	NA	NA
γ-Chlordane	2	0	NA	NA	2	0	0	0	NA	NA	1	0	0
Oxychlordane	2	1	1	0	1	0	0	0	NA	NA	0	NA	NA
Trans-Nonachlor	1	1	1	0	0	NA	NA	0	NA	NA	0	NA	NA
Methoxychlor	1	0	NA	NA	1	0	0	0	NA	NA	1	0	0
α-HCH	2	0	NA	NA	2	0	0	0	NA	NA	1	0	0
β-HCH	3	1	1	0	2	0	0	0	NA	NA	1	0	0
γ-HCH (Lindane)	3	1	0	0	2	0	0	0	NA	NA	1	0	0
Aldrin	3	1	0	0	2	0	0	0	NA	NA	1	0	0
Dieldrin	1	0	NA	NA	1	0	0	0	NA	NA	0	NA	NA
Endrin	2	0	NA	NA	2	0	0	0	NA	NA	1	0	0
Mirex	1	0	NA	NA	2	0	0	0	NA	NA	0	NA	NA
Heptachlor	2	0	NA	NA	2	0	0	0	NA	NA	1	0	0
Heptachlor Epoxide	2	0	NA	NA	2	0	0	0	NA	NA	1	0	0
Total pesticides*	1	1	1	0	0	NA	NA	0	NA	NA	0	NA	NA
Total PCB	1	0	NA	NA	1	0	0	1	0	0	0	NA	NA
PCB 28	1	0	NA	NA	1	0	0	0	NA	NA	0	NA	NA
PCB 52	1	0	NA	NA	1	0	0	0	NA	NA	0	NA	NA
PCB 77	1	0	NA	NA	1	0	0	0	NA	NA	0	NA	NA
PCB 101	1	0	NA	NA	1	0	0	0	NA	NA	0	NA	NA
PCB 118	2	0	NA	NA	2	0	0	0	NA	NA	0	NA	NA
PCB 128	1	0	NA	NA	1	0	0	0	NA	NA	0	NA	NA
PCB 138	3	1	1	0	3	0	0	0	NA	NA	0	NA	NA
PCB 153	3	2	1	0	2	0	0	0	NA	NA	0	NA	NA
PCB 170	1	0	NA	NA	1	0	0	0	NA	NA	0	NA	NA
PCB 180	3	1	0	0	3	0	0	0	NA	NA	0	NA	NA
PCB 187	1	0	NA	NA	1	0	0	0	NA	NA	0	NA	NA
PCB 195	1	0	NA	NA	1	0	0	0	NA	NA	0	NA	NA
Parabens	1	0	NA	NA	1	0	0	0	NA	NA	0	NA	NA

POPs: persistent organic pollutants; * Total or not specified; ** only when all important confounders were considered for adjustment; positive association : higher level of a prognostic factor with higher levels of the POP; negative association : lower level of a prognostic facto with higher levels of the POP.

Table S12. Summary results of POPs associated[**] positively with breast cancer risk

Criteria	POPs	
	n	Compounds
**Measures in blood (plasma/serum), n=61 studies**		
Positive association in at least one study	35	*o,p'*-DDT, *p,p'*-DDT, *p,p'*-DDE, *p,p'*-DDD, DDT*, DDE*, HCB, β-HCH, δ-HCH, Dieldrin, Heptachlor, Total PCBs, PCB 8, PCB 52, PCB 118, PCB 138, PCB 153, PCB 156, PCB 170, PCB 180, PCB 183, PCB 187, PCB 195, PCB 203, PCB 206, PCB 209, PFAAs, PFSAs, PFOSA, PFOS, PFOA, PFHxS, Monoethyl phthalate, BPA, PBBs
Positive association in at least one study and no negative association	28	***p,p'*-DDE**, *p,p'*-DDD, DDT*, **DDE***, **HCB**, **β-HCH**, δ-HCH, Dieldrin, Heptachlor, PCB 8, PCB 52, **PCB 118**, PCB 138, PCB 156, PCB 170, **PCB 180**, PCB 195, PCB 203, PCB 206, PCB 209, PFAAs, PFSAs, PFOSA, PFOA, PFOS, Mono-ethyl phthalate, BPA, PBBs
Positive association in ≥10 % of studies and in at least 2 studies	11	*p,p'*-DDT, *p,p'*-DDE, DDE*, β-HCH, Dieldrin, Total PCBs, PCB 118, PCB 138, PCB 153, PCB 170, PCB 180
Positive association in ≥10 % of studies and in at least 2 studies and no negative association	8	*p,p'*-DDE, DDE*, β-HCH, Dieldrin, PCB 118, PCB 138, PCB 170, PCB 180
**Measures in breast adipose tissue, n=26 studies**		
Positive association in at least one study	21	*p,p'*-DDE, DDE*, HCB, β-HCH, γ-HCH, Aldrin, Heptachlor epoxide, PCB 28, PCB 105, PCB 118, PCB 180, PCB 183, PBDE*, BDE 28, BDE 47, BDE 99, BDE 100, BDE 138, BDE 153, BDE 154, BDE 209
Positive association in at least one study and no negative association	21	***p,p'*-DDE**, **DDE***, **HCB**, **β-HCH**, γ-HCH, Aldrin, Heptachlor epoxide, PCB 28, PCB 105, **PCB 118**, **PCB 180**, PCB 183, PBDE*, BDE28, BDE 47, BDE 99, BDE 100, BDE 138, BDE 153, BDE 154, BDE 209
Positive association in ≥10 % of studies and in at least 2 studies	3	*p,p'*-DDE, DDE*, PCB 105
Positive association in ≥10 % of studies and in at least 2 studies and no negative association	3	*p,p'*-DDE, DDE*, PCB 105
**Measures in adipose tissue other than breast, n=2 studies**		
Positive association in at least one study	0	None
Positive association in at least one study and no negative association	0	None
Positive association in ≥10 % of studies and in at least 2 studies	0	None
Positive association in ≥10 % of studies and in at least 2 studies and no negative association	0	None
**Studies with measures in breast tumor, n=2**		
Positive association in at least one study	0	None
Positive association in at least one study and no negative association	0	None
Positive association in ≥10 % of studies and in at least 2 studies	0	None
Positive association in ≥10 % of studies and in at least 2 studies and no negative association	0	None

POPs: persistent organic pollutants; *Total or not specified; **in Bold:** compounds in common between different sample types; [**] only when all important confounders were considered for adjustment; positive association : higher level of a prognostic factor with higher levels of the POP; negative association : lower level of a prognostic facto with higher levels of the POP.

Table S13. Summary results of POPs associated** negatively with breast cancer risk

Criteria	POPs	
	n	Compounds
**Measures in blood (plasma/serum), n=61studies**		
Negative association in at least one study	13	*o,p'*-DDT, *p,p'*-DDT, *cis*-nonachlor, *trans*-nonachlor, Mirex, Total PCB, PCB 146, PCB 153, PCB 167, PCB 183, PCB 187, PCB 196, PFHxS
Negative association in at least one study and no positive association	6	*cis*-nonachlor, ***trans*-nonachlor**, Mirex, PCB 146, PCB 167, PCB 196
Negative association in ≥10 % of studies and in at least 2 studies	0	None
Negative association in ≥10 % of studies and in at least 2 studies and no positive association	0	None
**Measures in breast adipose tissue, n=26 studies**		
Negative association in at least one study	3	PCB 153, PCB 156, BDE 71
Negative association in at least one study and no positive association	3	PCB 153, PCB 156, BDE 71
Negative association in ≥10 % of studies and in at least 2 studies	0	None
Negative association in ≥10 % of studies and in at least 2 studies and no positive association	0	None
**Measures in adipose tissue other than breast, n=2 studies**		
Negative association in at least one study	10	*p,p'*-DDE, DDE*, HCB, Oxychlordane, *trans*-nonachlor, β-HCH, Total PCBs, PCB 118, PCB 138, PCB 153
Negative association in at least one study and no positive association	10	*p,p'*-DDE, DDE*, HCB, Oxychlordane, ***trans*-nonachlor**, β-HCH, Total PCB, PCB 118, PCB 138, PCB 153
Negative association in ≥10 % of studies and in at least 2 studies	0	None
Negative association in ≥10 % of studies and in at least 2 studies and no positive association	0	None
**Measures in breast tumor, n=2 studies**		
Negative association in at least one study	2	α-HCH, γ-HCH
Negative association in at least one study and no positive association	2	α-HCH, γ-HCH
Negative association in ≥10 % of studies and in at least 2 studies	0	None
Negative association in ≥10 % of studies and in at least 2 studies and no positive association	0	None

POPs: persistent organic pollutants; *Total or not specified; **in Bold**: compounds in common between different samples; ** only when all important confounders were considered for adjustment; positive association : higher level of a prognostic factor with higher levels of the POP; negative association : lower level of a prognostic facto with higher levels of the POP.

Table S14. Summary results of POPs associated positively with mortality among breast cancer patients**

Criteria	POPs	
	n	Compounds
**Measures in blood (plasma/serum), n=3 studies**		
Positive association in at least one study	5	*p,p'*-DDT, Dieldrin, Total PCBs, PCB 174, PCB 177
Positive association in at least one study and no negative association	5	*p,p'*-DDT, Dieldrin, **Total PCBs**, PCB 174, PCB 177
Positive association in ≥10 % of studies and in at least 2 studies	1	Dieldrin
Positive association in ≥10 % of studies and in at least 2 studies and no negative association	0	Dieldrin
**Measures in breast adipose tissue, n=1 study**		
Positive association in at least one study	4	Total PCBs, PCB 118, PCB 153, PCB 167
Positive association in at least one study and no negative association	4	**Total PCBs**, PCB 118, PCB 153, PCB 167
Positive association in ≥10 % of studies and in at least 2 studies	0	None
Positive association in ≥10 % of studies and in at least 2 studies and no negative association	0	None
**Measures in adipose tissue other than breast, n=2 studies**		
Positive association in at least one study	1	Dieldrin
Positive association in at least one study and no negative association	1	Dieldrin
Positive association in ≥10 % of studies and in at least 2 studies	0	None
Positive association in ≥10 % of studies and in at least 2 studies and no negative association	0	None

POPs: persistent organic pollutants; * Total or not specified; **in Bold:** compounds in common between different samples; ** only when all important confounders were considered for adjustment; positive association: higher level of a prognostic factor with higher levels of the POP; negative association : lower level of a prognostic facto with higher levels of the POP; NB: no study evaluated the association of POPs as measured in breast tumors and mortality among breast cancer patients.

Table S15. Summary results of POPs associated negatively with mortality among breast cancer patients**

Criteria	POPs	
	n	Compounds
**Measures in blood (plasma/serum), n=3 studies**		
Negative association in at least one study	1	*p,p'*-DDE
Negative association in at least one study and no positive association	1	*p,p'*-DDE
Negative association in ≥10 % of studies and in at least 2 studies	0	None
Negative association in ≥10 % of studies and in at least 2 studies and no positive association	0	None
**Measures in breast adipose tissue, n=1 study**		
Negative association in at least one study	0	None
Negative association in at least one study and no positive association	0	None
Negative association in ≥10 % of studies and in at least 2 studies	0	None
Negative association in ≥10 % of studies and in at least 2 studies and no positive association	0	None
**Measures in adipose tissue other than breast, n=2 studies**		
Negative association in at least one study	17	*p,p'*-DDT, *p,p'*-DDE, DDE*, HCB, *trans*-nonachlor, *cis*-nonachlor, Total PCBs, PCB 99, PCB 118, PCB 153, PCB 156, PCB 138, PCB 170, PCB 180, PCB 183, PCB 187, PCB 201
Negative association in at least one study and no positive association	17	*p,p'*-DDT, *p,p'*-DDE, DDE*, HCB, *trans*-nonachlor, *cis*-nonachlor, Total PCBs, PCB 99, PCB 118, PCB 153, PCB 156, PCB 138, PCB 170, PCB 180, PCB 183, PCB 187, PCB 201
Negative association in ≥10 % of studies and in at least 2 studies	0	None
Negative association in ≥10 % of studies and in at least 2 studies and no positive association	0	None

POPs: persistent organic pollutants; * Total or not specified; **in Bold**: compounds in common between different samples; ** only when all important confounders were considered for adjustment; positive association: higher level of a prognostic factor with higher levels of the POP; negative association : lower level of a prognostic facto with higher levels of the POP ; NB: no study evaluated the association of POPs as measured in breast tumors and mortality among breast cancer patients.

Table S16. Summary results of POPs associated positively with breast cancer prognostic factors**

Criteria	POPs	
	n	Compounds
**Measures in blood (plasma/serum), n=3 studies**		
Positive association in at least one study	7	*p,p'*-DDE, Oxychlordane, *trans*-Nonachlor, β-HCH, total pesticides*, PCB 138, PCB 153
Positive association in at least one study and no negative association	7	*p,p'*-DDE, Oxychlordane, *trans*-Nonachlor, total pesticides*, β-HCH, PCB 138, PCB 153
Positive association in ≥10 % of studies and in at least 2 studies	0	None
Positive association in ≥10 % of studies and in at least 2 studies and no negative association	0	None
**Measures in breast adipose tissue, n=6 studies**		
Positive association in at least one study	0	None
Positive association in at least one study and no negative association	0	None
Positive association in ≥10 % of studies and in at least 2 studies	0	None
Positive association in ≥10 % of studies and in at least 2 studies and no negative association	0	None
**Measures in adipose tissue other than breast, n=1 study**		
Positive association in at least one study	0	None
Positive association in at least one study and no negative association	0	None
Positive association in ≥10 % of studies and in at least 2 studies	0	None
Positive association in ≥10 % of studies and in at least 2 studies and no negative association	0	None
**Measures in breast tumor, n=1 study**		
Positive association in at least one study	0	None
Positive association in at least one study and no negative association	0	None
Positive association in ≥10 % of studies and in at least 2 studies	0	None
Positive association in ≥10 % of studies and in at least 2 studies and no negative association	0	None

POPs: persistent organic pollutants; * Total or not specified; **in Bold:** compounds in common between different samples
NB: no study reported a negative association between POPs and breast cancer prognostic factors regardless of the sample type

References:

1. Warner M, Mocarelli P, Samuels S, Needham L, Brambilla P, Eskenazi B. Dioxin exposure and cancer risk in the Seveso Women's Health Study. Environmental health perspectives. 2011;119(12):1700-1705.
2. Pesatori AC, Consonni D, Rubagotti M, Grillo P, Bertazzi PA. Cancer incidence in the population exposed to dioxin after the "Seveso accident": twenty years of follow-up. Environmental health : a global access science source. 2009;8:39.
3. Warner M, Eskenazi B, Mocarelli P, Gerthoux PM, Samuels S, Needham L, Patterson D, Brambilla P. Serum dioxin concentrations and breast cancer risk in the Seveso Women's Health Study. Environmental health perspectives. 2002;110(7):625-628.
4. Bonefeld-Jorgensen EC, Long M, Fredslund SO, Bossi R, Olsen J. Breast cancer risk after exposure to perfluorinated compounds in Danish women: a case-control study nested in the Danish National Birth Cohort. Cancer causes & control : CCC. 2014;25(11):1439-1448.
5. Hurley S, Goldberg D, Wang M, Park JS, Petreas M, Bernstein L, Anton-Culver H, Nelson DO, Reynolds P. Breast cancer risk and serum levels of per- and poly-fluoroalkyl substances: a case-control study nested in the California Teachers Study. Environmental health : a global access science source. 2018;17(1):83.
6. Cohn BA, La Merrill M, Krigbaum NY, Yeh G, Park JS, Zimmermann L, Cirillo PM. DDT Exposure in Utero and Breast Cancer. The Journal of clinical endocrinology and metabolism. 2015;100(8):2865-2872.
7. Cohn BA, Terry MB, Plumb M, Cirillo PM. Exposure to polychlorinated biphenyl (PCB) congeners measured shortly after giving birth and subsequent risk of maternal breast cancer before age 50. Breast cancer research and treatment. 2012;136(1):267-275.
8. Cohn BA, Wolff MS, Cirillo PM, Sholtz RI. DDT and breast cancer in young women: new data on the significance of age at exposure. Environmental health perspectives. 2007;115(10):1406-1414.
9. Iwasaki M, Inoue M, Sasazuki S, Kurahashi N, Itoh H, Usuda M, Tsugane S. Plasma organochlorine levels and subsequent risk of breast cancer among Japanese women: a nested case-control study. The Science of the total environment. 2008;402(2-3):176-183.
10. Rubin CH, Lanier A, Kieszak S, Brock JW, Koller KR, Strosnider H, Needham L, Zahm S, Harpster A. Breast cancer among Alaska Native women potentially exposed to environmental organochlorine chemicals. International journal of circumpolar health. 2006;65(1):18-27.
11. Wolff MS, Zeleniuch-Jacquotte A, Dubin N, Toniolo P. Risk of breast cancer and organochlorine exposure. Cancer epidemiology, biomarkers & prevention : a publication of the American Association for Cancer Research, cosponsored by the American Society of Preventive Oncology. 2000;9(3):271-277.
12. Terrell ML, Rosenblatt KA, Wirth J, Cameron LL, Marcus M. Breast cancer among women in Michigan following exposure to brominated flame retardants. Occupational and environmental medicine. 2016;73(8):564-567.
13. Ward EM, Schulte P, Grajewski B, Andersen A, Patterson DG, Jr., Turner W, Jellum E, Deddens JA, Friedland J, Roeleveld N; et al. Serum organochlorine levels and breast cancer: a nested case-control study of Norwegian women. Cancer epidemiology, biomarkers & prevention : a publication of the American Association for Cancer Research, cosponsored by the American Society of Preventive Oncology. 2000;9(12):1357-1367.
14. Helzlsouer KJ, Alberg AJ, Huang HY, Hoffman SC, Strickland PT, Brock JW, Burse VW, Needham LL, Bell DA, Lavigne JA; et al. Serum concentrations of organochlorine compounds and the subsequent development of breast cancer. Cancer epidemiology, biomarkers & prevention : a publication of the American Association for Cancer Research, cosponsored by the American Society of Preventive Oncology. 1999;8(6):525-532.
15. Dorgan JF, Brock JW, Rothman N, Needham LL, Miller R, Stephenson HE, Jr., Schussler N, Taylor PR. Serum organochlorine pesticides and PCBs and breast cancer risk: results from a prospective analysis (USA). Cancer causes & control : CCC. 1999;10(1):1-11.
16. Hoyer AP, Jorgensen T, Brock JW, Grandjean P. Organochlorine exposure and breast cancer survival. Journal of clinical epidemiology. 2000;53(3):323-330.
17. Hoyer AP, Grandjean P, Jorgensen T, Brock JW, Hartvig HB. Organochlorine exposure and risk of breast cancer. Lancet (London, England). 1998;352(9143):1816-1820.
18. Krieger N, Wolff MS, Hiatt RA, Rivera M, Vogelman J, Orentreich N. Breast cancer and serum organochlorines: a prospective study among white, black, and Asian women. Journal of the National Cancer Institute. 1994;86(8):589-599.
19. Wolff MS, Toniolo PG, Lee EW, Rivera M, Dubin N. Blood levels of organochlorine residues and risk of breast cancer. Journal of the National Cancer Institute. 1993;85(8):648-652.
20. Laden F, Ishibe N, Hankinson SE, Wolff MS, Gertig DM, Hunter DJ, Kelsey KT. Polychlorinated biphenyls, cytochrome P450 1A1, and breast cancer risk in the Nurses' Health Study. Cancer epidemiology, biomarkers & prevention : a publication of the American Association for Cancer Research, cosponsored by the American Society of Preventive Oncology. 2002;11(12):1560-1565.

21. Laden F, Hankinson SE, Wolff MS, Colditz GA, Willett WC, Speizer FE, Hunter DJ. Plasma organochlorine levels and the risk of breast cancer: an extended follow-up in the Nurses' Health Study. International journal of cancer. 2001;91(4):568-574.
22. Hunter DJ, Hankinson SE, Laden F, Colditz GA, Manson JE, Willett WC, Speizer FE, Wolff MS. Plasma organochlorine levels and the risk of breast cancer. The New England journal of medicine. 1997;337(18):1253-1258.
23. Hoyer AP, Jorgensen T, Rank F, Grandjean P. Organochlorine exposures influence on breast cancer risk and survival according to estrogen receptor status: a Danish cohort-nested case-control study. BMC cancer. 2001;1:8.
24. Hoyer AP, Gerdes AM, Jorgensen T, Rank F, Hartvig HB. Organochlorines, p53 mutations in relation to breast cancer risk and survival. A Danish cohort-nested case-controls study. Breast cancer research and treatment. 2002;71(1):59-65.
25. Hoyer AP, Jorgensen T, Grandjean P, Hartvig HB. Repeated measurements of organochlorine exposure and breast cancer risk (Denmark). Cancer causes & control : CCC. 2000;11(2):177-184.
26. Morgan M, Deoraj A, Felty Q, Roy D. Environmental estrogen-like endocrine disrupting chemicals and breast cancer. Molecular and cellular endocrinology. 2017;457:89-102.
27. Xu X, Dailey AB, Talbott EO, Ilacqua VA, Kearney G, Asal NR. Associations of serum concentrations of organochlorine pesticides with breast cancer and prostate cancer in U.S. adults. Environmental health perspectives. 2010;118(1):60-66.
28. Pastor-Barriuso R, Fernandez MF, Castano-Vinyals G, Whelan D, Perez-Gomez B, Llorca J, Villanueva CM, Guevara M, Molina-Molina JM, Artacho-Cordon F; et al. Total Effective Xenoestrogen Burden in Serum Samples and Risk for Breast Cancer in a Population-Based Multicase-Control Study in Spain. Environmental health perspectives. 2016;124(10):1575-1582.
29. Tang M, Zhao M, Zhou S, Chen K, Zhang C, Liu W. Assessing the underlying breast cancer risk of Chinese females contributed by dietary intake of residual DDT from agricultural soils. Environment international. 2014;73:208-215.
30. Gatto NM, Longnecker MP, Press MF, Sullivan-Halley J, McKean-Cowdin R, Bernstein L. Serum organochlorines and breast cancer: a case-control study among African-American women. Cancer causes & control : CCC. 2007;18(1):29-39.
31. Li Y, Millikan RC, Bell DA, Cui L, Tse CK, Newman B, Conway K. Polychlorinated biphenyls, cytochrome P450 1A1 (CYP1A1) polymorphisms, and breast cancer risk among African American women and white women in North Carolina: a population-based case-control study. Breast cancer research : BCR. 2005;7(1):R12-18.
32. Millikan R, DeVoto E, Duell EJ, Tse CK, Savitz DA, Beach J, Edmiston S, Jackson S, Newman B. Dichlorodiphenyldichloroethene, polychlorinated biphenyls, and breast cancer among African-American and white women in North Carolina. Cancer epidemiology, biomarkers & prevention : a publication of the American Association for Cancer Research, cosponsored by the American Society of Preventive Oncology. 2000;9(11):1233-1240.
33. Moysich KB, Shields PG, Freudenheim JL, Schisterman EF, Vena JE, Kostyniak P, Greizerstein H, Marshall JR, Graham S, Ambrosone CB. Polychlorinated biphenyls, cytochrome P4501A1 polymorphism, and postmenopausal breast cancer risk. Cancer epidemiology, biomarkers & prevention : a publication of the American Association for Cancer Research, cosponsored by the American Society of Preventive Oncology. 1999;8(1):41-44.
34. Gammon MD, Wolff MS, Neugut AI, Eng SM, Teitelbaum SL, Britton JA, Terry MB, Levin B, Stellman SD, Kabat GC; et al. Environmental toxins and breast cancer on Long Island. II. Organochlorine compound levels in blood. Cancer epidemiology, biomarkers & prevention : a publication of the American Association for Cancer Research, cosponsored by the American Society of Preventive Oncology. 2002;11(8):686-697.
35. Romieu I, Hernandez-Avila M, Lazcano-Ponce E, Weber JP, Dewailly E. Breast cancer, lactation history, and serum organochlorines. American journal of epidemiology. 2000;152(4):363-370.
36. Arrebola JP, Belhassen H, Artacho-Cordon F, Ghali R, Ghorbel H, Boussen H, Perez-Carrascosa FM, Exposito J, Hedhili A, Olea N. Risk of female breast cancer and serum concentrations of organochlorine pesticides and polychlorinated biphenyls: a case-control study in Tunisia. The Science of the total environment. 2015;520:106-113.
37. Boada LD, Zumbado M, Henriquez-Hernandez LA, Almeida-Gonzalez M, Alvarez-Leon EE, Serra-Majem L, Luzardo OP. Complex organochlorine pesticide mixtures as determinant factor for breast cancer risk: a population-based case-control study in the Canary Islands (Spain). Environmental health : a global access science source. 2012;11:28.
38. Bonefeld-Jorgensen EC, Long M, Bossi R, Ayotte P, Asmund G, Kruger T, Ghisari M, Mulvad G, Kern P, Nzulumiki P; et al. Perfluorinated compounds are related to breast cancer risk in Greenlandic Inuit: a case control study. Environmental health : a global access science source. 2011;10:88.
39. Li JY, Wu DS, Yang F, Zeng HY, Lei FM, Zhou WD, Li H, Tao P. Study on serum organochlorines pesticides (DDTs) level, CYP1A1 genetic polymorphism and risk of breast cancer: a case control study. Zhonghua liu xing bing xue za zhi = Zhonghua liuxingbingxue zazhi. 2006;27(3):217-222.
40. Pavuk M, Cerhan JR, Lynch CF, Kocan A, Petrik J, Chovancova J. Case-control study of PCBs, other organochlorines and breast cancer in Eastern Slovakia. Journal of exposure analysis and environmental epidemiology. 2003;13(4):267-275.
41. Soliman AS, Wang X, DiGiovanni J, Eissa S, Morad M, Vulimiri S, Mahgoub KG, Johnston DA, Do KA, Seifeldin IA; et al. Serum organochlorine levels and history of lactation in Egypt. Environmental research. 2003;92(2):110-117.

42. Dello Iacovo R, Celentano E, Strollo AM, Iazzetta G, Capasso I, Randazzo G. Organochlorines and breast cancer. A study on Neapolitan women. Advances in experimental medicine and biology. 1999;472:57-66.
43. Mendonca GA, Eluf-Neto J, Andrada-Serpa MJ, Carmo PA, Barreto HH, Inomata ON, Kussumi TA. Organochlorines and breast cancer: a case-control study in Brazil. International journal of cancer. 1999;83(5):596-600.
44. Wielsoe M, Kern P, Bonefeld-Jorgensen EC. Serum levels of environmental pollutants is a risk factor for breast cancer in Inuit: a case control study. Environmental health : a global access science source. 2017;16(1):56.
45. Wielsoe M, Eiberg H, Ghisari M, Kern P, Lind O, Bonefeld-Jorgensen EC. Genetic Variations, Exposure to Persistent Organic Pollutants and Breast Cancer Risk - A Greenlandic Case-Control Study. Basic & clinical pharmacology & toxicology. 2018;123(3):335-346.
46. Zhang Y, Wise JP, Holford TR, Xie H, Boyle P, Zahm SH, Rusiecki J, Zou K, Zhang B, Zhu Y; et al. Serum polychlorinated biphenyls, cytochrome P-450 1A1 polymorphisms, and risk of breast cancer in Connecticut women. American journal of epidemiology. 2004;160(12):1177-1183.
47. Demers A, Ayotte P, Brisson J, Dodin S, Robert J, Dewailly E. Risk and aggressiveness of breast cancer in relation to plasma organochlorine concentrations. Cancer epidemiology, biomarkers & prevention : a publication of the American Association for Cancer Research, cosponsored by the American Society of Preventive Oncology. 2000;9(2):161-166.
48. Demers A, Ayotte P, Brisson J, Dodin S, Robert J, Dewailly E. Plasma concentrations of polychlorinated biphenyls and the risk of breast cancer: a congener-specific analysis. American journal of epidemiology. 2002;155(7):629-635.
49. Zheng T, Holford TR, Mayne ST, Tessari J, Ward B, Carter D, Owens PH, Boyle P, Dubrow R, Archibeque-Engle S; et al. Risk of female breast cancer associated with serum polychlorinated biphenyls and 1,1-dichloro-2,2'-bis(p-chlorophenyl)ethylene. Cancer epidemiology, biomarkers & prevention : a publication of the American Association for Cancer Research, cosponsored by the American Society of Preventive Oncology. 2000;9(2):167-174.
50. Holmes AK, Koller KR, Kieszak SM, Sjodin A, Calafat AM, Sacco FD, Varner DW, Lanier AP, Rubin CH. Case-control study of breast cancer and exposure to synthetic environmental chemicals among Alaska Native women. International journal of circumpolar health. 2014;73(1):25760.
51. Zhang H, Liu L, Zhang P, Zhao Y, Wu X, Ni W. [A case-control study on the relationship between organochlorine and female breast cancer]. Wei sheng yan jiu = Journal of hygiene research. 2013;42(1):44-48.
52. Recio-Vega R, Velazco-Rodriguez V, Ocampo-Gomez G, Hernandez-Gonzalez S, Ruiz-Flores P, Lopez-Marquez F. Serum levels of polychlorinated biphenyls in Mexican women and breast cancer risk. Journal of applied toxicology : JAT. 2011;31(3):270-278.
53. Itoh H, Iwasaki M, Hanaoka T, Kasuga Y, Yokoyama S, Onuma H, Nishimura H, Kusama R, Tsugane S. Serum organochlorines and breast cancer risk in Japanese women: a case-control study. Cancer causes & control : CCC. 2009;20(5):567-580.
54. Yang M, Ryu JH, Jeon R, Kang D, Yoo KY. Effects of bisphenol A on breast cancer and its risk factors. Archives of toxicology. 2009;83(3):281-285.
55. Siddiqui MK, Anand M, Mehrotra PK, Sarangi R, Mathur N. Biomonitoring of organochlorines in women with benign and malignant breast disease. Environmental research. 2005;98(2):250-257.
56. Charlier CJ, Albert AI, Zhang L, Dubois NG, Plomteux GJ. Polychlorinated biphenyls contamination in women with breast cancer. Clinica chimica acta; international journal of clinical chemistry. 2004;347(1-2):177-181.
57. Charlier C, Pitance F, Plomteux G. PCB residues in a breast cancer patient population. Bulletin of environmental contamination and toxicology. 2003;71(5):887-891.
58. Charlier C, Foidart JM, Pitance F, Herman P, Gaspard U, Meurisse M, Plomteux G. Environmental dichlorodiphenyltrichlorethane or hexachlorobenzene exposure and breast cancer: is there a risk? Clinical chemistry and laboratory medicine. 2004;42(2):222-227.
59. Rusiecki JA, Holford TR, Zahm SH, Zheng T. Polychlorinated biphenyls and breast cancer risk by combined estrogen and progesterone receptor status. European journal of epidemiology. 2004;19(8):793-801.
60. Charlier C, Albert A, Herman P, Hamoir E, Gaspard U, Meurisse M, Plomteux G. Breast cancer and serum organochlorine residues. Occupational and environmental medicine. 2003;60(5):348-351.
61. Lopez-Carrillo L, Lopez-Cervantes M, Torres-Sanchez L, Blair A, Cebrian ME, Garcia RM. Serum levels of beta-hexachlorocyclohexane, hexachlorobenzene and polychlorinated biphenyls and breast cancer in Mexican women. European journal of cancer prevention : the official journal of the European Cancer Prevention Organisation (ECP). 2002;11(2):129-135.
62. Mathur V, Bhatnagar P, Sharma RG, Acharya V, Sexana R. Breast cancer incidence and exposure to pesticides among women originating from Jaipur. Environment international. 2002;28(5):331-336.
63. Ahmed MT, Loutfy N, El Shiekh E. Residue levels of DDE and PCBs in the blood serum of women in the Port Said region of Egypt. Journal of hazardous materials. 2002;89(1):41-48.
64. Wolff MS, Berkowitz GS, Brower S, Senie R, Bleiweiss IJ, Tartter P, Pace B, Roy N, Wallenstein S, Weston A. Organochlorine exposures and breast cancer risk in New York City women. Environmental research. 2000;84(2):151-161.

65. Burger M, Mate M, Lavina R, Carzoglio J, Antonaz R, Rampoldi O. Role of the organochlorine pesticides in breast cancer. Revista de Toxicologia. 2000;17(2):79-82.
66. Olaya-Contreras P, Rodriguez-Villamil J, Posso-Valencia HJ, Cortez JE. Organochlorine exposure and breast cancer risk in Colombian women. Cadernos de saude publica. 1998;14 Suppl 3:125-132.
67. Lopez-Carrillo L, Blair A, Lopez-Cervantes M, Cebrian M, Rueda C, Reyes R, Mohar A, Bravo J. Dichlorodiphenyltrichloroethane serum levels and breast cancer risk: a case-control study from Mexico. Cancer research. 1997;57(17):3728-3732.
68. Schecter A, Toniolo P, Dai LC, Thuy LT, Wolff MS. Blood levels of DDT and breast cancer risk among women living in the north of Vietnam. Archives of environmental contamination and toxicology. 1997;33(4):453-456.
69. Dewailly E, Dodin S, Verreault R, Ayotte P, Sauve L, Morin J, Brisson J. High organochlorine body burden in women with estrogen receptor-positive breast cancer. Journal of the National Cancer Institute. 1994;86(3):232-234.
70. Ye HZ. A case-control study on the relationship between organochlorine and breast cancer: Zhejiang University; 2009.
71. Sprague BL, Trentham-Dietz A, Hedman CJ, Wang J, Hemming JD, Hampton JM, Buist DS, Aiello Bowles EJ, Sisney GS, Burnside ES. Circulating serum xenoestrogens and mammographic breast density. Breast cancer research : BCR. 2013;15(3):R45.
72. Diorio C, Dumas I, Sandanger TM, Ayotte P. Levels of circulating polychlorinated biphenyls and mammographic breast density. Anticancer research. 2013;33(12):5483-5489.
73. He Y, Peng L, Zhang W, Liu C, Yang Q, Zheng S, Bao M, Huang Y, Wu K. Adipose tissue levels of polybrominated diphenyl ethers and breast cancer risk in Chinese women: A case-control study. Environmental research. 2018;167:160-168.
74. He Y, Peng L, Huang Y, Peng X, Zheng S, Liu C, Wu K. Association of breast adipose tissue levels of polychlorinated biphenyls and breast cancer development in women from Chaoshan, China. Environmental science and pollution research international. 2017;24(5):4778-4790.
75. He TT, Zuo AJ, Wang JG, Zhao P. Organochlorine pesticides accumulation and breast cancer: A hospital-based case-control study. Tumour biology : the journal of the International Society for Oncodevelopmental Biology and Medicine. 2017;39(5):1010428317699114.
76. Ociepa-Zawal M, Rubis B, Wawrzynczak D, Wachowiak R, Trzeciak WH. Accumulation of environmental estrogens in adipose tissue of breast cancer patients. Journal of environmental science and health Part A, Toxic/hazardous substances & environmental engineering. 2010;45(3):305-312.
77. Hurley S, Reynolds P, Goldberg D, Nelson DO, Jeffrey SS, Petreas M. Adipose levels of polybrominated diphenyl ethers and risk of breast cancer. Breast cancer research and treatment. 2011;129(2):505-511.
78. Reynolds P, Hurley SE, Petreas M, Goldberg DE, Smith D, Gilliss D, Mahoney ME, Jeffrey SS. Adipose levels of dioxins and risk of breast cancer. Cancer causes & control : CCC. 2005;16(5):525-535.
79. Cassidy RA, Natarajan S, Vaughan GM. The link between the insecticide heptachlor epoxide, estradiol, and breast cancer. Breast cancer research and treatment. 2005;90(1):55-64.
80. Waliszewski SM, Bermudez MT, Infanzon RM, Silva CS, Carvajal O, Trujillo P, Gomez Arroyo S, Villalobos Pietrini R, Saldana VA, Melo G; et al. Persistent organochlorine pesticide levels in breast adipose tissue in women with malignant and benign breast tumors. Bulletin of environmental contamination and toxicology. 2005;75(4):752-759.
81. McCready D, Aronson KJ, Chu W, Fan W, Vesprini D, Narod SA. Breast tissue organochlorine levels and metabolic genotypes in relation to breast cancer risk Canada. Cancer causes & control : CCC. 2004;15(4):399-418.
82. Ibarluzea Jm J, Fernandez MF, Santa-Marina L, Olea-Serrano MF, Rivas AM, Aurrekoetxea JJ, Exposito J, Lorenzo M, Torne P, Villalobos M; et al. Breast cancer risk and the combined effect of environmental estrogens. Cancer causes & control : CCC. 2004;15(6):591-600.
83. Lucena RA, Allam MF, Costabeber IH, Villarejo ML, Navajas RF. Breast cancer risk factors: PCB congeners. European journal of cancer prevention : the official journal of the European Cancer Prevention Organisation (ECP). 2001;10(1):117-119.
84. Charles MJ, Schell MJ, Willman E, Gross HB, Lin Y, Sonnenberg S, Graham ML. Organochlorines and 8-hydroxy-2'-deoxyguanosine (8-OHdG) in cancerous and noncancerous breast tissue: do the data support the hypothesis that oxidative DNA damage caused by organochlorines affects breast cancer? Archives of environmental contamination and toxicology. 2001;41(3):386-395.
85. Woolcott CG, Aronson KJ, Hanna WM, SenGupta SK, McCready DR, Sterns EE, Miller AB. Organochlorines and breast cancer risk by receptor status, tumor size, and grade (Canada). Cancer causes & control : CCC. 2001;12(5):395-404.
86. Aronson KJ, Miller AB, Woolcott CG, Sterns EE, McCready DR, Lickley LA, Fish EB, Hiraki GY, Holloway C, Ross T; et al. Breast adipose tissue concentrations of polychlorinated biphenyls and other organochlorines and breast cancer risk. Cancer epidemiology, biomarkers & prevention : a publication of the American Association for Cancer Research, cosponsored by the American Society of Preventive Oncology. 2000;9(1):55-63.

87. Zheng T, Holford TR, Tessari J, Mayne ST, Zahm SH, Owens PH, Zhang B, Ward B, Carter D, Zhang Y; et al. Oxychlordane and trans-nonachlor in breast adipose tissue and risk of female breast cancer. Journal of epidemiology and biostatistics. 2000;5(3):153-160.
88. Zheng T, Holford TR, Tessari J, Mayne ST, Owens PH, Ward B, Carter D, Boyle P, Dubrow R, Archibeque-Engle S; et al. Breast cancer risk associated with congeners of polychlorinated biphenyls. American journal of epidemiology. 2000;152(1):50-58.
89. Zheng T, Holford TR, Mayne ST, Ward B, Carter D, Owens PH, Dubrow R, Zahm SH, Boyle P, Archibeque S; et al. DDE and DDT in breast adipose tissue and risk of female breast cancer. American journal of epidemiology. 1999;150(5):453-458.
90. Zheng T, Holford TR, Mayne ST, Owens PH, Ward B, Carter D, Dubrow R, Zahm SH, Boyle P, Tessari J. Beta-benzene hexachloride in breast adipose tissue and risk of breast carcinoma. Cancer. 1999;85(10):2212-2218.
91. Zheng T, Holford TR, Mayne ST, Tessari J, Owens PH, Zahm SH, Zhang B, Dubrow R, Ward B, Carter D; et al. Environmental exposure to hexachlorobenzene (HCB) and risk of female breast cancer in Connecticut. Cancer epidemiology, biomarkers & prevention : a publication of the American Association for Cancer Research, cosponsored by the American Society of Preventive Oncology. 1999;8(5):407-411.
92. Holford TR, Zheng T, Mayne ST, Zahm SH, Tessari JD, Boyle P. Joint effects of nine polychlorinated biphenyl (PCB) congeners on breast cancer risk. International journal of epidemiology. 2000;29(6):975-982.
93. Stellman SD, Djordjevic MV, Britton JA, Muscat JE, Citron ML, Kemeny M, Busch E, Gong L. Breast cancer risk in relation to adipose concentrations of organochlorine pesticides and polychlorinated biphenyls in Long Island, New York. Cancer epidemiology, biomarkers & prevention : a publication of the American Association for Cancer Research, cosponsored by the American Society of Preventive Oncology. 2000;9(11):1241-1249.
94. Bagga D, Anders KH, Wang HJ, Roberts E, Glaspy JA. Organochlorine pesticide content of breast adipose tissue from women with breast cancer and control subjects. Journal of the National Cancer Institute. 2000;92(9):750-753.
95. Liljegren G, Hardell L, Lindstrom G, Dahl P, Magnuson A. Case-control study on breast cancer and adipose tissue concentrations of congener specific polychlorinated biphenyls, DDE and hexachlorobenzene. European journal of cancer prevention : the official journal of the European Cancer Prevention Organisation (ECP). 1998;7(2):135-140.
96. Guttes S, Failing K, Neumann K, Kleinstein J, Georgii S, Brunn H. Chlororganic pesticides and polychlorinated biphenyls in breast tissue of women with benign and malignant breast disease. Archives of environmental contamination and toxicology. 1998;35(1):140-147.
97. Hardell L, Lindstrom G, Liljegren G, Dahl P, Magnuson A. Increased concentrations of octachlorodibenzo-p-dioxin in cases with breast cancer--results from a case-control study. European journal of cancer prevention : the official journal of the European Cancer Prevention Organisation (ECP). 1996;5(5):351-357.
98. Djordjevic MV, Hoffmann D, Fan J, Prokopczyk B, Citron ML, Stellman SD. Assessment of chlorinated pesticides and polychlorinated biphenyls in adipose breast tissue using a supercritical fluid extraction method. Carcinogenesis. 1994;15(11):2581-2585.
99. Falck F, Jr., Ricci A, Jr., Wolff MS, Godbold J, Deckers P. Pesticides and polychlorinated biphenyl residues in human breast lipids and their relation to breast cancer. Archives of environmental health. 1992;47(2):143-146.
100. Mussalo-Rauhamaa H, Hasanen E, Pyysalo H, Antervo K, Kauppila R, Pantzar P. Occurrence of beta-hexachlorocyclohexane in breast cancer patients. Cancer. 1990;66(10):2124-2128.
101. Unger M, Kiaer H, Blichert-Toft M, Olsen J, Clausen J. Organochlorine compounds in human breast fat from deceased with and without breast cancer and in a biopsy material from newly diagnosed patients undergoing breast surgery. Environmental research. 1984;34(1):24-28.
102. Wassermann M, Nogueira DP, Tomatis L, Mirra AP, Shibata H, Arie G, Cucos S, Wassermann D. Organochlorine compounds in neoplastic and adjacent apparently normal breast tissue. Bulletin of environmental contamination and toxicology. 1976;15(4):478-484.
103. Brauner EV, Loft S, Wellejus A, Autrup H, Tjonneland A, Raaschou-Nielsen O. Adipose tissue PCB levels and CYP1B1 and COMT genotypes in relation to breast cancer risk in postmenopausal Danish women. International journal of environmental health research. 2014;24(3):256-268.
104. Raaschou-Nielsen O, Pavuk M, Leblanc A, Dumas P, Philippe Weber J, Olsen A, Tjonneland A, Overvad K, Olsen JH. Adipose organochlorine concentrations and risk of breast cancer among postmenopausal Danish women. Cancer epidemiology, biomarkers & prevention : a publication of the American Association for Cancer Research, cosponsored by the American Society of Preventive Oncology. 2005;14(1):67-74.
105. van't Veer P, Lobbezoo IE, Martin-Moreno JM, Guallar E, Gomez-Aracena J, Kardinaal AF, Kohlmeier L, Martin BC, Strain JJ, Thamm M; et al. DDT (dicophane) and postmenopausal breast cancer in Europe: case-control study. BMJ (Clinical research ed). 1997;315(7100):81-85.

106. Parada H, Jr., Wolff MS, Engel LS, White AJ, Eng SM, Cleveland RJ, Khankari NK, Teitelbaum SL, Neugut AI, Gammon MD. Organochlorine insecticides DDT and chlordane in relation to survival following breast cancer. International journal of cancer. 2016;138(3):565-575.
107. Parada H, Jr., Wolff MS, Engel LS, Eng SM, Khankari NK, Neugut AI, Teitelbaum SL, Gammon MD. Polychlorinated biphenyls and their association with survival following breast cancer. European journal of cancer (Oxford, England : 1990). 2016;56:21-30.
108. Muscat JE, Britton JA, Djordjevic MV, Citron ML, Kemeny M, Busch-Devereaux E, Pittman B, Stellman SD. Adipose concentrations of organochlorine compounds and breast cancer recurrence in Long Island, New York. Cancer epidemiology, biomarkers & prevention : a publication of the American Association for Cancer Research, cosponsored by the American Society of Preventive Oncology. 2003;12(12):1474-1478.
109. Roswall N, Sorensen M, Tjonneland A, Raaschou-Nielsen O. Organochlorine concentrations in adipose tissue and survival in postmenopausal, Danish breast cancer patients. Environmental research. 2018;163:237-248.
110. Cocco P, Kazerouni N, Zahm SH. Cancer mortality and environmental exposure to DDE in the United States. Environmental health perspectives. 2000;108(1):1-4.
111. Arrebola JP, Fernandez-Rodriguez M, Artacho-Cordon F, Garde C, Perez-Carrascosa F, Linares I, Tovar I, Gonzalez-Alzaga B, Exposito J, Torne P; et al. Associations of persistent organic pollutants in serum and adipose tissue with breast cancer prognostic markers. The Science of the total environment. 2016;566-567:41-49.
112. Charlier CJ, Dejardin MT. Increased risk of relapse after breast cancer with exposure to organochlorine pollutants. Bulletin of environmental contamination and toxicology. 2007;78(1):1-4.
113. Ellsworth RE, Kostyniak PJ, Chi LH, Shriver CD, Costantino NS, Ellsworth DL. Organochlorine pesticide residues in human breast tissue and their relationships with clinical and pathological characteristics of breast cancer. Environmental toxicology. 2018.
114. Eldakroory SA, Morsi DE, Abdel-Rahman RH, Roshdy S, Gouida MS, Khashaba EO. Correlation between toxic organochlorine pesticides and breast cancer. Human & experimental toxicology. 2017;36(12):1326-1334.
115. Barr L, Metaxas G, Harbach CA, Savoy LA, Darbre PD. Measurement of paraben concentrations in human breast tissue at serial locations across the breast from axilla to sternum. Journal of applied toxicology : JAT. 2012;32(3):219-232.
116. Munoz-de-Toro M, Durando M, Beldomenico PM, Beldomenico HR, Kass L, Garcia SR, Luque EH. Estrogenic microenvironment generated by organochlorine residues in adipose mammary tissue modulates biomarker expression in ERalpha-positive breast carcinomas. Breast cancer research : BCR. 2006;8(4):R47.
